# Zn/Cd status-dependent accumulation of Zn and Cd in root parts in tobacco is accompanied by specific expression of *ZIP* genes

**DOI:** 10.1186/s12870-020-2255-3

**Published:** 2020-01-22

**Authors:** Małgorzata Palusińska, Anna Barabasz, Katarzyna Kozak, Anna Papierniak, Karolina Maślińska, Danuta Maria Antosiewicz

**Affiliations:** University of Warsaw, Faculty of Biology, Institute of Experimental Plant Biology and Biotechnology, Miecznikowa Street 1, 02-096 Warszawa, Poland

**Keywords:** Cadmium, Metal uptake, *NtZIP5B*, Root-to-shoot translocation, Tobacco (*Nicotiana tabacum*), Zinc

## Abstract

**Background:**

Root-to-shoot translocation of zinc (Zn) and cadmium (Cd) depends on the concentrations of both metals in the medium. A previous study on tobacco (*Nicotiana tabacum*) pointed to the contribution of *NtZIP1, NtZIP2, NtZIP4* and *NtIRT1-like* in the regulation of this phenomenon. To learn more, Zn and Cd accumulation, root/shoot distribution and the expression of *ZIP* genes were investigated in the apical, middle and basal root parts.

**Results:**

We show that Zn/Cd status-dependent root-shoot distribution of both metals was related to distinct metal accumulation in root parts. At low Zn and Cd in the medium, the apical part contained the highest metal level; at higher concentrations, the middle and basal parts were the major sink for excess metal. The above were accompanied by root part-specific expression pattern modifications of *ZIPs* (*NtZIP1-like, NtZIP2, NtZIP4A/B, NtZIP5A/B, NtZIP5-like, NtZIP8, NtZIP11, NtIRT1*, and *NtIRT1-like*) that fell into four categories with respect to the root part. Furthermore, for lower Zn/Cd concentrations changes were noted for *NtZIP5A/B* and *NtZIP5-like* only*,* but at higher Zn and Cd levels for *NtZIP1-like, NtZIP5-like, NtZIP8, NtZIP11, NtIRT1*, and *NtIRT1-like. NtZIP1,* here renamed to *NtZIP5B*, was cloned and characterized. We found that it was a zinc deficiency-inducible transporter involved in zinc and cadmium uptake from the soil solution primarily by the middle root part.

**Conclusions:**

We conclude that regulation of the longitudinal distribution of Zn and Cd is highly specific, and that the apical, middle and basal root parts play distinct roles in Zn/Cd status-dependent control of metal translocation efficiency to shoots, including the stimulation of Zn translocation to shoots in the presence of Cd. These results provide new insight into the root part-specific unique role of *NtZIP5B* and other *ZIP* genes in the longitudinal distribution of zinc and cadmium and their contribution to the regulation of root-to-shoot translocation.

## Background

Tobacco is known to accumulate metals (including Zn and Cd) in aerial parts more efficiently than many other species [1, 2]. It has, therefore, been frequently used for phytoextraction purposes [3, 4]. On the other hand, these characteristics put smokers at risk of being exposed to cadmium accumulated in shoots. Thus, in-depth knowledge of the molecular mechanisms governing the rate of translocation of metals to shoots might generate future biotechnology-based modifications in Zn/Cd accumulation in the aerial parts of tobacco plants.

The regulation pathways of Zn homeostasis are not uniquely specific for this metal. Conversely, its regulation via common pathways is closely related to the homeostasis of other metals, as well as some toxic metals including Cd. This has been termed metal cross-homeostasis. It is based on one metal transport gene being regulated by more than one metal, and on metal transport proteins or chelating compounds having diverse substrate specificities [5–7]. There are strong competitive interactions between Zn and Cd. At the whole plant level these relationships are manifested, among others, by changes in Zn and Cd uptake and translocation to shoots in a manner dependent on the concentration of both metals in the medium [8, 9]. For example, excess Zn affects the uptake and root/shoot distribution of Cd. In *A. halleri* or *T. caerulescens* (current name, *Noccaea caerulescens)*, Cd transfer to shoots was competitively inhibited by Zn [10, 11]. The presence of Cd in the medium/soil also perturbs the uptake and translocation of Zn. Reduction of Zn transfer to shoots in the presence of Cd was reported, e.g., in *N. rustica* and *T. caerulescence* [12, 13], however, no changes were observed in tumbleweed or in *A. thaliana* [14, 15].

Despite their common occurrence, the mechanisms underlying this phenomenon have been only fragmentarily depicted. To learn more about the regulation of Zn/Cd supply-dependent root-to-shoot translocation of these metals in tobacco, the aim of this study was to examine the possible contribution of *ZIP* genes (encoding proteins belonging to the family of ZRT\IRT-related proteins) to the root/shoot distribution of both metals under different metal concentrations in the medium. The focus on *ZIP* genes results from SSH (Suppression Subtractive Hybridization)-based identification of tobacco *NtZIP1, NtZIP2, NtZIP4* and *IRT1-like* as genes having different expression under combinations of low to high Zn/Cd concentrations in the medium. This accompanied changes in the root/shoot partitioning of both metals [9]. Proteins from the ZIP family mediate the transport of a range of metals, including Zn and Cd, to the cytoplasm either from the extracellular space or from internal stores [16]. In *Arabidopsis*, AtZIP1 (localized to the tonoplast) is involved in Zn and Mn transport, and Zn- and Fe-deficiency upregulated its expression [17]. The ability to transport Cd was detected, e.g., for MtZIP1 [18] and OsZIP1 [19] but not for the new tobacco *NtZIP1-like* and *NtZIP11* genes encoding a Zn- (but not Cd- or Fe-) uptake protein [20, 21]. AtZIP2 from *A. thaliana* and MtZIP2 from *Medicago truncatula* are Zn uptake proteins localized to the plasma membrane, although AtZIP2 is also able to transport Mn, Cu, and potentially Cd [17, 22–24]. For comparison, the ability to transport Zn and Cd has also been shown for NtZIP4 [25], MnZIP4 (from *Morus notabilis*) [26], AtIRT1 [27, 28], and PsIRT1 (from *Pisum sativum*) [29].

In this study, we focused on root-specific processes associated with changes in Zn and Cd root/shoot partitioning and in the expression pattern of *ZIP* genes that depended on combinations of low and high concentrations of both metals in the medium. For the first time, analysis was performed not on whole roots, but on root parts (apical, middle, basal). Here, nine *ZIP* genes (*NtZIP1-like, NtZIP2, NtZIP4A/B, NtZIP5A/B, NtZIP5-like, NtZIP8, NtZIP11, NtIRT1*, and *NtIRT1-like*) were included in the analysis. Furthermore, as the result of detailed analysis *NtZIP1* was renamed *NtZIP5,* and two copies, *NtZIP5A* and *NtZIP5B*, were identified. *NtZIP5B* was cloned and functionally characterized. Our results provide new insights into the role of three root parts in Zn/Cd status-dependent accumulation of Zn and Cd and into the expression of *ZIP* genes associated with distinct Zn and Cd root/shoot partition patterns.

## Results

### Zn/Cd supply-dependent root/shoot metal distribution

**Fig. 1:**
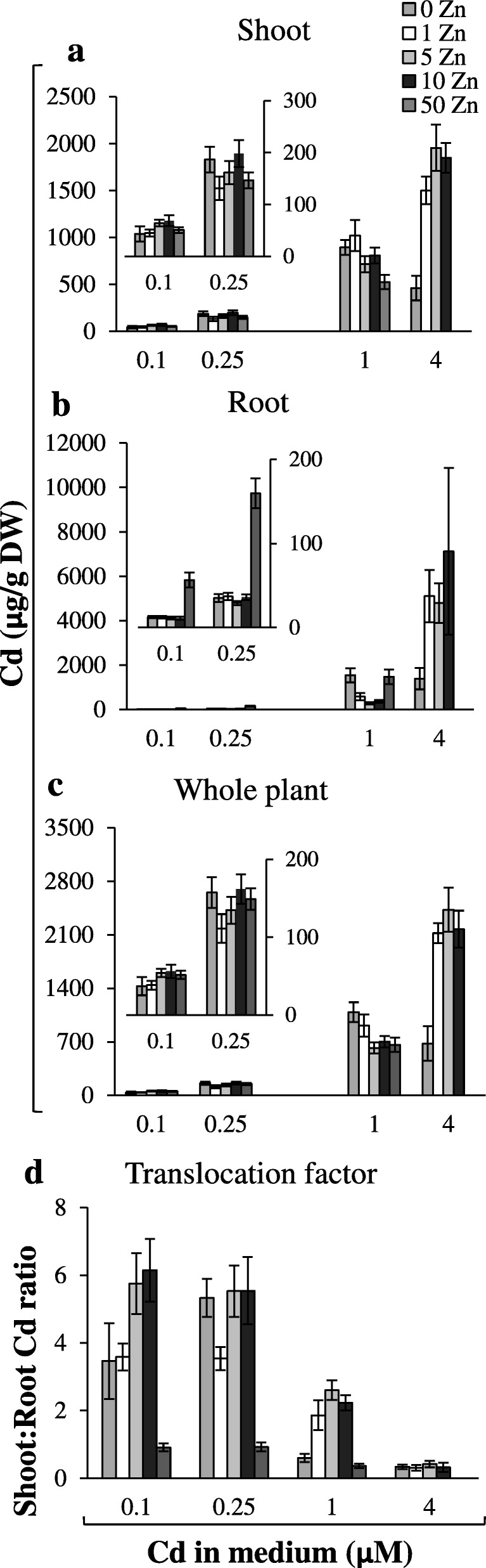
Cadmium concentration in plants grown under various Zn and Cd concentrations. 3.5-week old plants grown in the quarter-strength Knop’s medium were exposed for 17 days to the control medium supplemented with pairwise combinations of Zn (0; 1; 5; 10; 50 μM) and Cd (0; 0.25; 1; 4 μM) concentrations. Concentrations of metals were determined in the shoots (**a**), roots (**b**) and whole plants (**c**). The Translocation Factor (TF) was determined as the ratio of shoot/root concentrations of Cd (**d**). Concentrations of Cd were not measured for plants grown at the combination 4 μM Cd + 50 μM Zn since it was too toxic. Inserts in the (**a**, **b**, **c**) represent parts of the graph with decreased scale at the Y axis. Values correspond to arithmetic means ±SD (*n* = 10). Significance of differences between all values were evaluated by Student’s *t*-test (*P* ≤ 0.05), and it has been determined that each pair of values is significantly different if the SD bars do not overlap. Significance has not been marked above the bars with the letters or arrows to keep the clarity of the graph

**Fig. 2:**
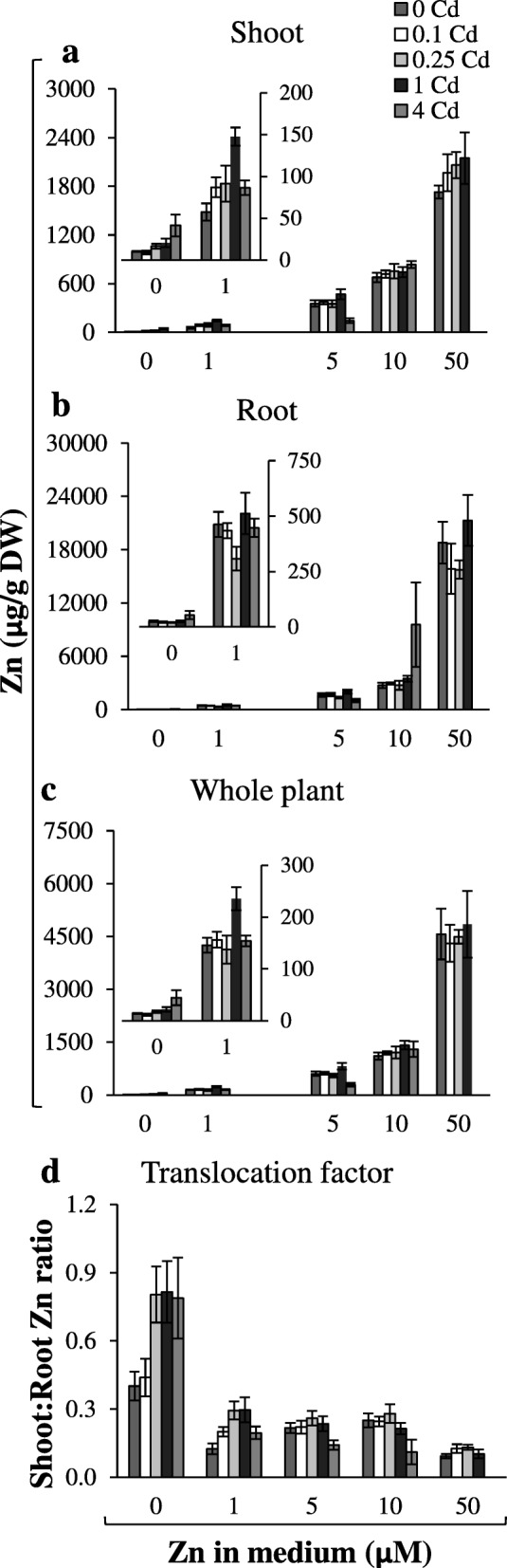
Zinc concentration in plants grown under various Zn and Cd concentrations. 3.5-week old plants grown in the quarter-strength Knop’s medium were exposed for 17 days to the control medium supplemented with pairwise combinations of Zn (0; 1; 5; 10; 50 μM) and Cd (0; 0.25; 1; 4 μM) concentrations. Concentrations of metals were determined in the shoots (**a**), roots (**b**) and whole plants (**c**). The Translocation Factor (TF) was determined as the ratio of shoot/root concentrations of Zn (**d**). Concentrations of Zn were not measured for plants grown at the combination 4 μM Cd + 50 μM Zn since it was too toxic. Inserts in the (**a**, **b**, **c**) represent parts of the graph with decreased scale at the Y axis. Values correspond to arithmetic means ±SD (n = 10). Significance of differences between all values were evaluated by Student’s *t*-test (P ≤ 0.05), and it has been determined that each pair of values is significantly different if the SD bars do not overlap. Significance has not been marked above the bars with the letters or arrows to keep the clarity of the graph

The aim of this study was to determine to what extent the levels of Zn and Cd in the medium affect the root-to-shoot translocation of both metals. It was shown that in plants exposed to a range of Zn (0; 1; 5; 10; 50 μM) and Cd (0; 0.1; 0.25; 1; 4 μM) concentrations for 17 days, Zn/Cd accumulation and translocation efficiency depended on the pairwise combination of both metals (Figs. 1-2; Additional file 1).

As the Cd concentration in the medium increased (regardless of the accompanying Zn level), the enhanced concentration of Cd in plants (Fig. 1a-c) was accompanied by decreased efficiency of root-to-shoot translocation (Fig. 1d). Concentrations of Cd in the shoots of plants grown at each tested Cd level were modified by accompanying Zn concentrations (Fig. 1a, b). Interestingly, at 0 Zn/4 μM Cd, the concentration of Cd in the shoots was 3- to 4-times lower compared with variants containing Zn (Fig. 1a); this did not result from a decreased translocation rate (Fig. 1d), but likely from lower Cd uptake in the absence of Zn under Zn deficiency (Fig. 1c). Moreover, the presence of 50 μM Zn reduced Cd translocation (Fig. 1d), and the accompanying high level of Cd in roots (Fig. 1b) indicated enhanced retention of Cd in this organ.

The presence of Cd also modified Zn shoot/root distribution (Fig. 2a-d). The pattern, however, was not uniform. In the presence of high (4 μM) Cd, Zn translocation to shoots was reduced (relative to the medium without Cd), however, only for plants exposed to 5–50 μM Zn. Notably, the opposite effect, an increase in Zn translocation in the presence of 0.25 and 1 μM Cd was observed in plants grown at low/medium Zn concentrations (0 to 1 μM) (Fig. 2d).

### The effects of long-term exposure of zinc and cadmium on Cd-dependent stimulation of Zn translocation to shoots

**Fig. 3:**
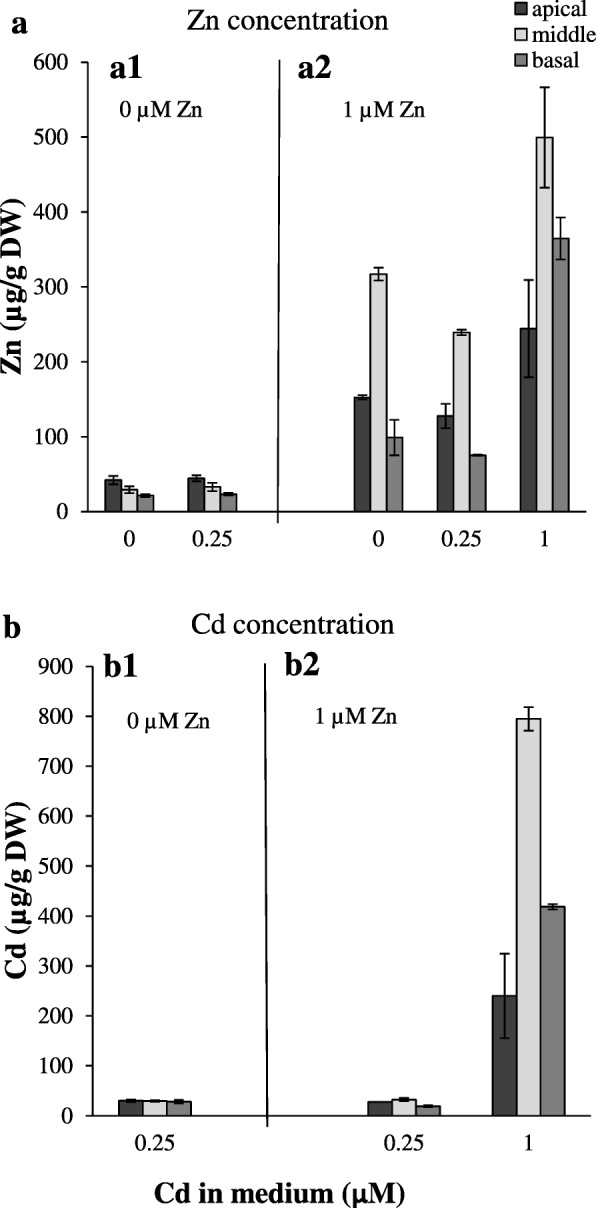
Zinc and cadmium concentration in the apical, middle and basal parts of roots from plants grown under various Zn and Cd concentrations. 3.5-week old plants grown in the quarter-strength Knop’s medium were exposed to the control medium supplemented with pairwise combinations of Zn (0; 1 μM) and Cd (0; 0.25; 1 μM) concentrations for 17 days. Concentrations of metals were measured in the apical parts (2.5 cm) from the main and lateral roots; basal (proximal) parts (1/4 of the total length of the main roots); middle part (remaining parts of the main roots). The basal and the middle parts of the roots were collected from the main root only (adventitious roots were excised). Zinc concentration (**a**): at Zn-deficiency (a1), at 1 μM Zn (a2). Cadmium concentration (**b**): at Zn-deficiency (b1), at 1 μM Zn (b2). Values correspond to arithmetic means ±SD (n = 10). Significance of differences between all values were evaluated by Student’s *t*-test (P ≤ 0.05), and it has been determined that each pair of values is significantly different if the SD bars do not overlap. Significance has not been marked above the bars with the letters or arrows to keep the clarity of the graph

Next, the study focused on Zn/Cd combinations at which Cd-dependent stimulation of Zn root-to-shoot translocation was detected (0 Zn + 0.25 μM Cd; 1 μM Zn + 0.25 μM Cd; 1 μM Zn + 1 μM Cd vs medium without Cd). The efficiency of retention of a metal in roots is one of the critical factors in controlling the rate of its root-to-shoot transfer. Depending on the combination of Zn/Cd concentrations in the medium, different root parts dominated in the accumulation of Zn and Cd. Under Zn deficiency the distribution of Zn among the apical, middle and basal root parts of plants grown without Cd and in the presence of 0.25 μM Cd was similar, with the highest Zn concentration found in the apical parts (Fig. 3a1). The Zn distribution changed at higher Zn and Cd status, and at 1 μM Zn + 1 μM Cd the middle and basal parts became the major root storage sites for both Zn and Cd (Fig. 3a2, b2). At lower Zn/Cd concentrations in the medium (0 Zn + 0.25 μM Cd), accumulation of Cd remained at the same level in three root parts (Fig. 3b1). These results point to the importance of the apical parts in Zn absorption under Zn deficiency, while at higher metal concentrations the basal part contributes more efficiently to metal accumulation.

**Fig. 4:**
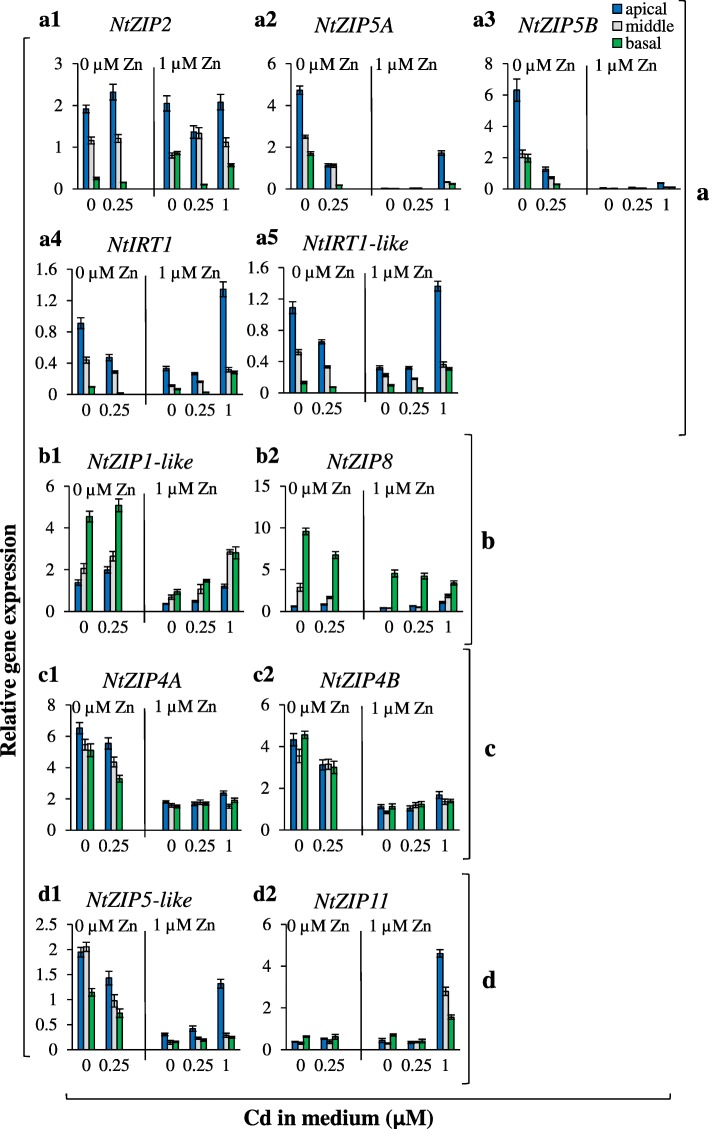
Expression of tobacco *ZIP* genes in the apical, middle and basal parts of roots from plants grown under various Zn and Cd concentrations. 3.5-week old plants grown in the quarter-strength Knop’s medium were exposed to the control medium supplemented with pairwise combinations of Zn (0; 1 μM) and Cd (0; 0.25; 1 μM) for 17 days. *ZIP* genes preferentially expressed in the apical root part (**a**): *NtZIP2* (a1), *NtZIP5A* (a2), *NtZIP5B* (a3), *NtIRT1* (a4), *NtIRT1-like* (a5); in the basal root part (**b**): *NtZIP1-like* (b1), *NtZIP8* (b2); equally expressed in tested three root parts (**c**): *NtZIP4A* (c1), *NtZIP4B* (c2); mixed expression pattern for tested three root parts (**d**): *NtZIP5-like* (d1)*, NtZIP11* (d2). Gene expression was normalized to the *PP2A* level. Values correspond to arithmetic means ±SD (*n* = 3); those with the ratio greater than 2 are considered significantly different

The expression of nine *ZIP* genes was determined under the above conditions. Differences among the apical, middle, and basal root parts allowed categorization of *ZIP* genes into four groups (Fig. 4 a-d). The first contains *ZIPs* with the highest expression in the apical part (*NtZIP2, NtZIP5A/B, NtIRT1*, *NtIRT1-like*) (Fig. 4a1-a5). The second comprises *NtZIP1-like* and *NtZIP8* expressed preferentially in the basal root part (Fig. 4b1-b2). The third is represented by *NtZIP4*A/B with equal expression in the three root parts (Fig. 4c1-c2). The remaining *NtZIP5-like* and *NtZIP11* fall into the fourth group characterized by intermediate expression that changed in different ways in the root parts under the tested combinations of Zn/Cd concentrations (Fig. 4d1-d2).

Furthermore, the transcript level of each tested *ZIP* gene in the apical, middle and basal root part depended on the combinations of Zn/Cd in the medium, although to different extents (Fig. 4). Expression was modified by Zn availability (all *ZIPs*, except *NtZIP2* and *NtZIP11,* were upregulated by Zn-deficiency), and by the Zn/Cd concentration ratios. Here we investigated the root part-specific expression of *ZIPs* that accompanied Cd-dependent stimulation of Zn root-to-shoot translocation detected at different combinations of Zn to Cd concentrations: 0.25 μM Cd + 0 Zn, 0.25 μM Cd + 1 μM Zn, 1 μM Cd + 1 μM Zn (Fig. 2d). At each of these three combinations, different *ZIP* expression patterns were found. Adding 0.25 μM Cd to the Zn-deficient medium decreased expression of *NtZIP5A/B* in all three root parts (Fig. 4a2-a3,) *NtIRT1* in the apical (Fig. 4a4) and *NtZIP5*-like in the middle part (Fig. 4d1), but its presence in the medium containing 1 μM Zn resulted in lower expression of *NtZIP2* in the basal root part only (Fig. 4a1). Furthermore, at the combination of 1 μM Cd + 1 μM Zn (relative to 0 μM Cd + 1 μM Zn) increased transcript levels were noted either in all three root parts (*NtZIP11*; Fig. 4d2), in the apical and middle parts (*NtZIP8*, Fig. 4b2), in the basal and middle parts (*NtZIP1*-like; Fig. 4b1), or in the apical part only (*NtZIP5-like*, *NtIRT1-like, NtIRT1, NtZIP5A*) (Fig. 4d1, a5, a4, a2, respectively).

The detected root part-specific changes indicate a distinct function of each part in mineral nutrition at low/sufficient Zn in the medium and in the presence/absence of toxic Cd, in which the *ZIPs* under study play specific roles.

### Short-term exposure to Cd: expression and accumulation study

**Fig. 5:**
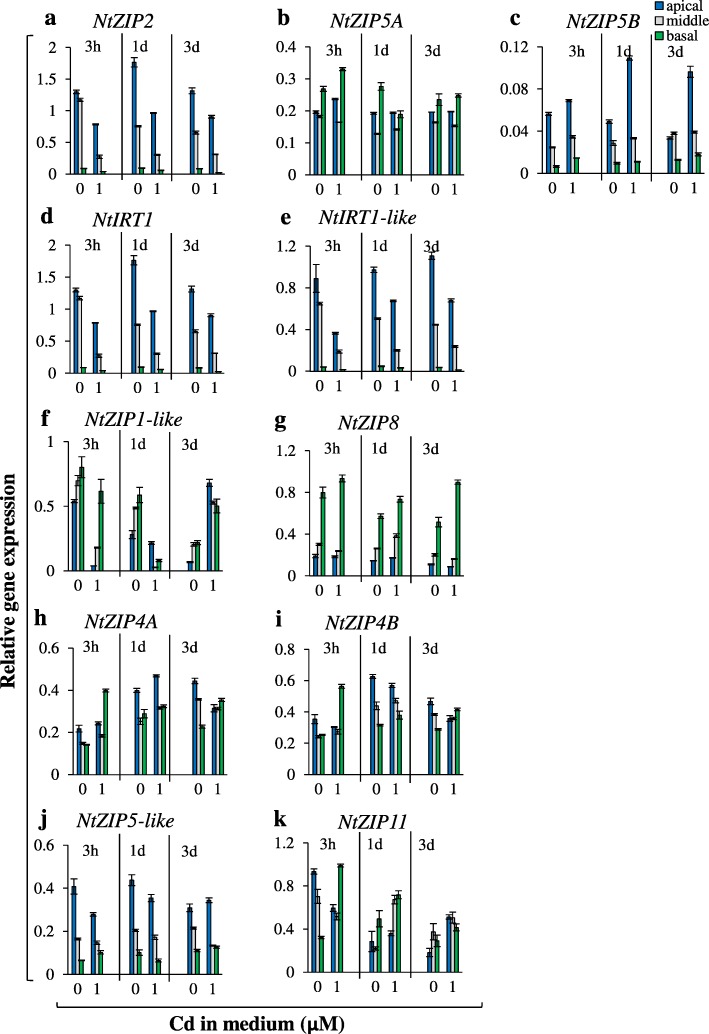
Expression of *ZIP* genes in the apical, middle and basal parts of roots from plants after short-term exposure to Cd. 5.5-week old plants grown in the quarter-strength Knop’s medium were exposed to the control medium containing 1 μM Zn with (1 μM Cd) or without Cd for 3 h, 1 day, or 3 days. Expression of *NtZIP2A* (**a**), *NtZIP5A* (**b**), *NtZIP5B* (**c**), *NtIRT1* (**d**), *NtIRT1-like* (**e**), *NtZIP1-like* (**f**), *NtZIP8* (**g**), *NtZIP4A* (**h**), *NtZIP4B* (**i**), *NtZIP5-like* (**j**), *NtZIP11* (**k**). Gene expression was normalized to the *PP2A* level. Values correspond to arithmetic means ±SD (n = 3); those with the ratio greater than 2 are considered significantly different

**Fig. 6:**
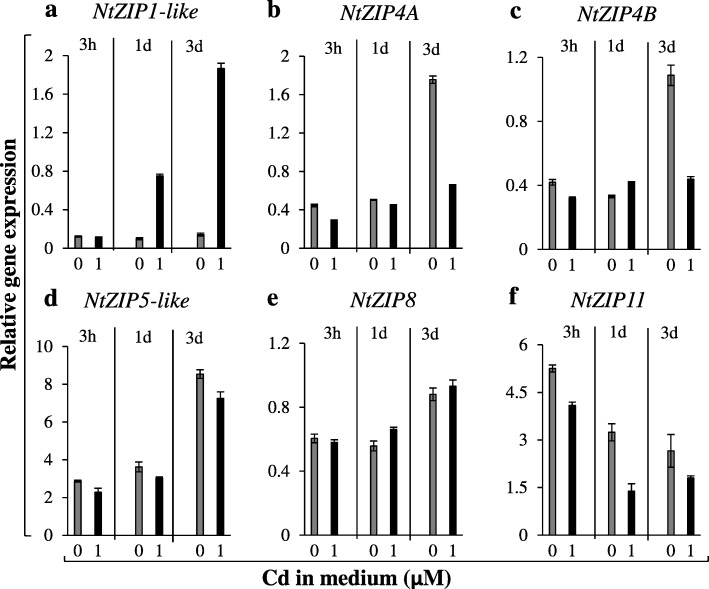
Expression of *ZIP* genes in the leaves of tobacco plants after short-term exposure to Cd. 5.5-week old plants grown in the quarter-strength Knop’s medium were exposed to the control medium containing 1 μM Zn with (1 μM Cd) or without Cd for 3 h, 1 day, or 3 days. Expression of *NtZIP1-like* (**a**), *NtZIP4A* (**b**), *NtZIP4B* (**c**), *NtZIP5-like* (**d**), *NtZIP8* (**e**), *NtZIP11* (**f**). Expression of *NtZIP2*, *NtZIP5A*, *NtZIP5B*, *NtIRT1*, *NtIRT1-like* were not detected in leaves. Gene expression was normalized to the *PP2A* level. Values correspond to arithmetic means ±SD (n = 3); those with the ratio greater than 2 are considered significantly different

**Fig. 7:**
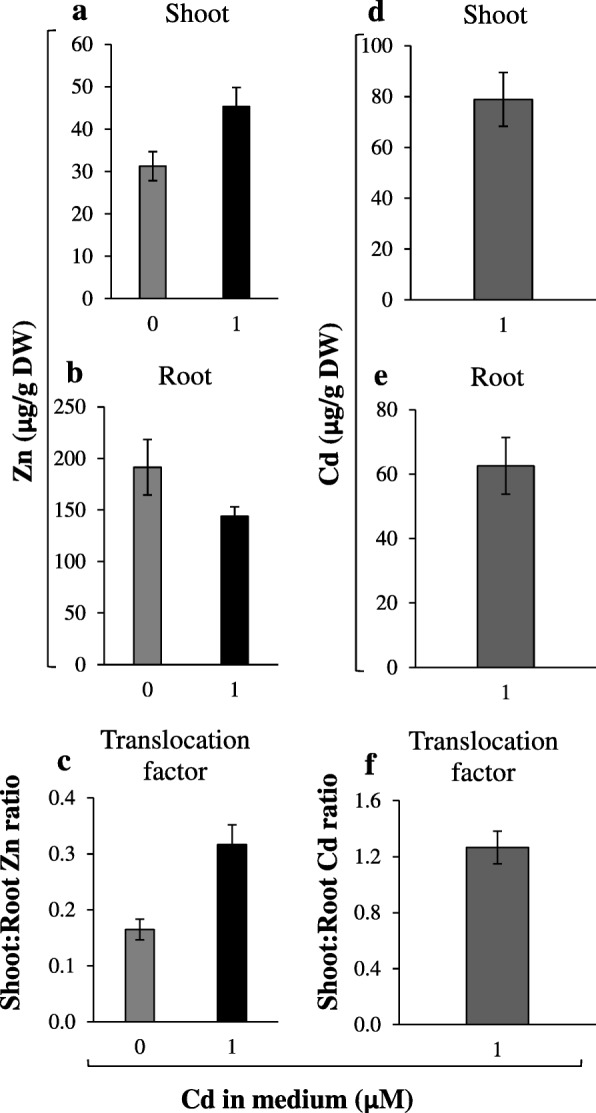
Zinc and cadmium concentration in the shoots and roots of tobacco plants after 3-day exposure to Cd. 5.5-week old plants grown in the quarter-strength Knop’s medium were exposed to the control medium containing 1 μM Zn with (1 μM Cd) or without Cd for 3 days. Zn concentration in the shoots (**a**), and roots (**b**). Cd concentrations in the shoots (**d**) and roots (**e**). The translocation factor (TF) was determined as the ratio of shoot/root concentrations of Zn (**c**), and Cd (**f**). Values correspond to means ±SD (n = 10). Significance of differences between all values were evaluated by Student’s *t*-test (P ≤ 0.05), and it has been determined that each pair of values is significantly different if the SD bars do not overlap. Significance has not been marked above the bars with the letters or arrows to keep the clarity of the graph

The detected increased transcript level of several *ZIP* genes upon 17-day exposure to 1 μM Cd (Fig. 4) suggested induction by Cd. Here, short-term treatment (3 h, 1 day, 3 days) with 1 μM Cd + 1 μM Zn (Figs. 5, 6) indicated Cd-induced increase in the expression of *NtZIP5B* (apical part) and *NtZIP1*-like (all root parts - Fig. 5c, f, and leaves - Fig. 6a). The opposite effect, downregulation of *NtIRT1* and *NtIRT1*-like was found in the middle root part (Fig. 5d, e). To complement the study, the Zn and Cd root/shoot distribution was determined (Fig. 7). It was shown that Zn translocation was already enhanced after 3-day exposure to 1 μM Cd + 1 μM Zn (as compared with medium without Cd).

### Isolation of *NtZIP5B* and bioinformatics analysis

The above experiments indicated that Cd-dependent stimulation of Zn translocation to shoots was accompanied by downregulation of *NtZIP5B* specifically during conditions of zinc deficiency (Fig. 4a3). To learn more about the physiological function of this gene, it was cloned and characterized. Tobacco is an allotetraploid species [30], and numerous genes are present in two copies. Accordingly, here we found two copies of *NtZIP5*: *NtZIP5A* and *NtZIP5B* (Additional file 2). Detailed bioinformatics analysis showed that *NtZIP5A* has a nucleotide sequence identical with *NtZIP1* previously identified by Sano et al. [31], and here we provided data based on which *NtZIP1* was renamed *NtZIP5A*.

**Fig. 8:**
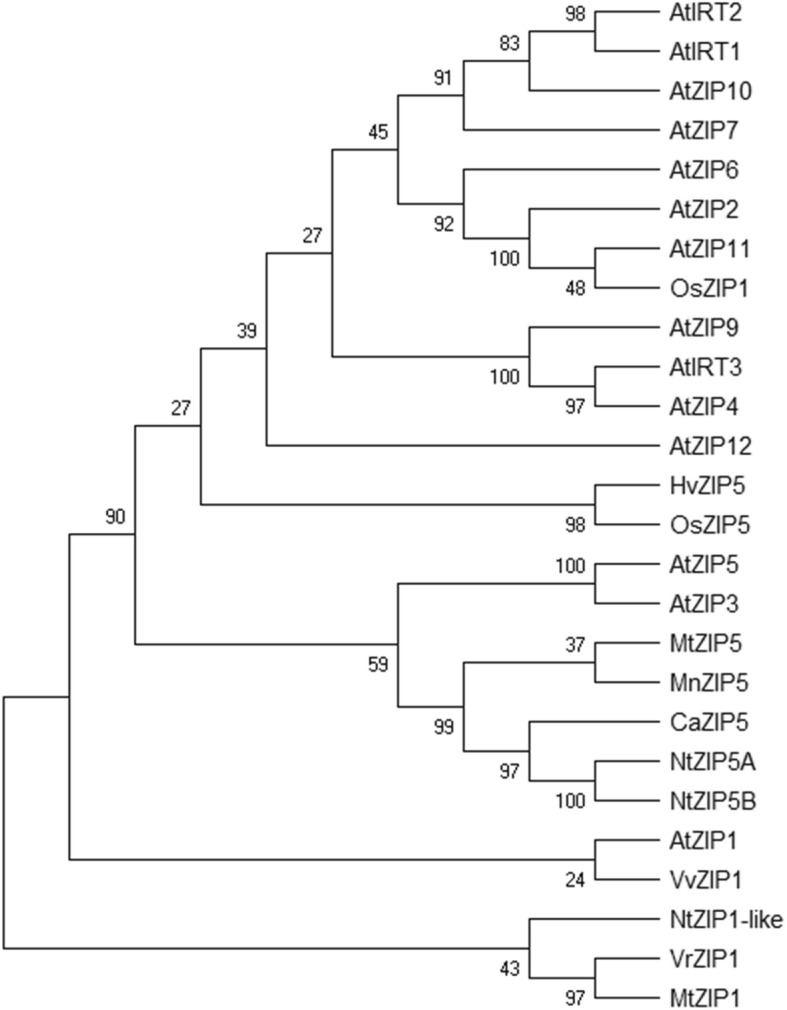
Phylogenetic analysis of ZIP transporters from selected nine species. Unrooted phylogenetic tree for the ZIP proteins was constructed based on amino acid sequences identified in the NCBI database, using MEGA 7.0 software. The length of branches are proportional to the degree of divergence. Numbers in the figure represent bootstrap values (1000 replicates). The accession numbers are as follows: *Nicotiana tabacum* NtZIP5A (NP_001312674.1), NtZIP5B (XP_016449056.1), NtZIP1-like (XM_016652513), *Capsicum annuum* CaZIP5 (PHT75648.1), *Medicago truncatula* MtZIP5 **(**XP_013461166.1), MtZIP1 (XP_013464193.1), *Morus notabilis* MnZIP5 (XP_010093125.1), *Vitis vinifera* VvZIP1 **(**XP_002264603.2), *Arabidopsis thaliana* AtZIP1 (NP_187881.1), AtZIP2 (NP_200760.1), AtZIP3 (NP_180786.1) AtZIP4 (NP_001320672.1), AtZIP5 (NP_172022.1), AtZIP6 (NP_180569.1), AtZIP7 (NP_178488.1), AtZIP9 (NP_001329099.1), AtZIP10 (NP_174411.2), AtZIP11 (NP_564703.1), AtZIP12 (NP_201022.1), AtIRT1 (NP_567590.3), AtIRT2 (NP_193703.2), AtIRT3 (NP_001321605.1), *Vigna radiata* VrZIP1 (XP_014501490.1), *Hordeum vulgare* HvZIP5 **(**ACN93833.1), *Oryza sativa* OsZIP1 **(**XP_015633357.1), OsZIP5 **(**XP_015637510.1)

Our study showed that the sequence of *NtZIP1* (acc. no AB 505626) is identical with the nucleotide sequence NM_001325745.1 described as *Nicotiana tabacum* zinc transporter 5-like (LOC107803903) mRNA and also shares the highest homology (95%) with the predicted *N. tabacum* zinc transporter 5-like (LOC107774100) mRNA (XM_016593570) (Additional file 2). The nucleotide sequences NM_001325745.1 and XM_016593570, and corresponding amino acid (aa) sequences were further used as a query for Blast searches for tobacco genes/proteins with the highest homology. As shown in Additional files 2a and 2b, the identified genes/proteins represented other ZIP5*-*like and ZIP5 from tobacco and other species, which share 100 to 83% identity. These results together with the data from the phylogenetic analysis (Fig. 8; Additional file 3) were the basis for changing the name of sequence A/B 505626.1/NM_001325745.1 from *NtZIP1* to *NtZIP5A*, and sequence XM_016593570.1 was named *NtZIP5B*.

The open reading frame (ORF) of *NtZIP5A*/*NtZIP5B* consists of 1017/1029 bp with 96.46% identity and encodes a predicted protein of 339/343 aa with 98.23% identity. Three exons were identified within the genomic sequences (Additional file 2e; Additional file 3). The phylogenetic relationships are demonstrated on a phylogenetic tree (Fig. 8) and in a Table showing sequence identity at the nucleotide and amino acid level (Additional file 3). It was shown that ZIP5 proteins from different plant species formed two distinct clades. First, both NtZIP5A and NtZIP5B cluster together with CaZIP5, MnZIP5 and MtZIP5 and show 83.63 to 69.62% identity to them. Furthermore, AtZIP5 and AtZIP3 reside on the closest sub-branch. The second ZIP5 clade includes *Monocotyledonous* HvZIP5 and OsZP5, which displayed the lowest identity (56.13 to 54.13%). Similarly, NtZIP5A/B show low identity (54.03 to 62.54%) to ZIP1 proteins (NtZIP1-like, AtZIP1 and VvZIP1), which reside on the closest sub-branch.

**Fig. 9:**
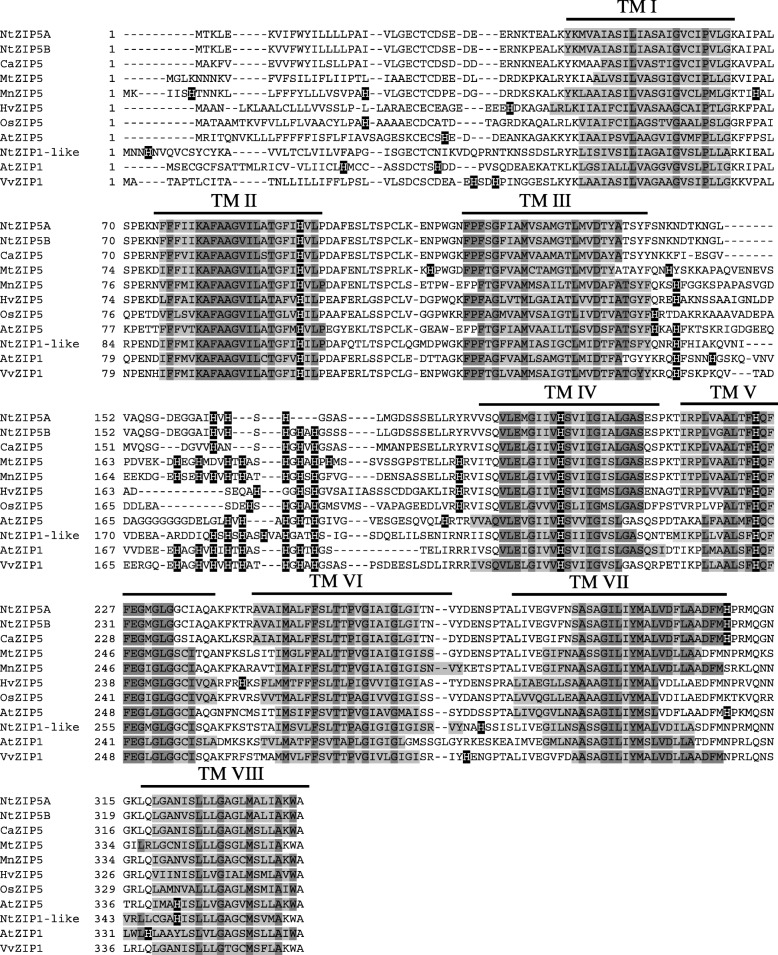
Amino acid alignment of predicted ZIP5 and ZIP1 proteins from different species. Sequences were aligned using Clustal Omega https://www.ebi.ac.uk/Tools/msa/clustalo/. The prediction of membrane-spanning regions was performed using Phobius programme (http://phobius.sbc.su.se/), and indicated as lines above the sequences, and numbered TM I–VIII respectively. Sequences of the transmembrane domains (TM) are marked with light grey; identical amino acids with dark grey; histidine in black. Dashes indicate gaps

The aa sequences of NtZIP5A/B are aligned with ZIP5 and ZIP1 proteins identified in the phylogenetic tree (Figs. 8, 9). In agreement with the structure of ZIP family members from other plants, eight transmembrane domains (TMD), a very short C-terminal tail, and a hydrophilic region between TM domains III/IV were identified. TM III and IV of NtZIP5A/B proteins contain potential metal-binding histidine residues. The length of this region in NtZIP5A/B is 51/55 aa, respectively, which falls within the range of aa numbers typical for plant ZIPs (Additional file 2e). Moreover, amino acids within the TM-IV domain of NtZIP5A/B match the proposed ZIP signature domain [32]. However, within the variable region between the III/IV TMDs, NtZIP5A has three histidine residues, whereas NtZIP5B has five (Fig. 9).

In silico analysis of the promoter regions of *NtZIP5A* and *NtZIP5B* (1797 bp and 1726 bp), identified several *cis*-acting regulatory elements regulated by metals and other abiotic stresses having a high degree of similarity with respect to location and types of elements (Additional file 4). In both copies, in the same location we found ZDRE elements (zinc deficiency-related elements), IDE2 (iron deficiency-responsive element 2), ABRE and ABRE4 (abscisic acid-responsive), MYB*-*like sequence (MYB binding region from *A. thaliana*), Box 4 and GT1 (abiotic stresses such as wounding and pathogen response elements), STRE (Stress Response Element) for heat, osmotic, low pH and nutrient starvation, and CAAT-box (common cis-acting element in promoter and enhancer regions in *Pisum sativum*), whereas light-responsive G-box was detected in a different location [33–37]. Additional copies of Box-4 and STRE were identified in *NtZIP5A*, while IDE2, ABRE, and GT-1 in *NtZIP5B*. Furthermore, four distinct *cis*-acting elements were found exclusively in the promoter of *NtZIP5A*: Box-S and W-box (wounding and pathogen response elements), P-box (giberelin response), and TCT (for light response). Alignments of the promoter regions of *NtZIP5A* and *NtZIP5B* from three different cultivars (K326, Basma Xanthi, and K326) showed identity of sequences (Additional file 4b-d).

Bioinformatics analysis to determine the subcellular localization of NtZIP5B was performed with the use of the ProtComp programme [38, 39]. As shown in Additional file 5, the NtZIP5B protein was predicted to be localized at the plasma membrane.

### Functional analysis of NtZIP5B in yeast

**Fig. 10:**
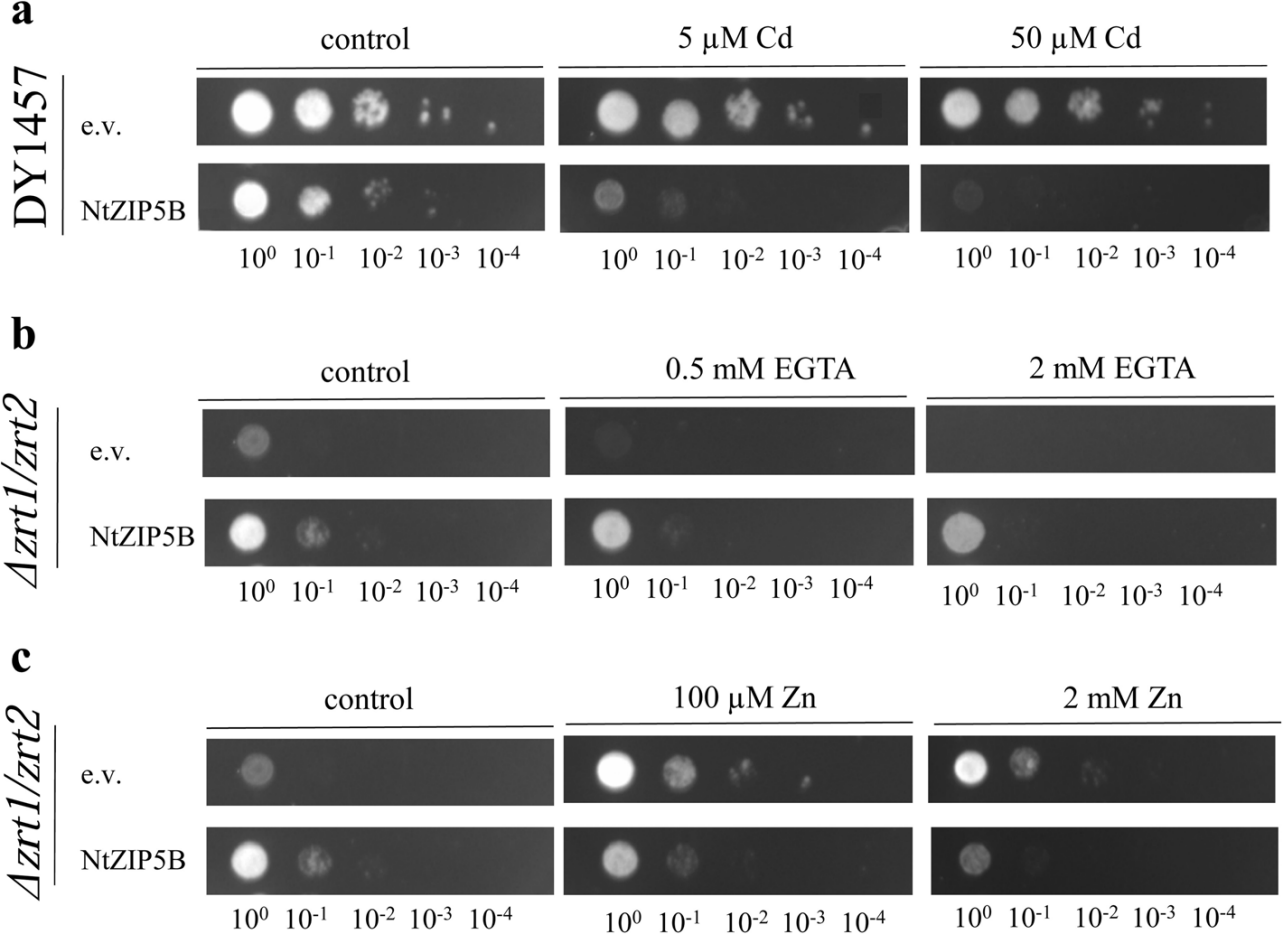
Complementation by *NtZIP5B* cDNA of yeast mutants defective in metal uptake on selective media. Yeast cells: DY1457, *Δzrt1zrt2* (defective in Zn uptake) were transformed with the empty vector pAG426 as a control (e.v.), or with the vector carrying *NtZIP5B* with the stop codon pAG426-ZIP5B (*NtZIP5B*). Yeast cultures were adjusted to an OD600 of 0.2, and 3 μl of serial dilutions (from left to right in each panel) was spotted on SC-URA medium containing 2% (w/v) galactose solidified with 2% agar, supplemented with CdCl_2_ (**a**); EGTA (**b**), or ZnCl_2_ (**c**). The plates were incubated for 3–6 d at 30 °C. The images are representative of three independent experiments taken after 3 days of growth

Increased sensitivity to Cd of the wild-type yeast DY1457 expressing pAG426-*NtZIP5B* as compared with the wild-type expressing pAG426 (Fig. 10a) indicated involvement of NtZIP5B in Cd uptake. Expression of pAG426-*NtZIP5B* in the *zrt1zrt2Δ* mutant line improved growth on minimal medium with increasing concentrations of EGTA (Fig. 10b). Moreover, it led to enhanced sensitivity to high Zn (Fig. 10c). Both results suggest that NtZIP5B mediates Zn uptake.

### Tissue-specific expression of *NtZIP5B*

**Fig. 11:**
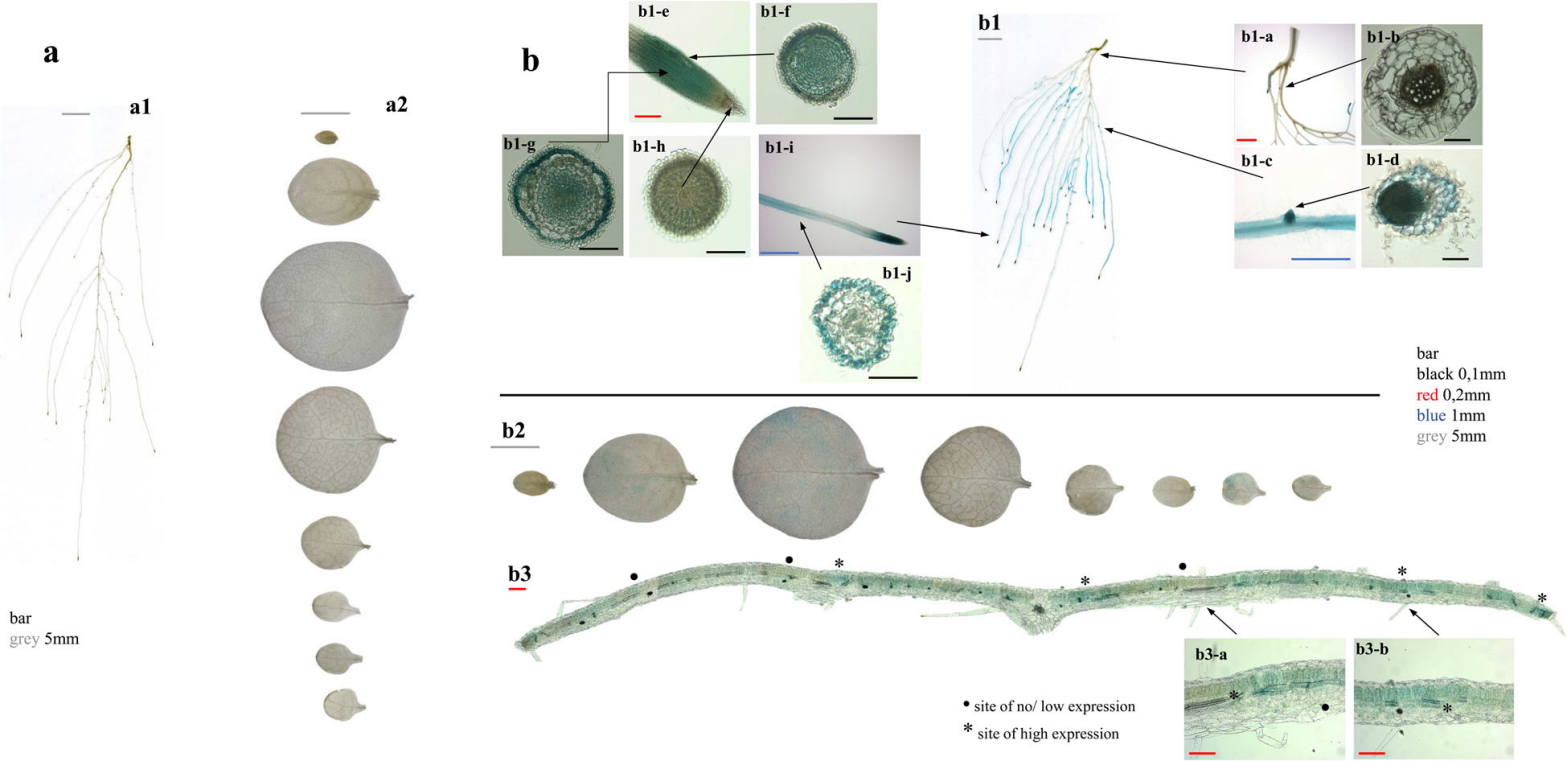
GUS staining pattern of transgenic plants expressing *NtZIP5B*_*prom*_::*GUS*. GUS expression in 4-week-old transgenic and wild-type seedlings grown at control conditions (**a**) and in the Zn-deficient (−Zn) medium (**b**) for 4 days. Whole roots (a1, b1); whole leaves (a2, b2); Basal root part (b1a) and the cross section (b1-b); Root segment from the middle part with the protruding lateral root (b1-c) and the cross section (b1-d); root apex (b1-e) and cross sections at different positions as indicated by arrows (b1-f, b1-g, b1-h); the apical root part (b1-i) and the cross sections from the position indicated by arrow (b1-j); Cross section through the leaf blade (b3) and two chosen areas at higher magnification (b3-a, b3-b). Magnification bars in different colours represent indicated length

Analysis of *NtZIP5Bprom-GUS* transgenic tobacco (seven homozygous T2 lines) showed no expression under control conditions (Fig. 11a1-a2), induction was noted at Zn deficiency (g. 11b1, b2).

GUS activity was not detected in the basal root part (Fig. 11b1-a, b1-b). High levels were found in all tissues of the root tips (except the quiescent center and the root cap) and in newly formed lateral roots (Fig. 11b1-c, b1-d, b1-e, b1-f, b1-g, b1-h). The intensity of staining increased in the epidermis at a distance of approximately 4 mm from the root tip and further up in the more mature region where it was localized primarily in the epidermis (Fig. 11b1-i, b1-j). These results indicate the contribution of NtZIP5B to the uptake of minerals from the medium by epidermal cells, also from the apoplast by cells comprising internal tissues. This function is specific to certain root parts only, primarily to the mature segments of the apical root parts.

Interestingly, in the leaves GUS activity was low and unevenly distributed (Fig. 11b2). Such distribution of *NtZIP5B* expression sites was confirmed by performing histochemical staining directly on sections from fresh leaves (Fig. 11b3). Blue staining was seen in the groups of mesophyll cells (Fig. 11b3-a, b3-b). Thus visually indistinguishable mesophyll cells have differing abilities to take up Zn.

## Discussion

### The apical, middle and basal root parts are distinct constituents of the root with specific contributions to Zn and Cd sequestration and Cd-dependent stimulation of Zn translocation to shoots

Our research showed that in tobacco under both Zn-deficiency and at 1 μM Zn in the medium, Zn translocation to shoots increased in the presence of Cd. This effect was lost at higher Zn concentrations (Fig. 2d). Competitive interactions between Zn and Cd modify the root-to-shoot translocation efficiency of both metals [9, 40–42], however the underlying mechanisms remain unknown. Two basic processes are decisive in the regulation of metal root/shoot partitioning; (i) sequestration in root cells, which determines the availability of the metal for xylem loading, (ii) efficiency of loading into xylem [43–48]. The majority of current research has been conducted on whole roots composed longitudinally of zones that are at different developmental stages (meristematic, differentiation, primary and secondary structures). It is not known if each zone plays distinct role in the regulation of metal root-to-shoot distribution.

In this study, the Zn and Cd accumulation in three root parts was compared among combinations of Zn/Cd concentrations representing contrasting values of the Zn Translocation Factor. Thus, the experimental variant with Cd (where the stimulation of Zn translocation occurred) was compared with the reference combination without Cd (0 μM Zn + 0.25 μM Cd *vs* 0 μM Zn + 0 μM Cd); (1 μM Zn + 0.25 μM Cd *vs* 1 μM Zn + 0 μM Cd); (1 μM Zn + 1 μM Cd *vs* 1 μM Zn + 0 μM Cd). We showed that the capacity of the apical, middle and basal root parts to accumulate Zn and Cd is different and depends on the concentrations of both metals in the soil solution (Fig. 3). The detected Zn/Cd status-dependent root part-specific distribution of Zn and Cd implicates distinct roles of the apical, middle and basal root parts in retention of both metals, consequently in the regulation of their translocation to shoots. Under Zn deficiency, the overall Zn concentration in roots was low (Fig. 2b), thus the distribution pattern characterized by the highest level of the metal in the apical part (Fig. 3a1) likely reflects the necessity of providing Zn to differentiating cells. When the Zn and Cd concentrations increased, the middle and basal parts of the root took over the major role of a sink for excess metal (Fig. 3a2, b2), which might protect the young apical zone from their toxicity. It is not known whether under higher metal status redistribution of metals is involved, or if uptake from the medium directly by the middle part (or even the basal part) is stimulated. The mechanisms remain unknown, since to date, neither the metal accumulation capacity of root parts, nor expression patterns specific for them have been taken into account. Only a few studies have demonstrated the longitudinal variation of metal influx. For example, Laporte et al. [49] showed that the efficiency of the longitudinal translocation of ^109^Cd applied locally was low, and the absorption of ^109^Cd at the tips of first-order sunflower roots was 2.9-fold that of the basal region. The root part-specific retention of Zn and Cd and less effective redistribution of ^109^Cd than ^65^Zn was also reported by Page and Feller [50]. They suggested the contribution of the xylem-to-phloem transfer of ^65^Zn and ^109^Cd taken up by roots in the regulation of the longitudinal distribution of both metals in roots.

Of importance was the discovery that Zn/Cd status-dependent distribution of both metals between the root parts (Figs. 1-3) was accompanied by specific changes in *ZIP*s expression patterns, which fell into four categories (Fig. 4). The first category included *NtZIP2*, *NtZIP5A/B, NtIRT1*, *NtIRT1-like* with the highest expression in the apical part; the second, those with the highest expression in the basal part (*NtZIP1-like* and *NtZIP8*); the third was represented by *NtZIP4A/B* with uniform expression in three root parts; and the fourth, by genes whose expression was not specific for any part of the root (*NtZIP5*-like and *NtZIP11*). These observations expose the complexity of the regulation of metal transfer along the whole root and within its tissues in response to changing metal status, and indicate the existence of signals specific to the apical, middle, and basal root parts that are generated upon a particular combination of Zn and Cd concentrations. Regulation of longitudinal distribution requires numerous genes involved in the uptake of metals, their retention in the particular root parts or effective redistribution, and in their transfer to the shoot.

Seven *ZIPs* were Zn-deficiency inducible, and upregulation was restricted to the apical root part only for *NtIRT1-like*, to the apical and middle part for *NtIRT1,* to the middle and basal part for *NtZIP8*, and to all root parts for *NtZIP1-like, NtZIP5A/B, NtZIP5-like, NtZIP4A/B* (Fig. 4). Furthermore, we detected upregulation by 17-day exposure to 1 μM Cd of *NtIRT1, NtZIP1-like,* and *NtZIP11* in all three root parts, *NtZIP5A/B* and *NtZIP5-like* in apical parts, and *NtZIP8* in apical and middle parts (Fig. 4). Knowing that enhanced transcript levels upon long-term treatment do not necessarily indicate upregulation by Cd itself, but might result from secondary changes in metal homeostasis [51–53], short-term exposure (3 h or 1 day) was applied. Detected upregulation of *NtZIP5B* in the apical root part, and *NtZIP1*-like in all root parts, and downregulation of *NtIRT1* and *NtIRT1-like* in the middle root part (Fig. 5) suggest root-part-specific regulation by Cd. For comparison, Yoshihara et al. [52] showed upregulation of *NtIRT1* in whole tobacco roots, and Barabasz et al. [25], downregulation of *NtZIP4A/B* upon 3-day exposure to 4 μM Cd in leaves, but not in roots.

From the results here described we conclude that the regulation of the longitudinal distribution of Zn and Cd is highly specific, and that the apical, middle and basal root parts play a specific role in accumulation and, likely, in the control of the efficiency of metal translocation to shoots, including the detected stimulation of Zn translocation to shoots in the presence of Cd (Fig. 2). Here, based on distinct expression of tested *ZIP* genes in root parts between combinations of medium containing Cd (at which Zn translocation was enhanced) and the reference medium without Cd, *ZIP* transporters were identified as candidates to be players involved in stimulation of Zn translocation (Fig. 4). However, as shown at the graphic summary (Additional file 6) it turned out that for each combination of Zn/Cd concentrations, different *ZIP* genes with modified expression were identified: (i) *NtZIP5*-like and *NtZIP5A/B* at 0 μM Zn + 0.25 μM Cd; (ii) *NtZIP2* at 1 μM Zn + 0.25 μM Cd, (iii) *NtZIP11, NtZIP8, NtZIP1-like, NtIRT1, NtIRT1-like,* and *NtZIP5-*like at 1 μM Zn + 1 μM Cd. Thus, the same phenomenon (stimulation of Zn translocation in the presence of Cd) detected at distinct combinations of Zn and Cd concentrations (0 μM Zn + 0.25 μM Cd; 1 μM Zn + 0.25 μM Cd; 1 μM Zn + 1 μM Cd) was accompanied by modified expression of different *ZIP* genes in a root part-specific manner (Additional file 6). This indicates that distinct molecular mechanisms or regulatory processes that contribute to Cd-dependent stimulation of Zn translocation to shoots may be involved at different Zn/Cd concentrations in the medium. This is likely linked to different metal statuses in the tested combinations (including exposure to low and severe stress resulting from the presence of Cd) that determine the expression pattern of sets of genes involved in different defense mechanisms [7, 54, 55]. To fully understand the underlying mechanisms, further research is needed to identify all regulatory components of the metal cross-homeostasis network.

### *NtZIP5A/B* –a novel Zn deficiency-inducible transporter involved in Zn and Cd uptake

*NtZIP1* (AB505626 / NM_001325745.1) was first cloned by Sano et al. [31] from tobacco BY-2 cells. However, he focused primarily on *NtNRAMP1*, and only an initial study on *NtZIP1* was performed. In our study, we renamed the gene *NtZIP1* as *NtZIP5A* based on detailed bioinformatics analysis and cloning. First, sequences with the highest homology to NM_001325745.1 (identified in the NCBI data base) all belonged to *ZIP5* genes (Additional file 2c-e). Second, phylogenetic analysis of predicted proteins showed that tobacco NtZIP5A/NtZIP5B formed a distinct clade with dicot CaZIP5, MnZIP5, MtZIP5, AtZIP5 and AtZIP3 (Fig. 8), while two monocot HvZIP5 and OsZIP5 formed a separate branch. These suggest functional divergence between the *Dicotyledonous* and *Monocotyledonous* ZIP5 transporters. Moreover, alignments of predicted amino acids of NtZIP5A/NtZIPB with other ZIP proteins (Fig. 9) also showed high sequence conservation with typical ZIP structures (eight transmembrane domains, a short C-terminal tail, a variable region between TM3-TM4 containing histidine residues) [32, 56, 57].

Furthermore, a yeast growth assay using the *zrt1zrt2* mutant line and wild type expressing *NtZIP5B* suggested that Zn and Cd are substrates (Fig. 10). Together with in silico bioinformatics analysis suggesting localization of NtZIP5B in the plasma membrane (Additional file 5), these results point to this protein’s role in Zn and Cd uptake. For comparison, HvZIP5, OsZIP5, MtZIP5, and ZmZIP5 have also been shown to be Zn uptake proteins, moreover, HvZIP5, OsZIP5 and ZmZIP5 have been localized to the plasma membrane [58–62].

In the tobacco genome, most genes are present in two copies originating from one of the two ancestors, *N. tomentosiformis* or *N. sylvestris* [30]. Here, two *NtZIP5* homologous genes, *NtZIP5A* and *NtZIP5B*, were identified (Additional file 2), which is similar to the presence of *NtMTP1a* and *NtMTP1b*, or *NtHMA4*α and *NtHMA4*β [63, 64]. In our study, the comparable expression patterns between *NtZIP5A* and *NtZIP5B* found in the apical, middle, and basal parts of roots (Fig. 4a1-a5), and absence of expression in leaves (legend to Fig. 6), suggested their similar functions in the regulation of metal homeostasis. Analysis of the *cis*-acting elements within the promoter region of new *NtZIP5A/B* genes showed the same number and localization of ZDRE elements responsible for upregulation under Zn-deficiency [36] and two IDE2s (iron-deficiency-responsive element 2) [33, 35]. Thus, both *NtZIP5A* and *NtZIP5B* likely play a similar role in the regulation of metal homeostasis. Numerous other regulatory sequences also displayed identity, with the exception, however, of four elements that were detected only within the *NtZIP5A* promoter, which are regulators of responses to wounding and pathogens (Box S and W-box), light (TCT motif), and giberellin (P-box) (Additional file 4). Hence compared with *NtZIP5B*, one might speculate that *NtZIP5A* could be involved more broadly in signaling pathways induced by multiple factors*.* Interestingly, the presence of sequences responsible for responses to various abiotic and biotic stresses and to abscisic acid (ABA) points likely not only to the existence of mechanisms by which metal transporters are regulated by numerous factors, but also to the importance of Zn homeostasis in a plant’s response to them. These could be a manifestation of cross-homeostasis not only between different metals, but also between abiotic and biotic stresses.

In addition, as shown by GUS expression analysis, NtZIP5B primarily provides root tissues with Zn under Zn-deficiency conditions (Fig. 11b1). Strong expression detected within the apical meristem across all tissues (Fig. 11b1-e, b1-f, b1-g) and further up in the epidermis (Fig. 11b1-i, b1-j) points to its function in the uptake of minerals from the medium. NtZIP5B is the new ZIP protein for which involvement in the absorption of Zn directly from the soil solution has been indicated. This function was first ascribed to the IRT1 protein [27, 28, 65], also to IRT3 [66], and recently to NtZIP4B, which provided Zn for the whole root up to its basal part, except for the very apical meristem [25]. NtZIP5B provides metals also to palisade parenchyma cells, but interestingly, not across the entire leaf blade (Fig. 11b2, b3), indicating the distinct ability of mesophyll cells to store Zn, as suggested by Siemianowski et al. [67].

## Conclusions

The presented results show that the apical, middle and basal root parts played distinct roles in the sequestration of Zn and Cd that depended on the reciprocal combinations of the concentrations of both metals. The detected regulation of metal distribution within root parts could be regarded as a mechanism protecting young meristematic cells within the root apex. Moreover, it might contribute to the regulation of Zn/Cd root-to-shoot distribution, likely by formation of a metal pool available for translocation to shoots. Next, the identified categories of the root part-specific expression pattern of tobacco *ZIP* genes that depended on the Zn/Cd status provide a first glimpse into the complexity of the regulatory cross-talk between Zn and Cd. This is a key element in determining metal distribution among root parts and the contribution of underlying processes in the regulation of metal root-to-shoot translocation, including the stimulation of Zn translocation in the presence of Cd and associated changes in the expression level of *ZIP* genes. We were the first to show the possible involvement of *ZIP* genes in this process, although to get a complete picture it is necessary not only to fully understand their physiological role, but also to identify other genes acting in concert.

Furthermore, we conclude that the newly cloned tobacco *NtZIP5B* is a Zn-deficiency inducible transporter involved in absorption of Zn and Cd from the soil solution. It performs uptake functions in the root epidermis, but also provides Zn to internal root tissues such as xylem parenchyma, and in leaves to groups of palisade parenchyma cells. The irregular expression of *NtZIP5* within the palisade parenchyma indicates that these cells have a distinct capacity to take up metals. Further study is necessary to reveal the identity of these cells.

## Methods

### Plant material, growth conditions and treatments

The experiments were performed on tobacco (*Nicotiana tabacum* var. Xanthi) plants. Seeds were obtained from the stock of the Institute of Biochemistry and Biophysics PAS, Warszawa, Poland in 2002, since then they were propagated in the greenhouse of the University of Warsaw for our experiments. Plants were cultivated in a controlled environment chamber as previously described [25].

Surface-sterilized seeds in 8% sodium hypochlorite (w/v) were grown for the first three weeks on vertically positioned Petri dishes containing quarter-strength Knop’s medium, 2% sucrose (w/v) and 1% agar (w/v). Afterwards plants were transferred to aeriated hydroponic control medium (quarter-strength Knop’s; composition is given in Barabasz et al. [68]). They were grown in 2 L pots (5 plants per pot) for a period depending on the experiment (details below). The nutrient solution was renewed every 3 days.

Plant samples were collected at the end of each experiment. For expression analysis, plant material was frozen in liquid nitrogen and stored at − 80 °C. The experiments were done in three independent biological replicates. For each experimental variant, plant material was collected and pooled from 10 to 30 plants in total.

For determination of Zn and Cd concentrations, roots were washed in ice-cold 5 mM CaCl_2_ according to Barabasz et al. [68]. Plant samples were dried at 50 °C and stored at room temperature (RT) until analysis.

#### Long-term experiments

Three-week-old seedlings were transferred from the agar-solidified medium to the hydroponic control medium and grown for three days, then exposed for 17 days to the control medium supplemented with different Zn (as ZnSO_4_) and Cd (as CdCl_2_) concentrations.

To determine Zn and Cd root/shoot partitioning, plants were exposed to pairwise combinations of Zn (0; 1; 5; 10; 50 μM) and Cd (0; 0.25; 1; 4 μM) concentrations, then whole roots and shoots were collected.

To determine Zn and Cd concentrations and the expression of tobacco *ZIP* genes in the apical (distal), middle, and basal (proximal) parts of the roots, plants were exposed to Cd (0; 0.25; 1 μM) in the presence of 1 μM Zn or without Zn, then the following root parts were collected: (i) apical (distal) parts (2.5 cm) from the main and lateral roots (containing meristematic tissues, differentiation zone and young primary tissues); (ii) basal (proximal) parts equal to 1/4 of the total length of the main roots (containing primary tissues and within the central cylinder, secondary conductive tissues); (iii) middle parts making up the remaining parts of the main roots (containing primary tissues). The basal and the middle parts of the roots were collected from the main root only (adventitious roots were excised). Taking into consideration the changes of the root branching pattern in plants exposed to a set of Zn and Cd concentrations in the medium, to be able to compare samples collected from plants grown at different medium compositions, the basal and the middle parts of the roots were collected from the main root only.

#### Short-term experiments

Three-week-old seedlings were transferred from the agar-solidified medium to hydroponic control medium for 2.5 weeks, then were exposed to pairwise combinations of Zn (0; 1 μM) and Cd (0; 1 μM) for 3 h, 1 day, or 3 days. Leaves (the 2nd and 3rd counting from the base), and roots (collected as described above) were frozen for expression analysis. Moreover, after 3-day Cd, treatment roots and shoots were collected for determination of Zn and Cd concentrations.

### Transcript analysis

The quantitative real-time PCR (qRT-PCR) based analysis was performed as described by Papierniak et al. [20]. In brief, the relative quantities of each transcript in the samples was calculated based on the comparative ΔCt (threshold cycle) method [69]. As an internal control tobacco *NtPP2A* (*protein phosphatase 2A;* AJ007496) was used, and its stability in the range of applied metal concentrations are given in Additional file 7. Primer sequences are listed in Additional file 8. For each sample, reactions were set up in three repetitions to calculate means. Studies were conducted on a minimum of three biological replicates.

### Determination of metal concentrations

Mineralization and determination of Zn and Cd concentrations by Atomic Absorption Spectrophotometry (AAS) were performed according to Barabasz et al. [53]. To compare the efficiency of Zn and Cd root-to-shoot translocation between plants grown at the applied medium compositions, the Translocation Factor (TF) was determined as the ratio of shoot/root Zn and Cd concentrations [9].

### Cloning of *NtZIP5B* and generation of constructs

In this study, the full length open reading frame (ORF) of *NtZIP5B* was amplified by PCR using Phusion HF polymerase (Thermo Scientific) with the cDNA transcribed from total RNA. The primer sequences are given in Additional file 2c, d, and in Additional file 8. Full length *NtZIP5B* containing the STOP codon was cloned into a vector pENTR™/D-TOPO^R^ (Invitrogen), and the presence of a correct insert in the plasmid pENTR/D-TOPO-*NtZIP5B* was checked by sequencing (Genomed, Poland).

For yeast complementation assays the vector pAG426-*NtZIP5B* was generated by LR recombination between the pENTR/D-TOPO-*NtZIP5B* and the destination vector pAG426GAL-cccB-EGFP.

To determine the tissue-specific expression of *NtZIP5B*, the promoter sequence was cloned with the use of pENTR Directional TOPO Cloning Kits (Invitrogen). The promoter sequence of 1726 bp upstream of the start codon was amplified with the use of primers specific to the genomic sequence AWOK01252628.1, and cloned to the vector pENTR/D-TOPO. The CACC sequence was added to the forward primers (Additional file 8). One Shot TOP10 *E. coli* was transformed with the construct pENTR/D::*ZIP5B*_prom_ and the insert was sequenced. The *NtZIP5B* promoter was cloned in translational fusion with the *uidA* gene (*GUS* gene) in the destination binary vector, pMDC163, by LR recombination and the sequence of the insert in the construct pMDC163::*NtZIP5B*_prom_::GUS was checked again.

### NtZIP5 sequence analysis

The nucleotide cDNA sequences of *NtZIP5A* and *NtZIP5B* were translated to protein sequences with the use of the ExPASy translate tool (http://web.expasy.org/translate/). Similarity searches were performed using the BLAST algorithm. Multiple sequence alignment of the NtZIP5A/B and ZIP proteins from chosen plant species was performed with CLustalW [70]. The phylogenetic tree was constructed with MEGA 7.0 software [71] using the maximum likelihood method with 1000 bootstrap replicates. Potential transmembrane domains were identified with Phobius software [72]. To identify *Nicotiana tabacum ZIP* sequences in the NCBI database, BLASTn (Basic Local Alignment Search Tool) searches were performed based on homology to already known *A. thaliana* sequences. The subcellular localizations of NtZIP4B protein was predicted with the use of the ProtComp v. 9.0 online; http://www.softberry.com/berry.phtml?topic=protcomppl&group=programs&subgroup=proloc.

### Yeast growth assay

Two *Saccharomyces cerevisiae* strains were used in the experiments: (i) wild-type DY1457 (MATa, ade1 can1 his3 leu2 trp1 ura3), (ii) mutant ZHY3 –*zrt1zrt2* (DY1457 + zrt1::LEU2, zrt2::HIS3) defective in high- and low-affinity zinc uptake. The prepared construct pAG426-*NtZIP5B* and the empty vector pAG426GAL were used for yeast transformation using the lithium acetate protocol [73].

The *zrt1zrt2Δ* strain defective in high- and low-affinity Zn uptake was used to determine if NtZIP5B mediates Zn uptake. Yeast were grown as described by Barabasz et al. [25]. Briefly, the wild type (WT) DY1457 was transformed with the empty vector pAG426GAL, and the mutant *zrt1zrt2* with pAG426-*NtZIP5B.* Next, they were cultivated on the SC-URA medium (containing 0.2 mM Zn) with galactose. Liquid cultures were spotted onto agar-solidified plates containing SC-URA with GAL supplemented with 0.5 to 2 mM EGTA (ethylene glycol-bis(β-aminoethyl ether)-N,N,N′,N′-tetraacetic acid) or with 0.1 to 2 mM Zn concentrations.

To check whether NtZIP5B transports Cd, the WT strain DY1457 was transformed with the empty vector pAG426GAL, and with the vector pAG426-*NtZIP5B*. The growth of yeast cultivated on an agar-solidified SC-URA with GAL medium containing 5 to 50 μM Cd (CdCl_2_) was monitored.

### Tissue-specific expression of *NtZIP5B*_*prom*_::*GUS*

The pMDC163::*NtZIP5B*_prom_::*GUS* construct was used for tobacco leaf disc transformation using an *Agrobacterium*-mediated method as previously described [68]. Transformants were selected for hygromycin resistance. The T2 homozygous lines were selected from T1 plant lines with a segregation ratio of 3:1 (tolerant:sensitive).

Three-week-old transformants (seven lines) and the wild type were transferred from the agar-solidified medium to hydroponic control medium for 3 days, then exposed to the medium without Zn (and in parallel to the control one) for 4 days. Whole seedlings were used for GUS histochemical staining. Representative individuals from representative lines were photographed. To visualize the expression of *NtZIP5B* at the tissue/cell level, stained root fragments were embedded in 3% agarose, 150 μ sections were cut on a Vibratome (Leica VT1000S, Heidelberg, Germany) and used for microscopic analysis (OPTA-TECH microscope). In addition, leaf cross-sections made from fresh unstained leaves on a Vibratome were subjected to histochemical staining. GUS activity was determined as described by Barabasz et al. [25].

### Statistical analysis

Evaluation of statistical significance was performed at the 0.05 probability level using Student’s *t*-test. Experiments were performed with at least three independent replicates.

## Supplementary information

**Additional file 1.** Dry weight of plant samples used to measure Zn and Cd concentrations

**Additional file 2 **Nucleotide and amino acid sequences of *NtZIP5A/B* and other selected *ZIP5* from other plant species

**Additional file 3 **Sequence identity between the *NtZIP5A, NtZIP5B* and chosen *ZIP5* and *ZIP1* nucleotide sequences (a) and predicted proteins (b) from selected species

**Additional file 4. **Supplementary information on cloning of *NtZIP5B* promoter (primers, promoter sequences)

**Additional file 5.** Bioinformatics analysis of NtZIP5B subcellular localization

**Additional file 6. **Graphical presentation of root part-specific changes in *ZIP*s expression

**Additional file 7. **Stability of *PP2A*

**Additional file 8. **Primer sequences used for expression analysis, cloning of *NtZIP5B* and promoter isolation

## Data Availability

Datasets supporting conclusions of this article are included within the article and its additional files.

## Abbreviations

Aa: Amino acid
AAS: Atomic Absorption Spectrophotometry
ABA: Abscisic acid
ABRE: Abscisic acid-responsive element
BLAST: Basic Local Alignment Search Tool
Cd: Cadmium
Cu: Cooper
EGTA: Ethylene glycol-bis(β-aminoethyl ether)-N,N,N′,N′-tetraacetic acid
GAL: Galactose
GUS: β-glucuronidase
IDE: Iron deficiency-responsive element
IRT: Iron regulated transporter
Mn: Manganese
MYB-like: Myeloblastosis-like
ORF: Open reading frame
RT: Room temperature
RT-qPCR: Reverse transcription quantitative polymerase chain reaction
SSH: Suppression Subtractive Hybridization
STRE: Stress Response Element
TMD: Transmembrane domain
WT: Wild type
ZDRE: Zinc deficiency-related element
ZIP: ZRT/IRT related Proteins
Zn: Zinc
ZRT: Zinc related transporter
