# Supplementary material for: Zn/Cd status-dependent accumulation of Zn and Cd in root parts in tobacco is accompanied by specific expression of ZIP genes
Source: BMC Plant Biol. 2020 Jan 22;20:37. doi: 10.1186/s12870-020-2255-3 (PMC6977228; doi:10.1186/s12870-020-2255-3)
Supplement: Supplementary file 1 — Additional file 1. Dry weight of plant samples used to measure Zn and Cd concentrations [file 12870_2020_2255_MOESM1_ESM.pdf]

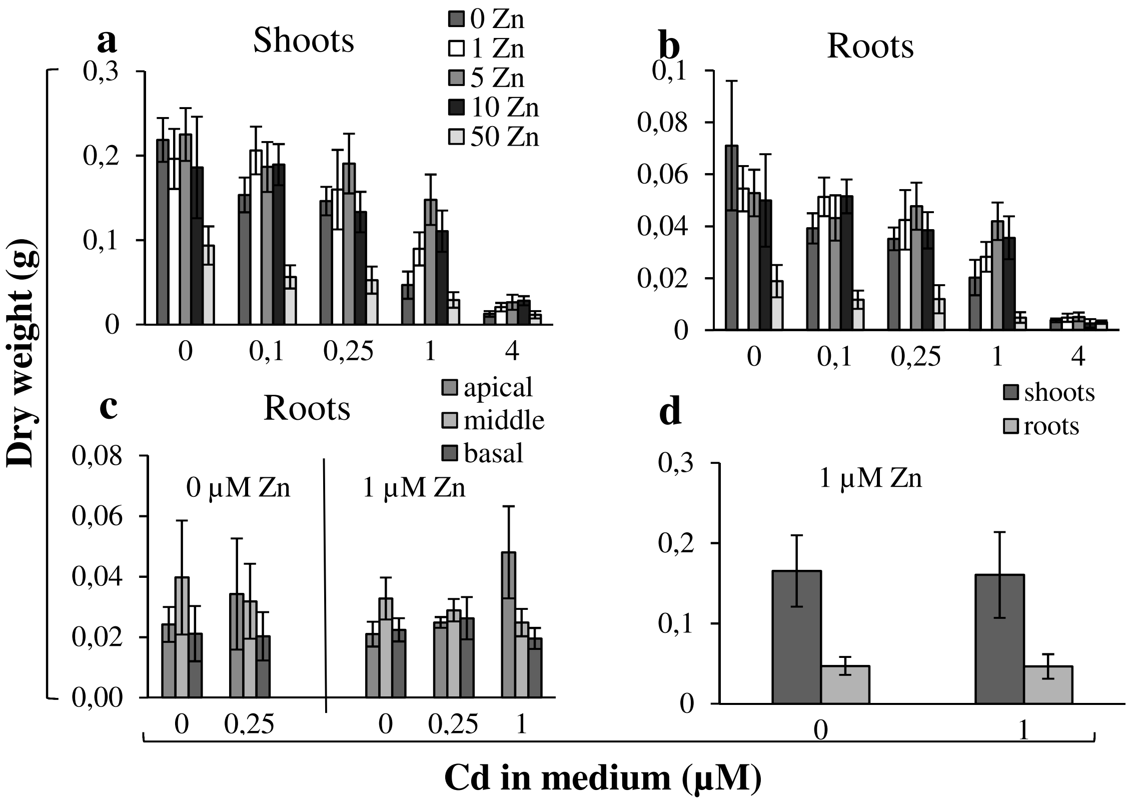

Additional file 1:

Dry weight of shoots (a) and roots (b) of 6-week-old plants grown for 3.5 weeks at control conditions and subsequently exposed to paiwise combinations of Zn (0; 1; 5; 10; 50 μM) and Cd (0; 0.1; 0.25; 1; 4 μM) concentration for 17 days, for which Zn and Cd contentrations were determined and shown in Figs. 1,2

Dry weight of the apical, middle and basal root parts (c) of 6-week-old plants grown for 3.5 weeks at control conditions and subsequently exposed to combinations of Zn (0; 1 μM) and Cd (0; 0.25 μM; 1 μM) concentration for 17 days, for which Zn and Cd contentrations were determined and shown in Fig. 3.

Dry weight of the shoots and roots (d) of 5 week-old plants grown at control conditions and subsequently exposed to combinations of Zn (1 μM) and Cd (0; 1 μM) concentrations for 3 days, for which Zn and Cd contentrations were determined and shown in Fig. 7.
