# Supplementary material for: Zn/Cd status-dependent accumulation of Zn and Cd in root parts in tobacco is accompanied by specific expression of ZIP genes
Source: BMC Plant Biol. 2020 Jan 22;20:37. doi: 10.1186/s12870-020-2255-3 (PMC6977228; doi:10.1186/s12870-020-2255-3)
Supplement: Supplementary file 2 — Additional file 2 Nucleotide and amino acid sequences of NtZIP5A/B and other selected ZIP5 from other plant species [file 12870_2020_2255_MOESM2_ESM.pdf]

Additional file 2:

Content:

(a) Identification of *ZIP5* genes based on a search through NCBI data base p.1

(b) Comparison of amino acid sequence of NtZIP5A / B with ZIP5 sequences identified in the NCBI data base; NtZIP5A / B sequences were used as a query. p12

(c) Comparison of cDNA nucleotide sequences of *NtZIP5A* and *NtZIP5B*. p.15

(d) Specificity of primers used for expression analysis of *NtZIP5A* and *NtZIP5B*. p.17

(e) Information on nucleotide and amino acid sequences of *NtZIP5A* and *NtZIP5B*, and chosen *NtZIP5* and *NtZIP1* genes from selected plant species. p.18

(a) Identification of *ZIP5* genes based on a search through NCBI data base; as a query the following sequences were used:

(a1) AB 505626.1 (Sano et al., 2012) which is identical with the sequence **Acc no NM_001325745.1** (named later in this study as ***NtZIP5A***);
(a2) Acc no XM_016593570.1 (named later in this study as ***NtZIP5B***).

(a1) The sequence Acc no NM_001325745.1 (named later in this study as *NtZIP5A*) was used as a query to search through NCBI data base for tobacco genes with the highest homology.

The list of tobacco genes identified as the best Blast Hits are given in the Table below,

Description	Max score	Total score	Query cover	E value	Ident	Accession no.
Nicotiana tabacum NtZIP1 mRNA for metal transporter (Sano et al., 2012, in this paper renamed to *NtZIP5A*)	1884	1884	100%	0.0	100%	AB505626.1

PREDICTED: *Nicotiana sylvestris* zinc transporter 5-like (LOC104220059), mRNA	1635	1635	100%	0.0	95%	XM_009770855.1
**PREDICTED: *Nicotiana tabacum* zinc transporter 5-like (LOC107774100), mRNA [cloned in this study and named NtZIP5B]**	**1629**	**1629**	**100%**	**0.0**	**95%**	**XM_016593570.1**
PREDICTED: *Nicotiana attenuata* zinc transporter 5-like (LOC109217305), mRNA	1563	1563	100%	0.0	94%	XM_019381545.1
PREDICTED: *Nicotiana tomentosiformis* zinc transporter 5 (LOC104104056), mRNA	1369	1369	100%	0.0	91%	XM_009612040.2
PREDICTED: *Nicotiana sylvestris* zinc transporter 5-like (LOC104225083), mRNA	1301	1301	99%	0.0	90%	XM_009776848.1
PREDICTED: *Solanum tuberosum z*inc transporter 5-like (LOC102599833), mRNA	1147	1147	100%	0.0	87%	XM_006353660.2
PREDICTED: *Solanum pennellii* zinc transporter 5-like (LOC107024498), mRNA	1131	1131	100%	0.0	87%	XM_015225484.1
PREDICTED: *Solanum tuberosum* zinc transporter 5-like (LOC102600170), mRNA	1120	1120	100%	0.0	86%	XM_006353661.2

Alignments of ORF+STOP nucleotide sequences between the query sequence (**Acc no NM_001325745.1,** named later as ***NtZIP5A*****)** and sequences of genes identified in the NCBI data base as the best hits. Sequences were aligned using Clustal Omega https://www.ebi.ac.uk/Tools/msa/clustalo/. The prediction of membrane regions was performed using Phobius programme (http://phobius.sbc.su.se/), and indicated as lines above the sequences, and numbered TM I–VIII respectively. Nucleotide sequences coding the transmembrane domains (TM) are marked with light grey; identical nucleotide with dark grey. Dashes indicate gaps.

```
NM_001325745.1-NtZIP5A        ATGACAAAGTTAGAAAAAGTTATTTTTTGGTACATTCTCTTGCTTCTTCCTGCTATAGTA    60
AB505626.1-NtZIP1             ATGACAAAGTTAGAAAAAGTTATTTTTTGGTACATTCTCTTGCTTCTTCCTGCTATAGTA    60
XM_009770855.1-NsZIP5-like    ATGACAAAGTTAGAAAAAGTAGTTTTTTGGTACATTCTCTTGCTTCTTCCTGCTATAGTA    60
XM_016593570.1-NtZIP5B        ATGACAAAGTTAGAAAAAGTAGTTTTTTGGTACATTCTCTTGCTTCTTCCTGCTATAGTA    60
XM_019381545.1-NaZIP5-like    ATGACAAAGTTGGAAAAAGTTGTATTTTGGTACATTCTCTTGCTTCTTCCTGCTATAGTA    60
XM_009612040.2-NtomZIP5       ATGGCAAAGTTAGAAAAAGTTGTTTTTTGGTACATTCTCGTACTTCTTCCTGCTATAGTA    60
XM_009776848.1-NsZIP5-like    ATGGAAAAGTTAAAAAAATGTTATTTTCTGGTACATTCTCGTTCTTTTACCTGCTATAGTA    60
XM_006353660.2-StZIP5-like    ATGACAAAGTTTGAAAAAATGATATTCTGGTACATTCTATTGCTTTTTCCGGCAATAGTA    60
XM_015225484.1-SpZIP5-like    ATGGCACAGTTTAAAAAAACGATATTCTGGTACATTCTATTGATTCTTCCCGCCATAGTA    60
XM_006353661.2-StZIP5-like    ATGGCAAAGATGGAAAAAATTGTATTTTGGTACATTCTATTGCTTCTTCCCGCAATAGTA    60
                              ***  * ** *  ****     * ** ***********  *  ** * ** ** ******

NM_001325745.1-NtZIP5A        TTAGGCGAATGTACTTGTGATTCTGAGGATGAAGAAAGAAACAAAACTGAAGCACTGAAG    120
AB505626.1-NtZIP1             TTAGGCGAATGTACTTGTGATTCTGAGGATGAAGAAAGAAACAAAACTGAAGCACTGAAG    120
XM_009770855.1-NsZIP5-like    TTAGGAGAATGTACTTGTGATTCTGAGGATGAAGAAAGAAACAAACCTGAAGCACTGAAA    120
XM_016593570.1-NtZIP5B        TTAGGAGAATGTACTTGTGATTCTGAGGATGAAGAAAGAAACAAACCTGAAGCACTGAAA    120
XM_019381545.1-NaZIP5-like    TTAGGAGAATGTACATGTGAATCTGAGGATGAAGAAAGAAACAAAACTGAAGCACTGAAA    120
XM_009612040.2-NtomZIP5       TTAGGAGAATGTACTTGTGATTCTGATGAAGAAGAAAGAAACAAAACTGAAGCATTAAAA    120
XM_009776848.1-NsZIP5-like    TTAGGAGAATGTACATGTGATTCTGAGGATGAAGAAAGAAACAAAGCTGAAGCACTGAAG    120
XM_006353660.2-StZIP5-like    TTAAGCGAGTGTACTTGTGATCCTGAGGATGAAGACAGAAACAAAAAAGAAGCACTCAAG    120
XM_015225484.1-SpZIP5-like    TTAGGCGATTGTACTTGTGATCCTGAGGATGAAGACAGAAACAAAAAAGAAGCACTCAAG    120
XM_006353661.2-StZIP5-like    TTAGGCGAATGTACTTGTGATTCGGAGGATGAAGAAAGAAACAAAAGTGAGGCACTCAAG    120
                              *** * ** ***** *****  * ** ** ***** *********   ** *** * **
```

TM I

```
NM_001325745.1-NtZIP5A        TATAAAATGGTAGCAATCGCTTCCATTCTGATTGCCAGTGCAATTGGGGTTTGTATTCCT    180
AB505626.1-NtZIP1             TATAAAATGGTAGCAATCGCTTCCATTCTGATTGCCAGTGCAATTGGGGTTTGTATTCCT    180
XM_009770855.1-NsZIP5-like    TATAAAATGGTAGCAATCGCTTCCATTTTGATTGCCAGTGCAATTGGGGTTTGTATTCCT    180
XM_016593570.1-NtZIP5B        TATAAAATGGTAGCAATCGCTTCCATTTTGATTGCCAGTGCAATTGGGGTTTGTATTCCT    180
XM_019381545.1-NaZIP5-like    TATAAAATGGTAGCAATCGCTACCATTTTGGTAGCCAGTGCAATTGGGGTTTGTATTCCT    180
XM_009612040.2-NtomZIP5       TATAAAATGGTAGCAGTCGCTTCCATTTTAGTTGCCAGTGCATTAGGGGTTTGTATTCCT    180
XM_009776848.1-NsZIP5-like    TATAAAATGGTAGCAGTTGCTTCCATTTTAGTAGCTAGTGCATTAGGGGTTTGTATTCCT    180
XM_006353660.2-StZIP5-like    TACAAAATGGCAGCACTCGCTTCCATTTTGGTAGCGAGTGCTATTGGGGTGTGTATTCCT    180
XM_015225484.1-SpZIP5-like    TACAAAATGGCAGCACTGGCTTCCATTTTGGTAGCGAGTTCCATTGGGGTGATTATTCCT    180
XM_006353661.2-StZIP5-like    TACAAAATGGCAGCACTCGCTTCCATTTTGGTAGCGAGTGCAATTGGGGTGTGTATTCCT    180
                              ** ******* **** * *** ***** *  * ** *** *  * *****   *******

NM_001325745.1-NtZIP5A        GTACTTGGAAAAGCAATTCCAGCTTTAAGCCCAGAGAAGAATTTCTTCTTCATAATCAAA    240
AB505626.1-NtZIP1             GTACTTGGAAAAGCAATTCCAGCTTTAAGCCCAGAGAAGAATTTCTTCTTCATAATCAAA    240
XM_009770855.1-NsZIP5-like    GTACTAGGAAAAGCAATTCCAGCTTTGAGCCCAGAGAAGAATTTCTTCTTCATAATCAAA    240
XM_016593570.1-NtZIP5B        GTACTAGGAAAAGCAATTCCAGCTTTGAGCCCAGAGAAGAATTTCTTCTTCATAATCAAA    240
XM_019381545.1-NaZIP5-like    GTACTAGGAAAAGCAATTCCAGCTTTGAGCCCAGAGAAGAATTTCTTCTTCATAATCAAA    240
XM_009612040.2-NtomZIP5       GTACTTGGAAAAGCAATTCCAGCTTTGAGCCCAGAGAAGAATTTATTTTTTATAATCAAA    240
XM_009776848.1-NsZIP5-like    GTACTAGGAAAAGCAATTCCAGCTTTGAGTCCAGAGAAGAATTTATTTTTCATAATCAAA    240
XM_006353660.2-StZIP5-like    GTTCTGGGCAAGGCGATTCCCGCCTTAAGCCCAGAAAGGAATTTCTTCTTTATAATTAAA    240
XM_015225484.1-SpZIP5-like    GTTCTGGGCAAGGCGATTCCCGCTTTAAGCCCAGAGAGGAATTTGTTCTTTATAGTTAAA    240
XM_006353661.2-StZIP5-like    GTTCTGGGCAAGGTGATTCCCGCCTTAAGCCCAGAGAGGAATTTCTTCTTTATAATTAAA    240
                              ** ** ** ** *  ***** ** ** ** ***** * ****** ** ** *** * ***
```

TM II

```
NM_001325745.1-NtZIP5A        GCTTTTGCTGCTGGTGTGATCCTCGCGACAGGGTTTATACATGTACTTCCTGATGCTTTT    300
AB505626.1-NtZIP1             GCTTTTGCTGCTGGTGTGATCCTCGCGACAGGGTTTATACATGTACTTCCTGATGCTTTT    300
XM_009770855.1-NsZIP5-like    GCTTTTGCTGCTGGTGTGATCCTCGCGACAGGGTTTATACATGTACTCCCTGATGCATTT    300
XM_016593570.1-NtZIP5B        GCTTTTGCTGCTGGTGTGATCCTCGCGACAGGGTTTATACATGTACTCCCTGATGCATTT    300
XM_019381545.1-NaZIP5-like    GCGTTCGCGGCGGGTGTGATCCTCGCGACAGGGTTTATACATGTACTCCCCGATGCATAT    300
XM_009612040.2-NtomZIP5       GCTTTCGCTGCTGGTGTGATCCTCGCGACAGGGTTTATACATGTATTACCTGATGCATAT    300
XM_009776848.1-NsZIP5-like    GCGTTCGCGGCGGGTGTGATCCTCTCGACAGGGTTTATACATGTATTACCTGATGCATAT    300
XM_006353660.2-StZIP5-like    GCTTTTGCGGCTGGTGTGATCTTATCAACGGGGTTTATACATGTACTTCCTGATGCATTT    300
XM_015225484.1-SpZIP5-like    GCTTTTGCGGCTGGTGTGATTTTATCAACGGGGTTTATACATGTGCTTCCTGATGCATAT    300
XM_006353661.2-StZIP5-like    GCTTTCGCGGCTGGTGTTATCCTATCAACGGGGTTTATACATGTACTTCCTGATGCATTT    300
                              ** ** ** ** ***** **  *  * ** **************  * ** ***** * *
```

```
NM_001325745.1-NtZIP5A          GAAAGCTTAACATCGCCATGTTTGAAAGAGAATCCGTGGGGAAATTTTCCTTTTAGTGGA     360
AB505626.1-NtZIP1               GAAAGCTTAACATCGCCATGTTTGAAAGAGAATCCGTGGGGAAATTTTCCTTTTAGTGGA     360
XM_009770855.1-NsZIP5-like      GAAAGCTTAACATCGCCATGTTTGAAAGAGAATCCGTGGGGAAATTTTCCATTTAGTGGA     360
XM_016593570.1-NtZIP5B          GAAAGCTTAACATCGCCATGTTTGAAAGAGAATCCGTGGGGAAATTTTCCATTCAGTGGA     360
XM_019381545.1-NaZIP5-like      GAAAGCTTGACATCGCCATGTTTGAAAGAAAATCCATGGGGAAATTTTCCTTTTAGTGGA     360
XM_009612040.2-NtomZIP5         AAAAGTTTAACATCGCCATATTTGAAAGAAAATCCGTGGGGAGATTTTCCTTTTAGTGGA     360
XM_009776848.1-NsZIP5-like      GAAAGTTTAACGTCGCCATGTTTGAAAGAAAATCCGTGGGGAGATTTTCCATTCAGTGGA     360
XM_006353660.2-StZIP5-like      GAAAGCTTGACTTCGCCGTGTTTGGCAGAACATCCGTGGGGAGATTTCCCTTTTAGTGGA     360
XM_015225484.1-SpZIP5-like      GAAAGTTTGACATCGCCGTGTTTGGCAGAACATCCATGGGGAGATTTCCCTTTTAGTGGA     360
XM_006353661.2-StZIP5-like      GAAAGTTTGACATCTCCGTGTTTGAAAGAGAATCCATGGGGAAATTTCCCCTTTTGTGGA     360
                                 **** ** ** ** ** * ****  ***  **** ****** **** ** **  *****

                                                 TM III
NM_001325745.1-NtZIP5A          TTTATTGCAATGGTTTCTGCAATGGGAACTCTTATGGTGGATACTTATGCAACTTCATAT     420
AB505626.1-NtZIP1               TTTATTGCAATGGTTTCTGCAATGGGAACTCTTATGGTGGATACTTATGCAACTTCATAT     420
XM_009770855.1-NsZIP5-like      TTTATCGCAATGGTTTCTGCAATGGGAACTCTAATGGTGGACACTTATGCAACTTCATAT     420
XM_016593570.1-NtZIP5B          TTTATCGCAATGGTTTCTGCAATGGGAACTCTAATGGTGGACACTTATGCAACTTCATAT     420
XM_019381545.1-NaZIP5-like      TTTATTGCAATGGTTTCTGCTATGGGAACTCTTATGGTGGATACTTATGCAACTTCATAT     420
XM_009612040.2-NtomZIP5         TTTATTGCAATGGTTTCTGCGATGGCGACTCTGATGGTGGATACTTATGCAACTTCATAT     420
XM_009776848.1-NsZIP5-like      TTTATTGCAATGGTTTCTGCAATGGCGACTCTGATGGTGGATACTTATGCAACTTCGTAT     420
XM_006353660.2-StZIP5-like      TTTATTGCAATGGTTTCAGCACTGGCGACTCTGATGGTGGATACTTATGCAACTTCGTAT     420
XM_015225484.1-SpZIP5-like      TTTATTGCAATGGTTTCTGCATTGGCGACTCTGATGGTGGATACTTATGCAAATTCGTAT     420
XM_006353661.2-StZIP5-like      TTTGTTGCTATGGTTTCTGCAATGGCGACTCTGATGGTGGATACTTATGCAACATCTTAT     420
                                *** * ** ******** **  ***  ***** ******** **********  ** ***

NM_001325745.1-NtZIP5A          TTCAGTAACAAAAATGACACGAAAAATGGATTGGTGG---CTCAGTCTGGAGATGAAGGA     477
AB505626.1-NtZIP1               TTCAGTAACAAAAATGACACGAAAAATGGATTGGTGG---CTCAGTCTGGAGATGAAGGA     477
XM_009770855.1-NsZIP5-like      TTCAGTAACAAAAATGATACGAAAAATGGATTGGTGG---CTCAGTCTGGAGATGAAGGA     477
XM_016593570.1-NtZIP5B          TTCAGTAACAAAAATGATACGAAAAATGGATTGGTGG---CTCAGTCTGGAGATGAAGGA     477
XM_019381545.1-NaZIP5-like      TTCAGAAACAAAAATGACACGAAAAATGGATTGGTGG---CTCAGTCTGGAGATGAAGGA     477
XM_009612040.2-NtomZIP5         TATAAGAATAAATC---TGCGAAAAATGGTGTGGTGG---CTCAGTCTGGAGATGAAGAA     474
XM_009776848.1-NsZIP5-like      TATAAGAATAAATC---TGTGAAAAATGGTGTGGTGG---CTCAGTCTGGAGATGAAGAA     474
XM_006353660.2-StZIP5-like      TACAGTAAGAAAAATT---TGAAAAATGGAGTGGTGGCGACTGAGGCTGGATATGAACAA     477
XM_015225484.1-SpZIP5-like      TACAGTAAGAAAAATT---TGAAAAATGGAGTGGTGGTGGCTCAGTCTGGAGATGAAGGA     477
XM_006353661.2-StZIP5-like      TACAATAAGAAGAATT---TGAAAAATGGAGTGGTGACGACTGAGGGTGGAGATGAAGAA     477
                                *  *  ** **         *********  *****    ** **  **** *****  *

NM_001325745.1-NtZIP5A          GGAGCTATTCATGTTCATTCACA------------TGGTTCAGCATCACTGA-TGGGTGA     524
AB505626.1-NtZIP1               GGAGCTATTCATGTTCATTCACA------------TGGTTCAGCATCACTGA-TGGGTGA     524
XM_009770855.1-NsZIP5-like      GGAGCTATACATGTTCATTCACATGGCCATGCACATGGTTCATCATCACTGC-TGGGTGA     536
XM_016593570.1-NtZIP5B          GGAGCTATACATGTTCATTCACATGGCCATGCACATGGTTCATCATCACTGC-TGGGTGA     536
XM_019381545.1-NaZIP5-like      GGAGCTATTCATGTTCATTCACATGGCCATGCACATGGTGCATCATCAATGA-TGGGTGA     536
XM_009612040.2-NtomZIP5         TTAGTT---AATGTTCATTCACATGGCCATGTACATGGCTCAACATCAATGA-TTGGTGA     530
XM_009776848.1-NsZIP5-like      GGAGTTATTCATGTTCATTCACATGGCCATGCACATGGTTCAACATCAATGA-TTGGTGA     533
XM_006353660.2-StZIP5-like      GGAGTTGTTCATGCTCATTCTCATG------------GC-CACGTTCATGGTTTGTCTGA     524
XM_015225484.1-SpZIP5-like      GGAGTGGTTCATCCTCACTCTCATG------------GTTCAGGATCAATGA-TGGTTGA     524
XM_006353661.2-StZIP5-like      AGAGTTATTCATACTCACTCTCATGCCCATGCACATGGTTCAACAACAATGA-TGGCTGA     536
                                  **      **  *** ** **              *  **    **  *  *   ***

                                                                      ______________________
NM_001325745.1-NtZIP5A          TTCCAGTTCTGAGCTCCTTCGTTATCGTGTTGTATCTCAGGTATTGGAAATGGGGATAAT     584
AB505626.1-NtZIP1               TTCCAGTTCTGAGCTCCTTCGTTATCGTGTTGTATCTCAGGTATTGGAAATGGGGATAAT     584
XM_009770855.1-NsZIP5-like      TTCTAGTTCCGAGCTCCTTCGTTATCGTGTTGTATCTCAGGTATTGGAAATGGGGATAAT     596
XM_016593570.1-NtZIP5B          TTCTAGTTCCGAGCTCCTTCGTTATCGTGTTGTATCTCAGGTATTGGAAATGGGGATAAT     596
XM_019381545.1-NaZIP5-like      TTCTAGTTCTGAGCTCCTTCGTTATCGTGTTGTATCTCAGGTTTTGGAGTTGGGGATAAT     596
XM_009612040.2-NtomZIP5         TTCTGATTCCGAGCTCCTTCGTTATCGTGTTATATCTCAGGTTTTGGAGGTGGGGATAAT     590
XM_009776848.1-NsZIP5-like      TTCTGATTCCGAGCTCCTTCGTTATCGTGTTATATCTCAGGTTTTGGAGTTGGGGATAAT     593
XM_006353660.2-StZIP5-like      TTCGGAGTCTGAACTCCTTCGATATCGTGTTATCTCTCAGGTTTTGGAACTTGGGATAAT     584
XM_015225484.1-SpZIP5-like      TTCCGAGTCTGAACTCCTCCGTTACCGTGTTATTTCTCAGGTTTTGGAACTTGGGATAAT     584
XM_006353661.2-StZIP5-like      TTCTAATTCGGAGCTTCTTCGTTATCGTGTTATTGCTCAGGTTTTGGAATTGGGGATAAT     596
                                ***    ** ** ** ** ** ** ****** *  ******* *****  * ********

                                               TM IV
                                ________________________________________                __
NM_001325745.1-NtZIP5A          AGTGCATTCTGTGATAATAGGAATAGCTTTGGGTGCTTCTGAAAGTCCCAAAACCATAAG     644
AB505626.1-NtZIP1               AGTGCATTCTGTGATAATAGGAATAGCTTTGGGTGCTTCTGAAAGTCCCAAAACCATAAG     644
XM_009770855.1-NsZIP5-like      AGTGCATTCTGTGATAATAGGAATAGCTTTGGGTGCTTCTGAAAGTCCCAAAACCATAAG     656
XM_016593570.1-NtZIP5B          AGTGCATTCTGTGATAATAGGAATAGCTTTGGGTGCTTCTGAAAGTCCCAAAACCATAAG     656
XM_019381545.1-NaZIP5-like      AGTGCACTCTGTGATAATAGGAATAGCTTTGGGTGCTTCTGAAAGTCCCAAAACCATAAG     656
XM_009612040.2-NtomZIP5         AGTACATTCTGTGATAATAGGAATAGCTTTGGGTGCTTCTGAAAGTCCCAAAACCATAAG     650
XM_009776848.1-NsZIP5-like      AGTGCACTCTGTGATAATAGGAATAGCTTTGGGTGCTTCTGAAAGTCCCAAAACCATAAG     653
XM_006353660.2-StZIP5-like      AGTGCACTCTGTGATTATTGGAATAGCTTTGGGTGCTTCTGAAAGTCCCAAAACTATAAA     644
XM_015225484.1-SpZIP5-like      AGTGCACTCTGTGATTATTGGAATAGCTTTGGGTGCTTCTGAAAGTCCAAAAACCATAAA     644
XM_006353661.2-StZIP5-like      AGTGCACTCAGTGATAATAGGAATAGCTTTGGGTGCTTCTGAAAGTCCCAAAACTATAAA     656
                                *** ** ** ***** ** ***************************** ***** ****

                                                 TM V
                                ____________________________________________________________
```

```
NM_001325745.1-NtZIP5A      GCCTCTTGTTGCTGCTTTGACTTTTCATCAATTTTTCGAAGGCATGGGACTTGGTGGATG   704
AB505626.1-NtZIP1           GCCTCTTGTTGCTGCTTTGACTTTTCATCAATTTTTCGAAGGCATGGGACTTGGTGGATG   704
XM_009770855.1-NsZIP5-like  GCCTCTTGTTGGTGCTTTGACTTTTCATCAATTTTTCGAAGGCATGGGACTTGGTGGATG   716
XM_016593570.1-NtZIP5B      GCCTCTTGTTGGTGCTTTGACTTTTCATCAATTTTTCGAAGGCATGGGACTTGGTGGATG   716
XM_019381545.1-NaZIP5-like  GCCTCTTGTTGGTGCTTTGACTTTTCATCAATTTTTCGAAGGCATGGGACTTGGTGGATG   716
XM_009612040.2-NtomZIP5     GCCTCTTGTTGCTGCTTTGACTTTTCATCAATTTTTCGAGGGCATGGGACTTGGTGGATG   710
XM_009776848.1-NsZIP5-like  GCCTCTTGTTGGTGCTTTGACTTTTCATCAATTTTTTGAAGGAATGGGACTTGGTGGATG   713
XM_006353660.2-StZIP5-like  GCCTCTTGTTGCTGCTTTGACTTTTCATCAATTTTTCGAAGGCTTGGGACTTGGTGGATG   704
XM_015225484.1-SpZIP5-like  GCCTCTTGTTGCTGCTTTGACTTTTCATCAATTTTTCGAAGGCTTGGGACTTGGTGGATG   704
XM_006353661.2-StZIP5-like  GCCACTGGTTGCTGCTTTGACTTTTCATCAGTTTTTTGAAGGCATGGGACTTGGTGGATG   716
                            *** ** **** ***************** ***** ** **  ***************

                                                            TM VI
NM_001325745.1-NtZIP5A      TATTGCTCAGGCAAAATTCAAGACTCGTGCAGTGGCAATAATGGCTTTATTTTTCTCTCT   764
AB505626.1-NtZIP1           TATTGCTCAGGCAAAATTCAAGACTCGTGCAGTGGCAATAATGGCTTTATTTTTCTCTCT   764
XM_009770855.1-NsZIP5-like  CATTGCTCAGGCAAAATTCAAGACTCGGGCAGTGGCAATAATGGCTTTATTTTTCTCACT   776
XM_016593570.1-NtZIP5B      CATTGCTCAGGCAAAATTCAAGACTCGGGCAGTGGCAATAATGGCTTTATTTTTCTCACT   776
XM_019381545.1-NaZIP5-like  CATTGCTCAGGCAAAATTCAAGACTCGGGCAGTGGCAATAATGGCTTTATTTTTCTCTCT   776
XM_009612040.2-NtomZIP5     CATTGCTCAGGCAAAATTTAAGACTCGTGCAGTGGCAATAATGACTTTATTTTTCTCTCT   770
XM_009776848.1-NsZIP5-like  CATTGCTCAGGCAAAATTCAAGAATCGTGCAGTGGTAATAATGACTTTATTTTTCTCTCT   773
XM_006353660.2-StZIP5-like  CATTGCTCAGGCAAAATTGAAGACTCGCACAATTGCAATAATGGCTTTATTTTTCTCGCT   764
XM_015225484.1-SpZIP5-like  CATTGCTCAGGCAAAATTGAAGAGTCGGACAATCGCAATAATGGCTTTATTTTTCTCGCT   764
XM_006353661.2-StZIP5-like  CATTGCTCAGGCAAAATTGAAGTCTCGTGCAATTGCAATAATGGCTTTATTTTTCTCCCT   776
                             **************** ***   ***   ** * * ******* ************* **

NM_001325745.1-NtZIP5A      TACAACTCCAGTCGGTATTGCAATTGGATTAGGAATAACAAATGTTTACGATGAAAACAG   824
AB505626.1-NtZIP1           TACAACTCCAGTCGGTATTGCAATTGGATTAGGAATAACAAATGTTTACGATGAAAACAG   824
XM_009770855.1-NsZIP5-like  TACAACTCCAGTGGGTATTGCAATTGGACTAGGAATAACAAATGTTTACGATGAAAACAG   836
XM_016593570.1-NtZIP5B      TACAACTCCAGTGGGTATTGCAATTGGACTAGGAATAACAAATGTTTACGATGAAAACAG   836
XM_019381545.1-NaZIP5-like  TACAACTCCAGTGGGAATTGCAATTGGACTAGGAATAACAAATGTTTACGATGAAAACAG   836
XM_009612040.2-NtomZIP5     CACAACTCCAATCGGAATTGGAATTGGATTAGGAATAACAAATTCTTACGATGAAAACAG   830
XM_009776848.1-NsZIP5-like  CACAACTCCAATAGGAATTGGAATTGGACTAGGAATAACAAATTCTTACGATGAAAACAG   833
XM_006353660.2-StZIP5-like  CACAACTCCAATTGGAATAGGAATCGGACTAGGAATAACAAATGTTTACGATGAAAACAG   824
XM_015225484.1-SpZIP5-like  CACAACTCCAATTGGAATAGGAATTGGACTAGGAATAACAAATGTTTACGATGAGAACAG   824
XM_006353661.2-StZIP5-like  CACAACTCCAGTTGGAATTGCAGTTGGACTAGCAATAACAAATGTTTACGATGAGAATAG   836
                             ********* * ** ** * * * *** *** **********   ********* ** **

NM_001325745.1-NtZIP5A      TCCAACGGCTCTTATTGTGGAAGGAGTATTTAATTCAGCATCAGCTGGTATCTTGATTTA   884
AB505626.1-NtZIP1           TCCAACGGCTCTTATTGTGGAAGGAGTATTTAATTCAGCATCAGCTGGTATCTTGATTTA   884
XM_009770855.1-NsZIP5-like  TCCAACGGCTCTCATTGTGGAAGGAGTATTTAATTCAGCATCAGCTGGTATCTTGATTTA   896
XM_016593570.1-NtZIP5B      TCCAACGGCTCTCATTGTGGAAGGAGTATTTAATTCAGCATCAGCTGGTATCTTGATTTA   896
XM_019381545.1-NaZIP5-like  TCCGACGGCTCTCATTGTAGAAGGAGTATTTAATTCAGCATCAGCTGGTATCTTGATTTA   896
XM_009612040.2-NtomZIP5     TCCAACGGCTCTTATTGTGGAAGGAGTATTTAATTCAGCATCAGCTGGTATCTTGATTTA   890
XM_009776848.1-NsZIP5-like  TCCAACAGCTCTTATTGTGGAAGGAGTATTTAATTCAGCATCAGCTGGTATCTTGATTTA   893
XM_006353660.2-StZIP5-like  TCCAACGGCTCTTATTGTGGAAGGAGTATTTAATTCAGCTTCAGCTGGCATTTTAATTTA   884
XM_015225484.1-SpZIP5-like  TCCAACGGCTCTTATTGTGGAAGGAGTATTTAATTCGGCTTCCGCTGGCATTTTGATTTA   884
XM_006353661.2-StZIP5-like  TCCAACGGCTCTTATCGTGGAAGGAATATTTAATTCAGCTTCAGCTGGCATCTTGATTTA   896
                            *** ** ***** ** ** ****** ********** ** ** ***** ** ** *****
                                           TM VII
NM_001325745.1-NtZIP5A      TATGGCACTTGTTGATTTTTAGCTGCTGATTTTATGCATCCAAGAATGCAAGGCAATGG   944
AB505626.1-NtZIP1           TATGGCACTTGTTGATTTTTAGCTGCTGATTTTATGCATCCAAGAATGCAAGGCAATGG   944
XM_009770855.1-NsZIP5-like  TATGGCATTAGTTGATTTTTGGCTGCTGATTTTATGCATCCAAGAATGCAAGGCAATGG   956
XM_016593570.1-NtZIP5B      TATGGCATTAGTTGATTTTTGGCTGCTGATTTTATGCATCCAAGAATGCAAGGCAATGG   956
XM_019381545.1-NaZIP5-like  TATGGCATTAGTTGATTTTTGGCTGCTGATTTTATGCATCCAAGAATGCAAGGCAATGG   956
XM_009612040.2-NtomZIP5     TATGGCATTAGTTGATTTTTAGCTGCCGATTTTATGCATCCAAGAATGCAAGGCAATGG   950
XM_009776848.1-NsZIP5-like  TATGGCACTTGTCGATTTTTAGCTGCTGATTTTATGCATCCAAGAATGCAAGGCAATGG   953
XM_006353660.2-StZIP5-like  TATGGCATTAGTTGATTTTTAGCTGCCGATTTTATGCATCCCAGAATGCAAGGCAATGG   944
XM_015225484.1-SpZIP5-like  TATGGCACTAGTTGATTTTTAGCAGCCGATTTTATGAATCCAAGAATGCAAGGCAATGG   944
XM_006353661.2-StZIP5-like  TATGGCGCTCGTTGACTTTTTAGCTGCCGATTTTATGCATCCAAGAATGCAAGGCAATGG   956
                            ******   * ** ** ***** ** ** ********* **** ****************
                                                     TM VIII
NM_001325745.1-NtZIP5A      AAAGCTTCAATTAGGGGCAAATATTTCACTTCTTCTCGGTGCTGGACTCATGGCTCTCAT   1004
AB505626.1-NtZIP1           AAAGCTTCAATTAGGGGCAAATATTTCACTTCTTCTCGGTGCTGGACTCATGGCTCTCAT   1004
XM_009770855.1-NsZIP5-like  AAAACTTCAATTAGGTGCCAATGTTTCACTTCTTCTTGGTGCTGGACTCATGGCTCTCAT   1016
XM_016593570.1-NtZIP5B      AAAACTTCAATTAGGTGCCAATGTTTCACTTCTTCTTGGTGCTGGACTCATGGCTCTCAT   1016
XM_019381545.1-NaZIP5-like  AAAACTTCAATTAGGTGCCAATATTTCACTTCTTCTTGGTGCTGGACTCATGGCTCTCAT   1016
XM_009612040.2-NtomZIP5     AAAACTTCAATTAGGAGCCAATATTTCACTTCTTCTTGGTGCTGGACTTATGTCTCTCTT   1010
XM_009776848.1-NsZIP5-like  AAAACTTCAATTAGGAGCCAATATTTCACTTCTTCTTGGTGCTGGACTTATGTCTCTCCT   1013
XM_006353660.2-StZIP5-like  AAAGCTTCAATTAGGGGCCAATATTTCACTTCTTCTTGGTGCTGGACTCATGTCTCTGTT   1004
XM_015225484.1-SpZIP5-like  AAAGCTTCAATTAGGGGCCAATATTTCACTTCTTCTTGGTGCTGGACTCATGTCTATGTT   1004
XM_006353661.2-StZIP5-like  AAAGCTTCAATTAGGGGCTAATGTTTCACTTCTTCTTGGCGCTGGTCTTATGTCTATGTT   1016
                            *** *********** ** *** ************* ** ***** ** *** ** *   *
```

```
NM_001325745.1-NtZIP5A          AGCCAAATGGGCTTAA      1020
AB505626.1-NtZIP1               AGCCAAATGGGCTTAA      1020
XM_009770855.1-NsZIP5-like      AGCCAAATGGGCTTAA      1032
XM_016593570.1-NtZIP5B          AGCCAAATGGGCTTAA      1032
XM_019381545.1-NaZIP5-like      AGCCAAATGGGCTTAA      1032
XM_009612040.2-NtomZIP5         AGCCAAATGGGCTTAA      1026
XM_009776848.1-NsZIP5-like      AGCCAAATGGGCTTAA      1029
XM_006353660.2-StZIP5-like      AGCCAAATGGGCTTAA      1020
XM_015225484.1-SpZIP5-like      AGCCAAATGGGCTTAA      1020
XM_006353661.2-StZIP5-like      AGCCAAATGGGCTTAA      1032
                                ****************
```

(a2) The sequence Acc no XM_016593570.1 (named later in this study as *NtZIP5B*) was used as a query to search through NCBI data base for genes with the highest homology.

The list of tobacco genes identified as the best Blast Hits are given in the Table below;

Description	Max score	Total score	Query cover	E value	Ident	Accession
PREDICTED: *Nicotiana sylvestris* zinc transporter 5-like (LOC104220059), mRNA	1901	1901	100%	0.0	99%	XM_009770855.1
PREDICTED: *Nicotiana attenuata* zinc transporter 5-like (LOC109217305), mRNA	1685	1685	100%	0.0	96%	XM_019381545.1
***Nicotiana tabacum* zinc transporter 5-like (LOC107803903), mRNA [in this study named as *NtZIP5A*]**	**1629**	**1629**	**100%**	**0.0**	**95%**	**NM_001325745.1**
Nicotiana tabacum NtZIP1 mRNA for metal transporter, (Sano et al., 2012) In this study renamed to *NtZIP5A*	1629	1629	100%	0.0	95%	AB505626.1
PREDICTED: *Nicotiana tomentosiformis* zinc transporter 5 (LOC104104056), mRNA	1424	1424	100%	0.0	92%	XM_009612040.2
PREDICTED: *Nicotiana sylvestris* zinc transporter 5-like (LOC104225083), mRNA	1389	1389	99%	0.0	91%	XM_009776848.1
PREDICTED: *Nicotiana attenuata* zinc transporter 5-like (LOC109222763), partial mRNA	1144	1144	70%	0.0	95%	XM_019387083.1
PREDICTED: *Nicotiana tabacum* zinc transporter 5-like (LOC107774468), mRNA	1138	1138	81%	0.0	91%	XM_016594002.1
PREDICTED: *Solanum tuberosum* zinc transporter 5-like (LOC102600170), mRNA	1125	1125	100%	0.0	86%	XM_006353661.2

Alignments of ORF+STOP nucleotide sequences between the query sequence (**Acc no XM_016593570.1,** named later as ***NtZIP5B*)** and sequences of ~~tobacco~~ genes identified as the best hits. Sequences were aligned using Clustal Omega https://www.ebi.ac.uk/Tools/msa/clustalo/. The prediction of membrane regions was performed using Phobius programme (http://phobius.sbc.su.se/), and indicated as lines above the sequences, and numbered TM I–VIII respectively. Nucleotide sequences coding the transmembrane domains (TM) are marked with light grey; identical nucleotide with dark grey. Dashes indicate gaps.

```
XM_016593570.1-NtZIP5B        ATGACAAAGTTAGAAAAAGTAGTTTTTTGGTACATTCTCTTGCTTCTTCCTGCTATAGTA    60
XM_009770855.1-NsZIP5-like    ATGACAAAGTTAGAAAAAGTAGTTTTTTGGTACATTCTCTTGCTTCTTCCTGCTATAGTA    60
XM_019381545.1-NaZIP5-like    ATGACAAAGTTGGAAAAAGTTGTATTTTGGTACATTCTCTTGCTTCTTCCTGCTATAGTA    60
NM_001325745.1-NtZIP5A        ATGACAAAGTTAGAAAAAGTTATTTTTTGGTACATTCTCTTGCTTCTTCCTGCTATAGTA    60
AB505626.1-NtZIP1             ATGACAAAGTTAGAAAAAGTTATTTTTTGGTACATTCTCTTGCTTCTTCCTGCTATAGTA    60
XM_009612040.2-NtomZIP5       ATGGCAAAGTTAGAAAAAGTTGTTTTTTGGTACATTCTCGTACTTCTTCCTGCTATAGTA    60
XM_009776848.1-NsZIP5-like    ATGGAAAAGTTAAAAAAATGTTATTTTCTGGTACATTCTCGTTCTTTTACCTGCTATAGTA   60
XM_019387083.1-NaZIP5-like    ATGACAAAGTTGGAAAAAGTTGTATTTTGGTACATTCTCTTGCTTCTTCCTGCTATAGTA    60
XM_016594002.1-NtZIP5-like    ------------------------------------------------------------    0
XM_006353661.2-StZIP5-like    ATGGCAAAGATGGAAAAAATTGTATTTTGGTACATTCTATTGCTTCTTCCCGCAATAGTA    60


XM_016593570.1-NtZIP5B        TTAGGAGAATGTACTTGTGATTCTGAGGATGAAGAAAGAAACAAACCTGAAGCACTGAAA    120
XM_009770855.1-NsZIP5-like    TTAGGAGAATGTACTTGTGATTCTGAGGATGAAGAAAGAAACAAACCTGAAGCACTGAAA    120
XM_019381545.1-NaZIP5-like    TTAGGAGAATGTACATGTGAATCTGAGGATGAAGAAAGAAACAAAACTGAAGCACTGAAA    120
NM_001325745.1-NtZIP5A        TTAGGCGAATGTACTTGTGATTCTGAGGATGAAGAAAGAAACAAAACTGAAGCACTGAAG    120
AB505626.1-NtZIP1             TTAGGCGAATGTACTTGTGATTCTGAGGATGAAGAAAGAAACAAAACTGAAGCACTGAAG    120
XM_009612040.2-NtomZIP5       TTAGGAGAATGTACTTGTGATTCTGATGAAGAAGAAAGAAACAAAACTGAAGCATTAAAA    120
XM_009776848.1-NsZIP5-like    TTAGGAGAATGTACATGTGATTCTGAGGATGAAGAAAGAAACAAAGCTGAAGCACTGAAG    120
XM_019387083.1-NaZIP5-like    TTAGGAGAATGTACATGTGAATCTGAGGATGAAGAAAGAAACAAAACTGAAGCACTGAAA    120
XM_016594002.1-NtZIP5-like    ------------------------------------------------------------    0
XM_006353661.2-StZIP5-like    TTAGGCGAATGTACTTGTGATTCGGAGGATGAAGAAAGAAACAAAAGTGAGGCACTCAAG    120
```

TM I

```
XM_016593570.1-NtZIP5B        TATAAAATGGTAGCAATCGCTTCCATTTTGATTGCCAGTGCAATTGGGGTTTGTATTCCT    180
XM_009770855.1-NsZIP5-like    TATAAAATGGTAGCAATCGCTTCCATTTTGATTGCCAGTGCAATTGGGGTTTGTATTCCT    180
XM_019381545.1-NaZIP5-like    TATAAAATGGTAGCAATCGCTACCATTTTGGTAGCCAGTGCAATTGGGGTTTGTATTCCT    180
NM_001325745.1-NtZIP5A        TATAAAATGGTAGCAATCGCTTCCATTCTGATTGCCAGTGCAATTGGGGTTTGTATTCCT    180
AB505626.1-NtZIP1             TATAAAATGGTAGCAATCGCTTCCATTCTGATTGCCAGTGCAATTGGGGTTTGTATTCCT    180
XM_009612040.2-NtomZIP5       TATAAAATGGTAGCAGTCGCTTCCATTTTAGTTGCCAGTGCATTAGGGGTTTGTATTCCT    180
XM_009776848.1-NsZIP5-like    TATAAAATGGTAGCAGTTGCTTCCATTTTAGTAGCTAGTGCATTAGGGGTTTGTATTCCT    180
XM_019387083.1-NaZIP5-like    TATAAAATGGTAGCAATCGCTACCATTTTGGTAGCCAGTGCAATTGGGGTTTGTATTCCT    180
XM_016594002.1-NtZIP5-like    ------------------------------------------ATGGAGGATTGTACGAGG    18
XM_006353661.2-StZIP5-like    TACAAAATGGCAGCACTCGCTTCCATTTTGGTAGCGAGTGCAATTGGGGTGTGTATTCCT    180
                                                                         * * **  ****


XM_016593570.1-NtZIP5B        ---GTACTAGGAAAAGCAATTCCAGCTTTGAGCCCAGAGAAGAATTTCTTCTTCATAATC    237
XM_009770855.1-NsZIP5-like    ---GTACTAGGAAAAGCAATTCCAGCTTTGAGCCCAGAGAAGAATTTCTTCTTCATAATC    237
XM_019381545.1-NaZIP5-like    ---GTACTAGGAAAAGCAATTCCAGCTTTGAGCCCAGAGAAGAATTTCTTCTTCATAATC    237
NM_001325745.1-NtZIP5A        ---GTACTTGGAAAAGCAATTCCAGCTTTAAGCCCAGAGAAGAATTTCTTCTTCATAATC    237
AB505626.1-NtZIP1             ---GTACTTGGAAAAGCAATTCCAGCTTTAAGCCCAGAGAAGAATTTCTTCTTCATAATC    237
XM_009612040.2-NtomZIP5       ---GTACTTGGAAAAGCAATTCCAGCTTTGAGCCCAGAGAAGAATTTATTTTTTATAATC    237
XM_009776848.1-NsZIP5-like    ---GTACTAGGAAAAGCAATTCCAGCTTTGAGTCCAGAGAAGAATTTATTTTTCATAATC    237
XM_019387083.1-NaZIP5-like    ---GTACTAGGAAAAGCAATTCCAGCTTTGAGCCCAGAGAAGAATTTCTTCTTCATAATC    237
XM_016594002.1-NtZIP5-like    TTAATTTTTGAAAAAATAATACCAGCTTTGAGCCCAGAGAAGAATTTATTTTTTATAATC    78
XM_006353661.2-StZIP5-like    ---GTTCTGGGCAAGGTGATTCCCGCCTTAAGCCCAGAGAGGAATTTCTTCTTTATAATT    237
                                  *  * *  **    ** ** ** ** ** ******* ****** ** ** *****
```

TM II

```
XM_016593570.1-NtZIP5B        AAAGCTTTTGCTGCTGGTGTGATCCTCGCGACAGGGTTTATACATGTACTCCCTGATGCA    297
XM_009770855.1-NsZIP5-like    AAAGCTTTTGCTGCTGGTGTGATCCTCGCGACAGGGTTTATACATGTACTCCCTGATGCA    297
XM_019381545.1-NaZIP5-like    AAAGCGTTCGCGGCGGGTGTGATCCTCGCGACAGGGTTTATACATGTACTCCCCGATGCA    297
NM_001325745.1-NtZIP5A        AAAGCTTTTGCTGCTGGTGTGATCCTCGCGACAGGGTTTATACATGTACTTCCTGATGCT    297
AB505626.1-NtZIP1             AAAGCTTTTGCTGCTGGTGTGATCCTCGCGACAGGGTTTATACATGTACTTCCTGATGCT    297
XM_009612040.2-NtomZIP5       AAAGCTTTCGCTGCTGGTGTGATCCTCGCGACAGGGTTTATACATGTATTACCTGATGCA    297
XM_009776848.1-NsZIP5-like    AAAGCGTTCGCGGCGGGTGTGATCCTCTCGACAGGGTTTATACATGTATTACCTGATGCA    297
XM_019387083.1-NaZIP5-like    AAAGCGTTCGCGGCGGGTGTGATCCTCGCGACAGGGTTTATACATGTACTCCCCGATGCA    297
XM_016594002.1-NtZIP5-like    AAAGCTTTCGCTGCTGGTGTGATCCTCGCGACAGGGTTTATACATGTATTACCTGATGCA    138
XM_006353661.2-StZIP5-like    AAAGCTTTCGCGGCTGGTGTTATCCTATCAACGGGGTTTATACATGTACTTCCTGATGCA    297
                              ***** ** ** ** ***** *****  * ** *************** * ** *****
```

```
XM_016593570.1-NtZIP5B          TTTGAAAGCTTAACATCGCCATGTTTGAAAGAGAATCCGTGGGGAAATTTTCCATTCAGT    357
XM_009770855.1-NsZIP5-like      TTTGAAAGCTTAACATCGCCATGTTTGAAAGAGAATCCGTGGGGAAATTTTCCATTTAGT    357
XM_019381545.1-NaZIP5-like      TATGAAAGCTTGACATCGCCATGTTTGAAAGAAAATCCATGGGGAAATTTTCCTTTTAGT    357
NM_001325745.1-NtZIP5A          TTTGAAAGCTTAACATCGCCATGTTTGAAAGAGAATCCGTGGGGAAATTTTCCTTTTAGT    357
AB505626.1-NtZIP1               TTTGAAAGCTTAACATCGCCATGTTTGAAAGAGAATCCGTGGGGAAATTTTCCTTTTAGT    357
XM_009612040.2-NtomZIP5         TATAAAAGTTTAACATCGCCATATTTGAAAGAAAATCCGTGGGGAGATTTTCCTTTTAGT    357
XM_009776848.1-NsZIP5-like      TATGAAAGTTTAACGTCGCCATGTTTGAAAGAAAATCCGTGGGGAGATTTTCCATTCAGT    357
XM_019387083.1-NaZIP5-like      TATGAAAGCTTGACATCGCCATGTTTGAAAGAAAATCCATGGGGAAATTTTCCTTTTAGT    357
XM_016594002.1-NtZIP5-like      TATAAAAGTTTAACATCGCCATGTTTGAAAAAACATCCGTGGGGAGATTTTCCTTTTAGT    198
XM_006353661.2-StZIP5-like      TTTGAAAGTTTGACATCTCCGTGTTTGAAAGAGAATCCATGGGGAAATTTCCCCTTTTGT    357
                                * * **** ** ** ** ** * ******* *  **** ****** **** ** **  **
```

TM III

```
XM_016593570.1-NtZIP5B          GGATTTATCGCAATGGTTTCTGCAATGGGAACTCTAATGGTGGACACTTATGCAACTTCA    417
XM_009770855.1-NsZIP5-like      GGATTTATCGCAATGGTTTCTGCAATGGGAACTCTAATGGTGGACACTTATGCAACTTCA    417
XM_019381545.1-NaZIP5-like      GGATTTATTGCAATGGTTTCTGCTATGGGAACTCTTATGGTGGATACTTATGCAACTTCA    417
NM_001325745.1-NtZIP5A          GGATTTATTGCAATGGTTTCTGCAATGGGAACTCTTATGGTGGATACTTATGCAACTTCA    417
AB505626.1-NtZIP1               GGATTTATTGCAATGGTTTCTGCAATGGGAACTCTTATGGTGGATACTTATGCAACTTCA    417
XM_009612040.2-NtomZIP5         GGATTTATTGCAATGGTTTCTGCGATGGCGACTCTGATGGTGGATACTTATGCAACTTCA    417
XM_009776848.1-NsZIP5-like      GGATTTATTGCAATGGTTTCTGCAATGGCGACTCTGATGGTGGATACTTATGCAACTTCG    417
XM_019387083.1-NaZIP5-like      GGATTTATTGCAATGGTTTCTGCTATGGGAACTCTTATGGTGGATACTTATGCAACTTCA    417
XM_016594002.1-NtZIP5-like      GGATTTATTGCAATGATTTCTGCAATGGCGACTCTGATGGTGGATACTTATGCAACTTCA    258
XM_006353661.2-StZIP5-like      GGATTTGTTGCTATGGTTTCTGCAATGGCGACTCTGATGGTGGATACTTATGCAACATCT    417
                                ****** * ** *** ******* ****  ***** ******** *********** **

XM_016593570.1-NtZIP5B          TATTTCAGTAACAAAAATGATACGAAAAATGGATTGGTG---GCTCAGTCTGGAGATGAA    474
XM_009770855.1-NsZIP5-like      TATTTCAGTAACAAAAATGATACGAAAAATGGATTGGTG---GCTCAGTCTGGAGATGAA    474
XM_019381545.1-NaZIP5-like      TATTTCAGAAACAAAAATGACACGAAAAATGGATTGGTG---GCTCAGTCTGGAGATGAA    474
NM_001325745.1-NtZIP5A          TATTTCAGTAACAAAAATGACACGAAAAATGGATTGGTG---GCTCAGTCTGGAGATGAA    474
AB505626.1-NtZIP1               TATTTCAGTAACAAAAATGACACGAAAAATGGATTGGTG---GCTCAGTCTGGAGATGAA    474
XM_009612040.2-NtomZIP5         TATTATAAGAATAAAT---CTGCGAAAAATGGTGTGGTG---GCTCAGTCTGGAGATGAA    471
XM_009776848.1-NsZIP5-like      TATTATAAGAATAAAT---CTGTGAAAAATGGTGTGGTG---GCTCAGTCTGGAGATGAA    471
XM_019387083.1-NaZIP5-like      TATTTCAGAAACAAAAATGACACGAAAAATGGATTGGTG---GCTCAGTCTGGAGATGAA    474
XM_016594002.1-NtZIP5-like      TATTATAAGAATAAAT---CTGCGAAAAATGGTGTGGTG---GCTCAGTCTGGAGATGAA    312
XM_006353661.2-StZIP5-like      TATTACAATAAGAAGAA---TTTGAAAAATGGAGTGGTGACGACTGAGGGTGGAGATGAA    474
                                ****  *  ** **         *********  *****    ** **  **********

XM_016593570.1-NtZIP5B          GGAGGAGCTATACATGTTCATTCACATGGCCATGCACATGGTTCATCATCACTGCTGGGT    534
XM_009770855.1-NsZIP5-like      GGAGGAGCTATACATGTTCATTCACATGGCCATGCACATGGTTCATCATCACTGCTGGGT    534
XM_019381545.1-NaZIP5-like      GGAGGAGCTATTCATGTTCATTCACATGGCCATGCACATGGTGCATCATCAATGATGGGT    534
NM_001325745.1-NtZIP5A          GGAGGAGCTATTCATGTTCATTCAC------------ATGGTTCAGCATCACTGATGGGT    522
AB505626.1-NtZIP1               GGAGGAGCTATTCATGTTCATTCAC------------ATGGTTCAGCATCACTGATGGGT    522
XM_009612040.2-NtomZIP5         GAATTAGTT---AATGTTCATTCACATGGCCATGTACATGGCTCAACATCAATGATTGGT    528
XM_009776848.1-NsZIP5-like      GAAGGAGTTATTCATGTTCATTCACATGGCCATGCACATGGTTCAACATCAATGATTGGT    531
XM_019387083.1-NaZIP5-like      GGAGGAGCTATTCATGTTCATTCACATGGCCATGCACATGGTGCATCATCAATGATGGGT    534
XM_016594002.1-NtZIP5-like      GAATTAGTT---AATGTTCATTCACATGGCCATGTACATGGTTCAACATCAATGATTGGT    369
XM_006353661.2-StZIP5-like      GAAAGAGTTATTCATACTCACTCTCATGCCCATGCACATGGTTCAACAACAATGATGGCT    534
                                * *  ** *    **  *** ** *            ****  ** ** ** ** * * *

XM_016593570.1-NtZIP5B          GATTCTAGTTCCGAGCTCCTTCGTTATCGTGTTGTATCTCAGGTATTGGAAATGGGGATA    594
XM_009770855.1-NsZIP5-like      GATTCTAGTTCCGAGCTCCTTCGTTATCGTGTTGTATCTCAGGTATTGGAAATGGGGATA    594
XM_019381545.1-NaZIP5-like      GATTCTAGTTCTGAGCTCCTTCGTTATCGTGTTGTATCTCAGGTTTTGGAGTTGGGGATA    594
NM_001325745.1-NtZIP5A          GATTCCAGTTCTGAGCTCCTTCGTTATCGTGTTGTATCTCAGGTATTGGAAATGGGGATA    582
AB505626.1-NtZIP1               GATTCCAGTTCTGAGCTCCTTCGTTATCGTGTTGTATCTCAGGTATTGGAAATGGGGATA    582
XM_009612040.2-NtomZIP5         GATTCTGATTCCGAGCTCCTTCGTTATCGTGTTATATCTCAGGTTTTGGAGGTGGGGATA    588
XM_009776848.1-NsZIP5-like      GATTCTGATTCCGAGCTCCTTCGTTATCGTGTTATATCTCAGGTTTTGGAGTTGGGGATA    591
XM_019387083.1-NaZIP5-like      GATTCTAGTTCTGAGCTCCTTCGTTATCGTGTTGTATCTCAGGTTTTGGAGTTGGGGATA    594
XM_016594002.1-NtZIP5-like      GATTCTGATTCCGAGCTCCTTCGTTATCGTGTTATATCTCAGGTTTTGGAGTTGGGGATA    429
XM_006353661.2-StZIP5-like      GATTCTAATTCGGAGCTTCTTCGTTATCGTGTTATTGCTCAGGTTTTGGAATTGGGGATA    594
                                *****   *** ***** *************** *  ******* *****  ********
```

TM IV

```
XM_016593570.1-NtZIP5B          ATAGTGCATTCTGTGATAATAGGAATAGCTTTGGGTGCTTCTGAAAGTCCCAAAACCATA    654
XM_009770855.1-NsZIP5-like      ATAGTGCATTCTGTGATAATAGGAATAGCTTTGGGTGCTTCTGAAAGTCCCAAAACCATA    654
XM_019381545.1-NaZIP5-like      ATAGTGCACTCTGTGATAATAGGAATAGCTTTGGGTGCTTCTGAAAGTCCCAAAACCATA    654
NM_001325745.1-NtZIP5A          ATAGTGCATTCTGTGATAATAGGAATAGCTTTGGGTGCTTCTGAAAGTCCCAAAACCATA    642
AB505626.1-NtZIP1               ATAGTGCATTCTGTGATAATAGGAATAGCTTTGGGTGCTTCTGAAAGTCCCAAAACCATA    642
XM_009612040.2-NtomZIP5         ATAGTACATTCTGTGATAATAGGAATAGCTTTGGGTGCTTCTGAAAGTCCCAAAACCATA    648
XM_009776848.1-NsZIP5-like      ATAGTGCACTCTGTGATAATAGGAATAGCTTTGGGTGCTTCTGAAAGTCCCAAAACCATA    651
XM_019387083.1-NaZIP5-like      ATAGTGCACTCTGTGATAATAGGAATAGCTTTGGGTGCTTCTGAAAGTCCCAAAACCATA    654
XM_016594002.1-NtZIP5-like      ATAGTACATTCTGTGATAATAGGAATAGCTTTGGGTGCTTCTGAAAGTCCCAAAACCATA    489
XM_006353661.2-StZIP5-like      ATAGTGCACTCAGTGATAATAGGAATAGCTTTGGGTGCTTCTGAAAGTCCCAAAACTATA    654
                                ***** ** ** ******************************************** ***
```

TM V

```
XM_016593570.1-NtZIP5B         AGGCCTCTTGTTGGTGCTTTGACTTTTCATCAATTTTTCGAAGGCATGGGACTTGGTGGA     714
XM_009770855.1-NsZIP5-like     AGGCCTCTTGTTGGTGCTTTGACTTTTCATCAATTTTTCGAAGGCATGGGACTTGGTGGA     714
XM_019381545.1-NaZIP5-like     AGGCCTCTTGTTGGTGCTTTGACTTTTCATCAATTTTTCGAAGGCATGGGACTTGGTGGA     714
NM_001325745.1-NtZIP5A         AGGCCTCTTGTTGCTGCTTTGACTTTTCATCAATTTTTCGAAGGCATGGGACTTGGTGGA     702
AB505626.1-NtZIP1              AGGCCTCTTGTTGCTGCTTTGACTTTTCATCAATTTTTCGAAGGCATGGGACTTGGTGGA     702
XM_009612040.2-NtomZIP5        AGGCCTCTTGTTGCTGCTTTGACTTTTCATCAATTTTTCGAGGGCATGGGACTTGGTGGA     708
XM_009776848.1-NsZIP5-like     AGGCCTCTTGTTGGTGCTTTGACTTTTCATCAATTTTTTGAAGGAATGGGACTTGGTGGA     711
XM_019387083.1-NaZIP5-like     AGGCCTCTTGTTGGAGCTTTGACTTTTCATCAATTTTTCGAAGGCATGGGACTTGGTGGA     714
XM_016594002.1-NtZIP5-like     AGGCCTCTTGTTGCTGCTTTGACTTTTCATCAATTTTTCGAAGGCATGGGCCTTGGTGGA     549
XM_006353661.2-StZIP5-like     AAGCCACTGGTTGCTGCTTTGACTTTTCATCAGTTTTTTGAAGGCATGGGACTTGGTGGA     714
                               * *** ** ****  ***************** ***** ** ** ***** *********

XM_016593570.1-NtZIP5B         TGCATTGCTCAGGCAAAATTCAAGACTCGGGCAGTGGCAATAATGGCTTTATTTTTCTCA     774
XM_009770855.1-NsZIP5-like     TGCATTGCTCAGGCAAAATTCAAGACTCGGGCAGTGGCAATAATGGCTTTATTTTTCTCA     774
XM_019381545.1-NaZIP5-like     TGCATTGCTCAGGCAAAATTCAAGACTCGGGCAGTGGCAATAATGGCTTTATTTTTCTCT     774
NM_001325745.1-NtZIP5A         TGTATTGCTCAGGCAAAATTCAAGACTCGTGCAGTGGCAATAATGGCTTTATTTTTCTCT     762
AB505626.1-NtZIP1              TGTATTGCTCAGGCAAAATTCAAGACTCGTGCAGTGGCAATAATGGCTTTATTTTTCTCT     762
XM_009612040.2-NtomZIP5        TGCATTGCTCAGGCAAAATTTAAGACTCGTGCAGTGGCAATAATGACTTTATTTTTCTCT     768
XM_009776848.1-NsZIP5-like     TGCATTGCTCAGGCAAAATTCAAGAATCGTGCAGTGGTAATAATGACTTTATTTTTCTCT     771
XM_019387083.1-NaZIP5-like     TGCATTGCTCAGGTA---------------------------------------------     729
XM_016594002.1-NtZIP5-like     TGCATTGCTCAGGCAAAATTTAAGACTCGTGCAGTGGCAATAATGACTTTATTTTTCTCT     609
XM_006353661.2-StZIP5-like     TGCATTGCTCAGGCAAAATTGAAGTCTCGTGCAATTGCAATAATGGCTTTATTTTTCTCC     774
                               ** ********** *
```

TM VI

```
XM_016593570.1-NtZIP5B         CTTACAACTCCAGTGGGTATTGCAATTGGACTAGGAATAACAAATGTTTACGATGAAAAC     834
XM_009770855.1-NsZIP5-like     CTTACAACTCCAGTGGGTATTGCAATTGGACTAGGAATAACAAATGTTTACGATGAAAAC     834
XM_019381545.1-NaZIP5-like     CTTACAACTCCAGTGGGAATTGCAATTGGACTAGGAATAACAAATGTTTACGATGAAAAC     834
NM_001325745.1-NtZIP5A         CTTACAACTCCAGTCGGTATTGCAATTGGATTAGGAATAACAAATGTTTACGATGAAAAC     822
AB505626.1-NtZIP1              CTTACAACTCCAGTCGGTATTGCAATTGGATTAGGAATAACAAATGTTTACGATGAAAAC     822
XM_009612040.2-NtomZIP5        CTCACAACTCCAATCGGAATTGGAATTGGATTAGGAATAACAAATTCTTACGATGAAAAC     828
XM_009776848.1-NsZIP5-like     CTCACAACTCCAATAGGAATTGGAATTGGACTAGGAATAACAAATTCTTACGATGAAAAC     831
XM_019387083.1-NaZIP5-like     ------------------------------------------------------------     729
XM_016594002.1-NtZIP5-like     CTCACAACTCCAATAGGAATTGGAATTGGATTAGGAATAACAAATTCTTACGATGAAAAC     669
XM_006353661.2-StZIP5-like     CTCACAACTCCAGTTGGAATTGCAGTTGGACTAGCAATAACAAATGTTTACGATGAGAAT     834

XM_016593570.1-NtZIP5B         AGTCCAACGGCTCTCATTGTGGAAGGAGTATTTAATTCAGCATCAGCTGGTATCTTGATT     894
XM_009770855.1-NsZIP5-like     AGTCCAACGGCTCTCATTGTGGAAGGAGTATTTAATTCAGCATCAGCTGGTATCTTGATT     894
XM_019381545.1-NaZIP5-like     AGTCCGACGGCTCTCATTGTAGAAGGAGTATTTAATTCAGCATCAGCTGGTATCTTGATT     894
NM_001325745.1-NtZIP5A         AGTCCAACGGCTCTTATTGTGGAAGGAGTATTTAATTCAGCATCAGCTGGTATCTTGATT     882
AB505626.1-NtZIP1              AGTCCAACGGCTCTTATTGTGGAAGGAGTATTTAATTCAGCATCAGCTGGTATCTTGATT     882
XM_009612040.2-NtomZIP5        AGTCCAACGGCTCTTATTGTGGAAGGAGTATTTAATTCAGCATCAGCTGGTATCTTGATT     888
XM_009776848.1-NsZIP5-like     AGTCCAACAGCTCTTATTGTGGAAGGAGTATTTAATTCAGCATCAGCTGGTATCTTGATT     891
XM_019387083.1-NaZIP5-like     ------------------------------------------------------------     729
XM_016594002.1-NtZIP5-like     AGTCCAACGGCTCTTATTGTGGAAGGAGTATTTAATTCAGCATCAGCTGGTATCTTGATT     729
XM_006353661.2-StZIP5-like     AGTCCAACGGCTCTTATCGTGGAAGGAATATTTAATTCAGCTTCAGCTGGCATCTTGATT     894
```

TM VII

```
XM_016593570.1-NtZIP5B         TATATGGCATTAGTTGATTTTTTGGCTGCTGATTTTATGCATCCAAGAATGCAAGGCAAT     954
XM_009770855.1-NsZIP5-like     TATATGGCATTAGTTGATTTTTTGGCTGCTGATTTTATGCATCCAAGAATGCAAGGCAAT     954
XM_019381545.1-NaZIP5-like     TATATGGCATTAGTTGATTTTTTGGCTGCTGATTTTATGCATCCAAGAATGCAAGGCAAT     954
NM_001325745.1-NtZIP5A         TATATGGCACTTGTTGATTTTTTAGCTGCTGATTTTATGCATCCAAGAATGCAAGGCAAT     942
AB505626.1-NtZIP1              TATATGGCACTTGTTGATTTTTTAGCTGCTGATTTTATGCATCCAAGAATGCAAGGCAAT     942
XM_009612040.2-NtomZIP5        TATATGGCATTAGTTGATTTTTTAGCTGCCGATTTTATGCATCCAAGAATGCAAGGCAAT     948
XM_009776848.1-NsZIP5-like     TATATGGCACTTGTCGATTTTTTAGCTGCTGATTTTATGCATCCAAGAATGCAAGGCAAT     951
XM_019387083.1-NaZIP5-like     ------------------------------------------------------------     729
XM_016594002.1-NtZIP5-like     TATATGGCATTAGTTGATTTTTTAGCTGCCGATTTTATGCATCCAAGAATGCAAGGCAAT     789
XM_006353661.2-StZIP5-like     TATATGGCGCTCGTTGACTTTTTAGCTGCCGATTTTATGCATCCAAGAATGCAAGGCAAT     954
```

TM VIII

```
XM_016593570.1-NtZIP5B         GGAAAACTTCAATTAGGTGCCAATGTTTCACTTCTTCTTGGTGCTGGACTCATGGCTCTC     1014
XM_009770855.1-NsZIP5-like     GGAAAACTTCAATTAGGTGCCAATGTTTCACTTCTTCTTGGTGCTGGACTCATGGCTCTC     1014
XM_019381545.1-NaZIP5-like     GGAAAACTTCAATTAGGTGCCAATATTTCACTTCTTCTTGGTGCTGGACTCATGGCTCTC     1014
NM_001325745.1-NtZIP5A         GGAAAGCTTCAATTAGGGGCAAATATTTCACTTCTTCTCGGTGCTGGACTCATGGCTCTC     1002
AB505626.1-NtZIP1              GGAAAGCTTCAATTAGGGGCAAATATTTCACTTCTTCTCGGTGCTGGACTCATGGCTCTC     1002
XM_009612040.2-NtomZIP5        GGAAAACTTCAATTAGGAGCCAATATTTCACTTCTTCTTGGTGCTGGACTTATGTCTCTC     1008
XM_009776848.1-NsZIP5-like     GGAAAACTTCAATTAGGAGCCAATATTTCACTTCTTCTTGGTGCTGGACTTATGTCTCTC     1011
XM_019387083.1-NaZIP5-like     ------------------------------------------------------------     729
XM_016594002.1-NtZIP5-like     GGAAAACTTCAATTAGGAGCCAATATTTCAGTTCTTCTTGGTGCTGGACTTATGTCTCTC     849
XM_006353661.2-StZIP5-like     GGAAAGCTTCAATTAGGGGCTAATGTTTCACTTCTTCTTGGCGCTGGTCTTATGTCTATG     1014
```

```
XM_016593570.1-NtZIP5B          ATAGCCAAATGGGCTTAA     1032
XM_009770855.1-NsZIP5-like      ATAGCCAAATGGGCTTAA     1032
XM_019381545.1-NaZIP5-like      ATAGCCAAATGGGCTTAA     1032
NM_001325745.1-NtZIP5A          ATAGCCAAATGGGCTTAA     1020
AB505626.1-NtZIP1               ATAGCCAAATGGGCTTAA     1020
XM_009612040.2-NtomZIP5         TTAGCCAAATGGGCTTAA     1026
XM_009776848.1-NsZIP5-like      CTAGCCAAATGGGCTTAA     1029
XM_019387083.1-NaZIP5-like      ------------------     729
XM_016594002.1-NtZIP5-like      TTAGCCAAATGGGCTTAA     867
XM_006353661.2-StZIP5-like      TTAGCCAAATGGGCTTAA     1032
```

(b) Comparison of amino acid sequences of NtZIP5A / B with NtZIP5 sequences identified in the NCBI data base;

The list of ZIP5 proteins identified as the best Hits are given in the Table below;

NtZIP5B sequence (XP_016449056.1) was used as a query.

Description	Max score	Total score	Query cover	E value	Ident	Protein accession	mRNA accession
PREDICTED: zinc transporter 5-like [*Nicotiana sylvestris*]	694	694	100%	0.0	100 %	XP_009769157.1	XM_009770855.1
PREDICTED: zinc transporter 5-like [*Nicotiana attenuata*]	681	681	100%	0.0	97%	XP_019237090.1	XM_019381545.1
zinc transporter 5-like precursor [*Nicotiana tabacum*] [in Sano et al., 2012 named NtZIP1] [in this study renamed to NtZIP5A]	672	672	100%	0.0	97%	NP_001312674.1 [previously BAH 66920.1]	NM_001325745.1
PREDICTED: zinc transporter 5-like [*Solanum tuberosum*]	619	619	100%	0.0	86%	XP_006353723.1	XM_006353661.2
PREDICTED: zinc transporter 5-like [*Nicotiana sylvestris*]	619	619	100%	0.0	89%	XP_009775150.1	XM_009776848.1
PREDICTED: zinc transporter 5 [*Nicotiana tomentosiformis*]	605	605	100%	0.0	88%	XP_009610335.1	XM_009612040.2
PREDICTED: zinc transporter 5-like [*Solanum pennellii*]	595	595	99%	0.0	84%	XP_015081742.1	XM_015226256.1
Zinc transporter 5 [*Capsicum annuum*]	589	589	100%	0.0	83%	PHT75648.1	* AYRZ02000007.1 43331107..43333849

Zinc transporter 5 [*Capsicum chinense*]	588	588	100%	0.0	83%	PHU11901.1	* MCIT02000007.1 161184166..161186921
PREDICTED: zinc transporter 5-like [*Solanum tuberosum*]	585	585	100%	0.0	85%	XP_006353722.1	XM_006353660.2

*no mRNA sequence

Alignments of amino acid sequences between NtZIP5B (used as a query) and ZIP5 proteins identified as the best hit sequences. Sequences were aligned using Clustal Omega https://www.ebi.ac.uk/Tools/msa/clustalo/. The prediction of membrane-spanning regions was performed using Phobius programme (http://phobius.sbc.su.se/), and indicated as lines above the sequences, and numbered TM I–VIII respectively. Sequences of the transmembrane domains (TM,) are marked with light grey; identical amino acids with dark grey; histidine in black. Dashes indicate gaps.

```
                                                                      TM I
NP_001312674.1-NtZIP5A        MTKLEKVIFWYILLLLPAIVLGECTCDSEDEE-RNKTEALKYKMVAIASILIASAIGVCI    59
XP_016449056.1-NtZIP5B        MTKLEKVVFWYILLLLPAIVLGECTCDSEDEE-RNKPEALKYKMVAIASILIASAIGVCI    59
XP_009769157.1-NsZIP5-like    MTKLEKVVFWYILLLLPAIVLGECTCDSEDEE-RNKPEALKYKMVAIASILIASAIGVCI    59
XP_019237090.-NaZIP5-like     MTKLEKVVFWYILLLLPAIVLGECTCESEDEE-RNKTEALKYKMVAIATILVASAIGVCI    59
XP_006353723.1-StZIP5-like    MAKMEKIVFWYILLLLPAIVLGECTCDSEDEE-RNKSEALKYKMAALASILVASAIGVCI    59
XP_009775150.1-NsZIP5-like    MEKLKNVIFWYILVLLPAIVLGECTCDSEDEE-RNKAEALKYKMVAVASILVASALGVCI    59
XP_009610335.1-NtomZIP5       MAKLEKVVFWYILVLLPAIVLGECTCDSDEEE-RNKTEALKYKMVAVASILVASALGVCI    59
XP_015081742.1-SpZIP5-like    ---MAKMIFWYILLILPAIVLGECTCDPEDED-RNKSEALKYKMAALASILVASAIGVCI    56
PHT75648.1-CaZIP5             MAKFVEVVFWYILSLLPAIVLGECTCDSDDDE-RNKNEALKYKMAAFASILVASTIGVCI    59
PHU11901.1-CcZIP5             MAKFVKVVFWYILSLLPAIVLGECTCDSDDDEERNKNEALKYKMAALASILFASTIGVCI    60
XP_006353722.1-StZIP5-like    MTKFEKMIFWYILLLFPAIVLSECTCDPEDED-RNKKEALKYKMAALASILVASAIGVCI    59
                                      *****   ***** ****       *** ******* * * ** ** ****

                                                    TM II
NP_001312674.1-NtZIP5A        PVLGKAIPALSPEKNFFFIIKAFAAGVILATGFIHVLPDAFESLTSPCLKENPWGNFPFS    119
XP_016449056.1-NtZIP5B        PVLGKAIPALSPEKNFFFIIKAFAAGVILATGFIHVLPDAFESLTSPCLKENPWGNFPFS    119
XP_009769157.1-NsZIP5-like    PVLGKAIPALSPEKNFFFIIKAFAAGVILATGFIHVLPDAFESLTSPCLKENPWGNFPFS    119
XP_019237090.-NaZIP5-like     PVLGKAIPALSPEKNFFFIIKAFAAGVILATGFIHVLPDAYESLTSPCLKENPWGNFPFS    119
XP_006353723.1-StZIP5-like    PVLGKVIPALSPERNFFFIIKAFAAGVILSTGFIHVLPDAFESLTSPCLKENPWGNFPFC    119
XP_009775150.1-NsZIP5-like    PVLGKAIPALSPEKNLFFIIKAFAAGVILSTGFIHVLPDAYESLTSPCLKENPWGDFPFS    119
XP_009610335.1-NtomZIP5       PVLGKAIPALSPEKNLFFIIKAFAAGVILATGFIHVLPDAYKSLTSPYLKENPWGDFPFS    119
XP_015081742.1-SpZIP5-like    PVLGKVIPALSPERNFFFIIKAFAAGVILSTGFIHVLPDAFESLTSPCLPEHPWGDFPFC    116
PHT75648.1-CaZIP5             PVLGKAVPALSPERNFFFVIKAFAAGVILSTGFIHVLPDAFESLTSPCLKENPWGKFPFS    119
PHU11901.1-CcZIP5             PVLGKAVPALSPERNFFFVIKAFAAGVILSTGFIHVLPDAFESLTSPCLKENPWGKFPFS    120
XP_006353722.1-StZIP5-like    PVLGKAIPALSPERNFFFIIKAFAAGVILSTGFIHVLPDAFESLTSPCLAEHPWGDFPFS    119
                              *****  ****** * ** ********** **********  ***** * * *** ***

                                   TM III
NP_001312674.1-NtZIP5A        GFIAMVSAMGTLMVDTYATSYFSNKNDTKNGLVAQSGDEGGAI-HV---HSHGSASLMGD    175
XP_016449056.1-NtZIP5B        GFIAMVSAMGTLMVDTYATSYFSNKNDTKNGLVAQSGDEGGAIHVHSHGHAHGSSSLLGD    179
XP_009769157.1-NsZIP5-like    GFIAMVSAMGTLMVDTYATSYFSNKNDTKNGLVAQSGDEGGAIHVHSHGHAHGSSSLLGD    179
XP_019237090.-NaZIP5-like     GFIAMVSAMGTLMVDTYATSYFRNKNDTKNGLVAQSGDEGGAIHVHSHGHAHGASSMMGD    179
XP_006353723.1-StZIP5-like    GFVAMVSAMATLMVDTYATSYYNKKNLKNGVVTTEGGDEERVIHTHSHAHAHGSTTMMAD    179
XP_009775150.1-NsZIP5-like    GFIAMVSAMATLMVDTYATSYYKNKS-VKNGVVAQSGDEEGVIHVHSHGHAHGSTSMIGD    178
XP_009610335.1-NtomZIP5       GFIAMVSAMATLMVDTYATSYYKNKSAKN-GVVAQSGDEELVN-VHSHGHVHGSTSMIGD    177
XP_015081742.1-SpZIP5-like    GFVAMVSAMATLMVDTYATSYYNKKNLKNGVVGTEVGDEGGVVHTHSHAHAHGSTTMMVD    176
PHT75648.1-CaZIP5             GFVAMVAAMATLMVDAYATSYYNKKFIESGVMVQ---SGDGVVHANSHGHVHGSASMMAN    176
PHU11901.1-CcZIP5             GFVAMVAAMSTLMIDTYATSYYNKKFINSGVVVQ---SGDGVVHANSHGHVHGSASIMAN    177
XP_006353722.1-StZIP5-like    GFIAMVSALATLMVDTYATSYYSKKNLKNGVVATEAGYEQGVVHAHSHGHVHGLSD----    175
                              ** *** *  *** * *****   *                        * **

                                          TM IV                    TM V
NP_001312674.1-NtZIP5A        SSSELLRYRVVSQVLEMGIIVHSVIIGIALGASESPKTIRPLVAALTFHQFFEGMGLGGC    235
XP_016449056.1-NtZIP5B        SSSELLRYRVVSQVLEMGIIVHSVIIGIALGASESPKTIRPLVGALTFHQFFEGMGLGGC    239
XP_009769157.1-NsZIP5-like    SSSELLRYRVVSQVLEMGIIVHSVIIGIALGASESPKTIRPLVGALTFHQFFEGMGLGGC    239
XP_019237090.-NaZIP5-like     SSSELLRYRVVSQVLELGIIVHSVIIGIALGASESPKTIRPLVGALTFHQFFEGMGLGGC    239
XP_006353723.1-StZIP5-like    SNSELLRYRVIAQVLELGIIVHSVIIGIALGASESPKTIKPLVAALTFHQFFEGMGLGGC    239
```

```
XP_009775150.1-NsZIP5-like      SDSELLRYRVISQVLELGIIVHSVIIGIALGASESPKTIRPLVGALTFHQFFEGMGLGGC      238
XP_009610335.1-NtomZIP5         SDSELLRYRVISQVLEVGIIVHSVIIGIALGASESPKTIRPLVAALTFHQFFEGMGLGGC      237
XP_015081742.1-SpZIP5-like      SNSELLRYRVISQVLELGIIVHSVIIGIALGASESPKTIKPLVAALTFHQFFEGMGLGGC      236
PHT75648.1-CaZIP5               PESELLRYRVISQVLELGIIVHSVIIGIALGASQSPKTIKPLVAALTFHQFFEGMGLGGS      236
PHU11901.1-CcZIP5               PESELLRYRVISQVLELGIIVHSVIIGIALGASQSPKTIKPLVAALTFHQFFEGMGLGGS      237
XP_006353722.1-StZIP5-like      SESELLRYRVISQVLELGIIVHSVIIGIALGASESPKTIKPLVAALTFHQFFEGLGLGGC      235
                                  ********  **** **************** ***** *** ********** ****

                                          TM VI                                   TM VIII
NP_001312674.1-NtZIP5A          IAQAKFKTRAVAIMALFFSLTTPVGIAIGLGITNVYDENSPTALIVEGVFNSASAGILIY      295
XP_016449056.1-NtZIP5B          IAQAKFKTRAVAIMALFFSLTTPVGIAIGLGITNVYDENSPTALIVEGVFNSASAGILIY      299
XP_009769157.1-NsZIP5-like      IAQAKFKTRAVAIMALFFSLTTPVGIAIGLGITNVYDENSPTALIVEGVFNSASAGILIY      299
XP_019237090.-NaZIP5-like       IAQAKFKTRAVAIMALFFSLTTPVGIAIGLGITNVYDENSPTALIVEGVFNSASAGILIY      299
XP_006353723.1-StZIP5-like      IAQAKLKSRAIAIMALFFSLTTPVGIAVGLAITNVYDENSPTALIVEGIFNSASAGILIY      299
XP_009775150.1-NsZIP5-like      IAQAKFKNRAVVIMTLFFSLTTPIGIGIGLGITNSYDENSPTALIVEGVFNSASAGILIY      298
XP_009610335.1-NtomZIP5         IAQAKFKTRAVAIMTLFFSLTTPIGIGIGLGITNSYDENSPTALIVEGVFNSASAGILIY      297
XP_015081742.1-SpZIP5-like      IAQAKLKCREITIMALFFSLTTPVGIAIGLGITNVYDENSPTALIVQGIFDSASAGILIY      296
PHT75648.1-CaZIP5               IAQAKLKSRAIAIMALFFSLTTPIGIAIGLGITNDYDENSPTALIVEGIFNSASAGILIY      296
PHU11901.1-CcZIP5               IAQAKLKSRAIAIMALFFSLTTPIGIAIGLGITNDYDENSPTALIVEGIFNSASAGILIY      297
XP_006353722.1-StZIP5-like      IAQAKLKTRTIAIMALFFSLTTPIGIGIGLGITNVYDENSPTALIVEGVFNSASAGILIY      295
                                ***** * *   ** ******** **  ** *** *********** * * *********

                                                         TM VIII
NP_001312674.1-NtZIP5A          MALVDFLAADFMHPRMQGNGKLQLGANISLLLGAGLMALIAKWA 339
XP_016449056.1-NtZIP5B          MALVDFLAADFMHPRMQGNGKLQLGANVSLLLGAGLMALIAKWA 343
XP_009769157.1-NsZIP5-like      MALVDFLAADFMHPRMQGNGKLQLGANVSLLLGAGLMALIAKWA 343
XP_019237090.-NaZIP5-like       MALVDFLAADFMHPRMQGNGKLQLGANISLLLGAGLMALIAKWA 343
XP_006353723.1-StZIP5-like      MALVDFLAADFMHPRMQGNGKLQLGANVSLLLGAGLMSMLAKWA 343
XP_009775150.1-NsZIP5-like      MALVDFLAADFMHPRMQGNGKLQLGANISLLLGAGLMSLLAKWA 342
XP_009610335.1-NtomZIP5         MALVDFLAADFMHPRMQGNGKLQLGANISLLLGAGLMSLLAKWA 341
XP_015081742.1-SpZIP5-like      MALVDFLAADFMNPRMQGNGKLQLGANVSLLLGAGLMSMLAKWA 340
PHT75648.1-CaZIP5               MALVDFLAADFMHPRMQGNGKLQLGANISLLLGAGLMSLLAKWA 340
PHU11901.1-CcZIP5               MALVDFLAADFMHPRMQGNGKLQLGANISLLLGAGLMSLLAKWA 341
XP_006353722.1-StZIP5-like      MALVDFLAADFMHPRMQGNGKLQLGANISLLLGAGLMSLLAKWA 339
                                ************ ************** *********   ****
```

(c) Comparison of cDNA nucleotide sequences of *NtZIP5A* and *NtZIP5B*.

Nucleotide alignment of ***NtZIP5A*** (NM_001325745.1) and ***NtZIP5B*** (XM_016593570.1) using ClustalW.

The primer pairs used for cloning and ZIP5B-ORF-START / ZIP5B-STOP (with the STOP codon) are listed in the **Additional file 3**.

In grey - nucleotides different between sequences

Underlined in green – sequence identical with the primer sequence ZIP4B-ORF-START used for cloning of the ORF of *NtZIP5B*.

Underlined in orange – sequence complementary to primer sequence ZIP4B-ORF-STOP used for cloning of *NtZIP5B*.

Underlined in blue double line – sequence identical or complementary to the primer sequences used for *NtZIP5A* expression analysis by RT-qPCR.

Underlined in red double line – sequence identical or complementary to the primer sequences used for *NtZIP5B* expression analysis by RT-qPCR.

In red – START codon

In blue – STOP codon

```
CLUSTAL O(1.2.4) multiple sequence alignment

NM_001325745.1/NtZIP5A      ATTGGAATTTGTAACATTTCAGATTCTAAGTGATTCTTTTCTCTCAATCGTACTTCCAAA      60
XM_016593570.1/NtZIP5B      ------------------------------------------------------------      0


NM_001325745.1/NtZIP5A      ACCCTTTTTCATTTTCTATACCTAGAGTCATGACAAAGTTAGAAAAAGTTATTTTTTGGT      120
XM_016593570.1/NtZIP5B      -----------------------------ATGACAAAGTTAGAAAAAGTAGTTTTTTGGT      31
                                                         ********************:.*********

NM_001325745.1/NtZIP5A      ACATTCTCTTGCTTCTTCCTGCTATAGTATTAGGCGAATGTACTTGTGATTCTGAGGATG      180
XM_016593570.1/NtZIP5B      ACATTCTCTTGCTTCTTCCTGCTATAGTATTAGGAGAATGTACTTGTGATTCTGAGGATG      91
                            **********************************.*************************

NM_001325745.1/NtZIP5A      AAGAAAGAAACAAAACTGAAGCACTGAAGTATAAAATGGTAGCAATCGCTTCCATTCTGA      240
XM_016593570.1/NtZIP5B      AAGAAAGAAACAAACCTGAAGCACTGAAATATAAAATGGTAGCAATCGCTTCCATTTTGA      151
                            **************.*************.*************************** ***

NM_001325745.1/NtZIP5A      TTGCCAGTGCAATTGGGGTTTGTATTCCTGTACTTGGAAAAGCAATTCCAGCTTTAAGCC      300
XM_016593570.1/NtZIP5B      TTGCCAGTGCAATTGGGGTTTGTATTCCTGTACTAGGAAAAGCAATTCCAGCTTTGAGCC      211
                            **********************************:********************.****

NM_001325745.1/NtZIP5A      CAGAGAAGAATTTCTTCTTCATAATCAAAGCTTTTGCTGCTGGTGTGATCCTCGCGACAG      360
XM_016593570.1/NtZIP5B      CAGAGAAGAATTTCTTCTTCATAATCAAAGCTTTTGCTGCTGGTGTGATCCTCGCGACAG      271
                            ************************************************************

NM_001325745.1/NtZIP5A      GGTTTATACATGTACTTCCTGATGCTTTTGAAAGCTTAACATCGCCATGTTTGAAAGAGA      420
XM_016593570.1/NtZIP5B      GGTTTATACATGTACTCCCTGATGCATTTGAAAGCTTAACATCGCCATGTTTGAAAGAGA      331
                            **************** ********:**********************************

NM_001325745.1/NtZIP5A      ATCCGTGGGGAAATTTTCCTTTTAGTGGATTTATTGCAATGGTTTCTGCAATGGGAACTC      480
XM_016593570.1/NtZIP5B      ATCCGTGGGGAAATTTTCCATTCAGTGGATTTATCGCAATGGTTTCTGCAATGGGAACTC      391
                            *******************:** *********** *************************

NM_001325745.1/NtZIP5A      TTATGGTGGATACTTATGCAACTTCATATTTCAGTAACAAAAATGACACGAAAAATGGAT      540
XM_016593570.1/NtZIP5B      TAATGGTGGACACTTATGCAACTTCATATTTCAGTAACAAAAATGATACGAAAAATGGAT      451
                            *:******** *********************************** *************

NM_001325745.1/NtZIP5A      TGGTGGCTCAGTCTGGAGATGAAGGAGGAGCTATTCATGTTCATTCA------------C      588
XM_016593570.1/NtZIP5B      TGGTGGCTCAGTCTGGAGATGAAGGAGGAGCTATACATGTTCATTCACATGGCCATGCAC      511
                            **********************************:************            *
```

```
NM_001325745.1/NtZIP5A      ATGGTTCAGCATCACTGATGGGTGATTCCAGTTCTGAGCTCCTTCGTTATCGTGTTGTAT      648
XM_016593570.1/NtZIP5B      ATGGTTCATCATCACTGCTGGGTGATTCTAGTTCCGAGCTCCTTCGTTATCGTGTTGTAT      571
                            ******** ********.********** ***** *************************

NM_001325745.1/NtZIP5A      CTCAGGTATTGGAAATGGGGATAATAGTGCATTCTGTGATAATAGGAATAGCTTTGGGTG      708
XM_016593570.1/NtZIP5B      CTCAGGTATTGGAAATGGGGATAATAGTGCATTCTGTGATAATAGGAATAGCTTTGGGTG      631
                            ************************************************************

NM_001325745.1/NtZIP5A      CTTCTGAAAGTCCCAAAACCATAAGGCCTCTTGTTGCTGCTTTGACTTTTCATCAATTTT      768
XM_016593570.1/NtZIP5B      CTTCTGAAAGTCCCAAAACCATAAGGCCTCTTGTTGGTGCTTTGACTTTTCATCAATTTT      691
                            ************************************ ***********************

NM_001325745.1/NtZIP5A      TCGAAGGCATGGGACTTGGTGGATGTATTGCTCAGGCAAAATTCAAGACTCGTGCAGTGG      828
XM_016593570.1/NtZIP5B      TCGAAGGCATGGGACTTGGTGGATGCATTGCTCAGGCAAAATTCAAGACTCGGGCAGTGG      751
                            ************************* ************************** *******

NM_001325745.1/NtZIP5A      CAATAATGGCTTTATTTTTCTCTCTTACAACTCCAGTCGGTATTGCAATTGGATTAGGAA      888
XM_016593570.1/NtZIP5B      CAATAATGGCTTTATTTTTCTCACTTACAACTCCAGTGGGTATTGCAATTGGACTAGGAA      811
                            **********************:************** *************** ******

NM_001325745.1/NtZIP5A      TAACAAATGTTTACGATGAAAACAGTCCAACGGCTCTTATTGTGGAAGGAGTATTTAATT      948
XM_016593570.1/NtZIP5B      TAACAAATGTTTACGATGAAAACAGTCCAACGGCTCTCATTGTGGAAGGAGTATTTAATT      871
                            ************************************* **********************

NM_001325745.1/NtZIP5A      CAGCATCAGCTGGTATCTTGATTTATATGGCACTTGTTGATTTTTTAGCTGCTGATTTTA      1008
XM_016593570.1/NtZIP5B      CAGCATCAGCTGGTATCTTGATTTATATGGCATTAGTTGATTTTTTGGCTGCTGATTTTA      931
                            ******************************** *:***********.*************

NM_001325745.1/NtZIP5A      TGCATCCAAGAATGCAAGGCAATGGAAAGCTTCAATTAGGGGCAAATATTTCACTTCTTC      1068
XM_016593570.1/NtZIP5B      TGCATCCAAGAATGCAAGGCAATGGAAAACTTCAATTAGGTGCCAATGTTTCACTTCTTC      991
                            ****************************.*********** **.***.************

NM_001325745.1/NtZIP5A      TCGGTGCTGGACTCATGGCTCTCATAGCCAAATGGGCTTAAT-TGATTAACATTGATTAT      1127
XM_016593570.1/NtZIP5B      TTGGTGCTGGACTCATGGCTCTCATAGCCAAATGGGCTTAATTTGATCAACACTGATTAT      1051
                            * **************************************** **** **** *******

NM_001325745.1/NtZIP5A      TATCAAATTCACATTATTTGTCTTTTTTT-----TATTTTTTCTGTCTTATCTCTTGACA      1182
XM_016593570.1/NtZIP5B      TATCAATTTCACATTATTTGTCTTTTTCTTTTAAATTTTTTTCTGTCTTATCTCTTGACA      1111
                            ******:******************** *     ::************************

NM_001325745.1/NtZIP5A      GAGTCTTAAACAGAATGTGACTGTAATTTTTTTGAATTTGCATTTCCCCATGATTTATTT      1242
XM_016593570.1/NtZIP5B      GAGTGTTATACAGGATGTGACTGTAATTTTTTTGATTTTGC-------------------      1152
                            **** ***:****.*********************:*****

NM_001325745.1/NtZIP5A      CATCATATTGATTTGTTTT                                               1261
XM_016593570.1/NtZIP5B      -------------------                                               1152
```

(d) Specificity of primers used for expression analysis of *NtZIP4A* and *NtZIP4B*.

The primer pairs used for expression analysis of NtZIP5A and NtZIP5B are listed in the **Additional file 3**.

(d1) As a template plasmid pRACE with the partial sequence of *NtZIP5A*, 659bp (137bp 5'UTR+ 522bp from ATG) included sequence used for *NtZIP5A* expression analysis.

(d2) As a template plasmid pENTR-NtZIP5B-STOP with the full sequence of the ORF of *NtZIP5B* was used.

(d1) – NtZIP5A

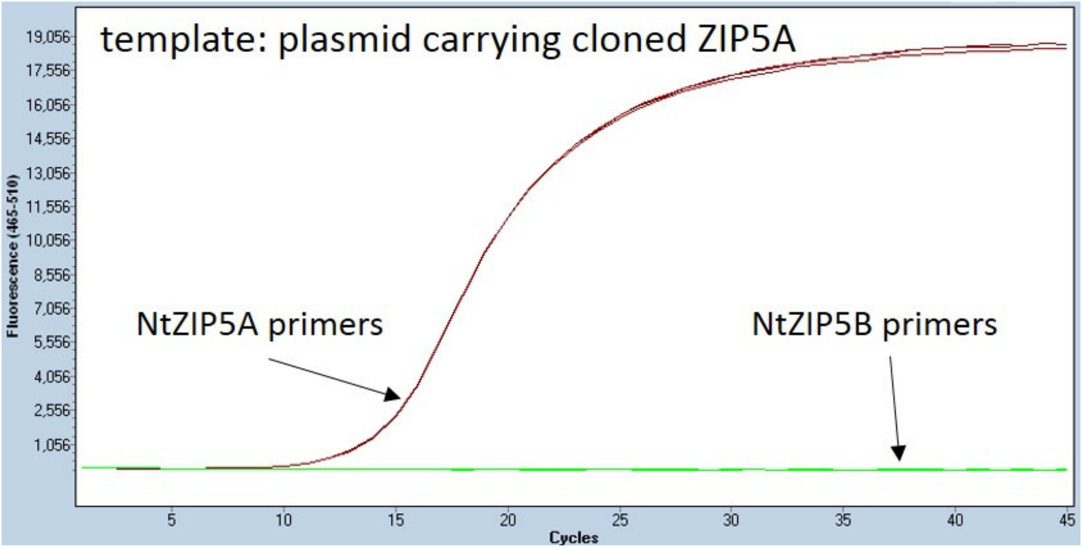

(d2) – NtZIP5B

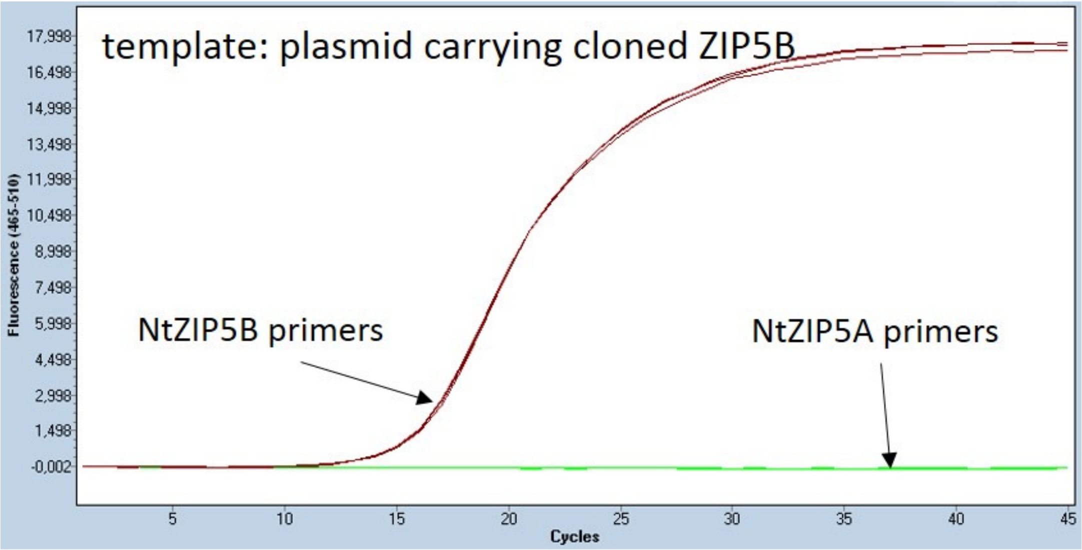

(e) Information on nucleotide and amino acid sequences of *NtZIP5A* and *NtZIP5B*, and *NtZIP5* and *NtZIP1* genes from selected plant species (based on data from the phylogenetic tree – Fig. 8).

Gene	Locus taq/ gen symbol #	Nucleotide Accession No	Protein Accession No	Lenght of mRNA (bp)	ORF (bp)	No. of exons	Protein length (aa)	Length of variable domain between TM III-IV (aa)	whole genome shotgun sequence#/ chromosome
NtZIP5 A	LOC107803903	NM_001325745.1	NP_001312674.1	1261	1017	3	339	51	AYMY01154470
NtZIP5 B	LOC107774100	XM_016593570.1	XP_016449056.1	1152	1029	3	343	55	AYMY01204208
CaZIP5	T459_19170		PHT75648.1		1020	3	340	52	7
MtZIP5	MTR_3g081580	XM_013605712.2	XP_013461166.1	1753	1074	3	358	67	3
MnZIP5	L484_009317	XM_010094823.2	XP_010093125.1	1546	1074	3	358	61	3
HvZIP5	LOC100301578	FJ208992.1	ACN93833.1	1053	1050		350	54	
OsZIP5	OSNPB_050472700	XM_015782024.2	XP_015637510.1	4174	1059	3	353	55	5
AtZIP5	AT1G05300	NM_100409.5	NP_172022.1	1554	1080	3	360	58	1
CsZIP5	LOC104763168	XM_010486577.2	XP_010484879.1	1412	1065	3	355	54	3
AtZIP1	AT3G12750	NM_112111.3	NP_187881.1	1412	1065	2	355	56	3
CsZIP1	LOC104776954	XM_010501137.2	XP_010499439.1	1254	1071	2	357	56	1
VvZIP1	VIT_00036734001	XM_002264567.4	XP_002264603.2	1263	1080	3	360	55	19

# - gene symbol is given if the sequence of the whole chromosome is not available or the locus tag is not given

References:

Barabasz A, Klimecka M, Kendziorek M, Weremczuk A, Ruszczyńska A, Bulska E, Antosiewicz DM. 2016. The ratio of Zn to Cd supply as a determinant of metal-homeostasis gene expression in tobacco and its modulation by overexpressing the metal exporter AtHMA4. *Journal of Experimental Botany*. **67**, 6201-6214

Sano T, Yoshihara T, Handa H, Sato MH, Nagata N, Hasezawa S. 2012. Metal Ion Homeostasis Mediated by NRAMP Transporters in Plant Cells - Focused on Increased Resistance to Iron and Cadmium Ion, Crosstalk and Integration of Membrane Trafficking Pathways, Dr.Roberto Weigert (Ed.), ISBN: 978-953-51-0515-2. Rijeka, Shanghai: INTECH, 214–228.
