# Supplementary material for: Zn/Cd status-dependent accumulation of Zn and Cd in root parts in tobacco is accompanied by specific expression of ZIP genes
Source: BMC Plant Biol. 2020 Jan 22;20:37. doi: 10.1186/s12870-020-2255-3 (PMC6977228; doi:10.1186/s12870-020-2255-3)
Supplement: Supplementary file 3 — Additional file 3 Sequence identity between the NtZIP5A, NtZIP5B and chosen ZIP5 and ZIP1 nucleotide sequences (a) and predicted proteins (b) from selected species [file 12870_2020_2255_MOESM3_ESM.pdf]

Additional file 3: Sequence identity between the NtZIP5A, NtZIP5B and ZIP1, ZIP5 nucleotide sequences- ORF (Open Reading Frame) (a) and predicted proteins (b) from selected species (sequences were chosen based on phylogenetic tree given in Figure 1), using Clustal Omega https://www.ebi.ac.uk/Tools/msa/clustalo/

(a)

	NtZIP5A	NtZIP5B	CaZIP5	MtZIP5	MnZIP5	HvZIP5	OsZIP5	AtZIP5	ZIP1-like	AtZIP1
**NtZIP5B**	96.46									
**CaZIP5**	*	*								
**MtZIP5**	70.40	69.68	*							
**MnZIP5**	66.96	67.25	*	65.45						
**HvZIP5**	55.52	55.36	*	54.30	59.77					
**OsZIP5**	52.38	51.75	*	51.92	58.67	67.34				
**AtZIP5**	61.14	61.01	*	58.92	59.55	55.43	54.10			
**ZIP1-like**	61.52	60.80	*	60.34	60.62	52.50	50.24	55.61		
**AtZIP1**	58.81	58.31	*	57.38	58.26	53.37	52.10	54.60	55.92	
**VvZIP1**	63.98	63.42	*	61.92	66.29	57.58	55.52	57.51	62.06	61.23

* nucleotide sequence not available

(b)

	NtZIP5A	NtZIP5B	CaZIP5	MtZIP5	MnZIP5	HvZIP5	OsZIP5	AtZIP5	ZIP1-like	AtZIP1
**NtZIP5B**	98.23									
**CaZIP5**	83.63	82.65								
**MtZIP5**	69.62	69.68	67.35							
**MnZIP5**	70.71	70.76	70.50	67.13						
**HvZIP5**	55.90	56.13	56.00	58.53	57.69					
**OsZIP5**	54.13	55.29	53.33	55.65	54.94	60.81				
**AtZIP5**	59.82	59.41	58.75	54.24	58.64	48.84	47.56			
**NtZIP1-like**	59.92	55.56	55.46	56.09	56.66	50.74	47.52	48.30		
**AtZIP1**	54.08	54.03	55.42	53.74	55.62	51.06	49.55	49.99	54.47	
**VvZIP1**	62.54	62.10	62.35	59.44	64.23	57.69	51.31	53.12	59.10	60.17
