# Supplementary material for: Zn/Cd status-dependent accumulation of Zn and Cd in root parts in tobacco is accompanied by specific expression of ZIP genes
Source: BMC Plant Biol. 2020 Jan 22;20:37. doi: 10.1186/s12870-020-2255-3 (PMC6977228; doi:10.1186/s12870-020-2255-3)
Supplement: Supplementary file 4 — Additional file 4. Supplementary information on cloning of NtZIP5B promoter (primers, promoter sequences) [file 12870_2020_2255_MOESM4_ESM.pdf]

Additional file 4:

Content:

a. Primers used for cloning of *NtZIP5B* promoter

b. Identification of *cis*-acting elements including 5'UTR within the promoter sequences of *NtZIP5A* (from the contig AWOK01321153.1 position 22508…20712 orientation plus/minus – without ATG), **and *NtZIP5B*** (from the contig AWOJ01230056.1 position 1…1726 – without ATG).

c. Comparison of the promoter sequences including 5'UTR of *NtZIP5A* from four tobacco cultivars: K326 Nitab4.5 (NCAA01000170.1 position 898333…896770, orientation plus/minus), **NT90** (AYMY01154470.1 position 1…1604), **Basma Xanthi** (AWOK01321153.1 position 22508…20712, orientation plus/minus) and **K326** (AWOJ01501266.1 position 7785…6182, orientation plus/minus).

d. Comparison of the promoter sequences including 5'UTR of *NtZIP5B* from four tobacco cultivars: K326 Nitab4.5 (NCAA01006169.1 position 29448…27794, orientation plus/minus), **NT90** (AYMY01204208.1 position 8544…10543), **Basma Xanthi** (AWOK01242628.1 position 1…1607) and **K326** (AWOJ01230056.1 position 1…1726).

a. Primers used for cloning of *NtZIP5B* promoter

Primer Forward	promZIP5B-F	CACCGGTCATATTTTCAGCCCAAAC
Primer Reverse	promZIP5B-R	CATGACTCTAGGTTTAGAAAACC

CACC – sequence added to clone the insert into the pENTR plasmid in proper orientation.

Cloned promoter sequence of *NtZIP5B* + 5'UTR + START codon (1726 bp without ATG)**:**

In red - START codon.

In red underlined - sequence of the forward primer and the sequence complementary to the reverse primer.

```
GGTCATATTTTCAGCCCAAACGGCTAGATTTTAAATATGGCCGTTGGAGAATTTTAATTTTTTTAAAAAAATAGC
CGTTGGGCCCGCCAGGCCCGTCTGACGGTCCCGGGCTAAACGGTCCCGGGCTCGTGGGCTAATACTACAAGACCG
GCCCGTCACGGGCTTCTCAGGACCACCAGGACAGGCCCACCAGGCCCGTTAGGACCGCGTGCCCGGGCCGGTCTG
GTTCAGGCCCGGCCCACCAAGCAGCCTTAATTACACCTATAAGAAAATTTGCGGGATTTCACGGAAAATATATCG
CTATAAACACGTCTAACTAATATGGATAAAAAGAAACTAAAATAAGACGAACACCATTTATAACATATAAATTGT
GTCAACAAGCACGTGGAACGAAACTAAAGTAAGGGATCAAATGTATGTCACCAACACTCCAACGATATATAAAAA
AAATACATCATCACGGCTAACTCTGTTTTCCATTAATTTGAACTTCATTCGCTTGAGCTTTAAATATGATCATTC
ATTAAATGTGGTATCCCTTGCTGAAAGTCCTAACCTTTAAACCTGAAAGAAATTAAGAAAAATATTAAGAACTCT
GTCTTTTTAGTATATCTTATCGGTTTATCGATGAACTGATAATCGAAGAGGACAAAATCAAAATCGAAATCGATT
ACCCGATAAGAAAAATTTCGAAATCGAAATCGAAATCGATAAACCGATAACAAATAATCGATTCGATTTATCGAT
AAACCGATTCATGACTTTATTTACATTGGATTATTACGTTAAACTGATGACCTGCTATCTCAATAGAGGGTGAGA
GGATTCAAAACCTTCTCGTTATGTACTTTTAATTGATAAATTAAGGAAGAATGAATAGAGGGGGTTAAGATTCAA
AACCTTCTCGTTATGTACTTTTAATTGACAAATTAAAGGAGAATGACAAGAACGTTTTAGCACGTATAGAATTAG
TTAATAAAATTATAAGGAATTAAACAAACAAATTTAATCAGGAAATAATTAATAGTAAAGAATAAATTTGTTTAG
AATCCACAAAAATTTTAAAAAAATAAAATAAGCCAGCTAAGTAAGAGCAATTTACGAGGAGGAAGTATATCCCGT
TAATCTATTCAGCTAGAATATATGTCGACATTTTTTCGACACATAATATAGTTAAGAATTGTTTTTGCCCTATTT
CCCTTAGTTCTTTCTCCTTCTCTCCAGTTTTATAGACCCTTGTGACTTTATTATGTCTTCACATTTTGATATCAT
TAATACGTGTTTCCTAATACATCTGCGATAACCAAGTAATGTATATGTTTTATTATACATCAACCTTTAATATAT
TATTTTTTCAAGTTCTGACTTTGTGTTTCGGAATCACTTGCATTAGGGTAGACTGTCTACGTCACACCTCATTGA
TGTACGCATTTTCCTGAATCCTACGTGAAGTTGTGAAGACGGAATGCTTCATGCACCCGACTTTTCCTTTGACTT
TTACTTTTTAATTTACAAAATTTATGGCAGTTTTTTCTTGATTTTCTCTCAGCCATTTTCAAGTGCTAATATAAG
```

AGCCAAACTATCAATTTGGATCACATATAATTAAGAAGCATAGATCACCACGATTTTGACATTGGAATTTGTTAA
ACACTTCAGATTCTAAGTGTTTCATTTCTCTCAATCATACTTCCACAAACCTTTTTGGTTTTCTAAACCTAGAGT
CATG

b. Identification of cis-acting elements including 5'UTR within the promoter sequences of *NtZIP5A* (from the contig AWOK01321153.1 position 22508…20712 orientation plus/minus – without ATG), **and *NtZIP5B*** (from the contig AWOJ01230056.1 position 1…1726 – without ATG).

For comparison made by Clustal Omega https://www.ebi.ac.uk/Tools/msa/clustalo/ the sequences of 1797 bp (for *NtZIP5A*) and 1726 bp (for *NtZIP5B*) upstream the START codon were used.
The program PlantCARE: http://bioinformatics.psb.ugent.be/webtools/plantcare/html/ , was used to identify *cis*-acting elements within identified promoter region. IDE2 I ZDRE sequences were not present in the PlantCARE database, therefore identification has been made based on the literature data.,

ABRE (ACGTG) -– cis-acting element involved in the abscisic responsiveness from *A.thaliana*
ABRE4 (CACGTA) -– ABA (abscisic acid) response from *Zea mays*
Box S (AGCCACC) – elicitation, wounding and pathogen responsiveness from *A.thaliana*
Box 4 (ATTAAT) **-** part of a conserved DNA module involved in light responsiveness from *Petroselinum crispum*
CAAT-box (CAAAT) - common cis-acting element in promoter and enhancer regions in *Pisum sativum*
G-box (TACGTG, CACGTC) – cis-acting regulatory element involved in light responsiveness, involved in regulating gene expression in response to a variety of environmental stresses and ABA from *A.thaliana*, *Brassica rapa*, *Capsicum annuum*, *Craterostigma plantagineum*, *Daucus carota*, *Helianthus annuus*, *Hordeum vulgare*, *Nicotiana tabacum*
GT1-motif (GGTTAA) – light responsive element from *A.thaliana*
IDE2 (CAAGTAA, CAAGTTC, CAAGTAC) – Iron Deficiency-responsive Element 2, IDEF2-binding core site (Ogo et.al., 2008)
MYB-like sequence (TAACCA) – possible MYB binding region from *A.thaiana*
P-box - (CCTTTTG) - gibberellin-responsive element from *Oryza sativa*
STRE (AGGGG) **-** activation by heat shock, osmotic stress, low pH, nutrient starvation from *A.thaliana*
TATA-box (ATATAA, ATTATA) - core promoter element around -30 of transcription start site.
TCT-motif (TCTTAC) **-** part of a light responsive element from *A.thaliana*
W box (TTGACC) – elicitation, wounding and pathogen responsiveness. Binds WRKY type transcription factors from *A. thaliana*
ZDRE (ATGTCGACAT) - Zinc Deficiency Response Element (Assunção et al., 2010), localized at – 603 bp (for *NtZIP5A*prom) and -580 bp (for *NtZIP5B*prom).

```
-1797 GAATATCATGGAGGAGGTTATGGAGTTAATCATAACTCCATAA---TTATTAGAGTATTG          promZIP5A_AWOK01321153.1
-1726 ---------------GGTCATATTTCAGCCCAAACGGCTAGATTTTAAATATGGCCGTT          promZIP5B_cloned
                     *** **    * *  *  ***  *   *   * * **  *   *

-1740 GGAGTTGTGGAGATAATTGAGAAGAGTAGACATCCACTATAATTTAATTTTCTTATAG-C          promZIP5A_AWOK01321153.1
-1681 GGAGAATTTTAATTTTTTTAAAAAAATAGCCGTTGGGCCCGCCAGGCCCGTCTGACGGTC          promZIP5B_cloned
      ****   *  *  *  ** * ** * *** * *                 *** *  * *

-1681 AATAGCTTAATTTTCGTGTGCGCATTATATAACTTTAATTATATTATTCTTGACTTTATT          promZIP5A_AWOK01321153.1
-1621 CCGGGCTAAACGGTCCCGGGCTCGTGGGCTAATA------CTACAAGACCGGCCCGTCAC          promZIP5B_cloned
          *** **   **  * ** * *    ***         **  *  *  * *  *
```

```
                          CAAT-box G-box ABRE
-1621 TAGTGTTATTTGTACATCATACCCCTTTACAATTTATAATTTAAGAAAATGATCTTTTTA   promZIP5A_AWOK01321153.1
-1567 G-GGCTTCTCAGGACCACCAGGACAGGCCCACCA----------------GGCCCGTTAG   promZIP5B_cloned
        *  ** *  * **  *     *     **                   *  *  **

                          CAAT-box G-box ABRE
-1561 GATCTCTTATGTGTAAAAGACAAATCTGTTACGTGTAAAAGTAAATCTCTCAAGTGTAAA   promZIP5A_AWOK01321153.1
-1524 GACCGCGTG----------CCCGGGCCGGTC----------------------------T   ZIP5B_AWOJ01230056.1
      ** * * *            *    * * *

                                                         CAAT-box W-box
-1501 AATTGAGGTAGGGTCATAAAACTAAAAACAAGATTGTAAAGAGCCACAAAATCAATTGAC   promZIP5A_AWOK01321153.1
-1505 GGTTCAGGCCCGGC------CCACCAAGCAGCCTTAATTACACCTATAAGAAAATTTGCG   promZIP5B_cloned
        ** ***   **        *   **  **   **    *  *  *  *  ** *  *  ***

                             CAAT-box G-box
-1441 CCCTTTATTTAGGGACGGCTAAACAAATTTAGTGGTCTAAACCCAAAATTTAATAAGAAT   promZIP5A_AWOK01321153.1
-1448 GGATT--TCACGGAAAATATATCGCTATAAACACGTCTAACTAATATGGATAAAAAGAAA   promZIP5B_cloned
         ** *  ** *  **      **   *   ******     *    *** *****

       CAAT-box                                          G-box ABRE4
-1381 CCAAATTTTATACACCTATATAAAGTTATCTTTTCTTTTTTTTCCGTACTTATTTATAGT   promZIP5A_AWOK01321153.1
-1390 CTAAAATAAG----ACGAACACCATTTATAACATATAAATTGTGTCAACAAGCACGTGGA   promZIP5B_cloned
      * *** *        * *     * ****    * *   ** *    **       * *

              TCT-motif          CAAT-box
-1321 ATTGTATTCTTACAAGATATAAGACTTTTAACTTTATTTAATAATATTATTATTATATTA   promZIP5A_AWOK01321153.1
-1334 ACGAAACTAAA-------GTAAGGGATCAAATGTATGTCACCAACACTCCAACGATATAT   promZIP5B_cloned
      *    * *           ****   *  **   *   * *  ** * *   *  ****
              Box 4
-1261 GCAAGGATTAATTCA---------AAAATGTTAGCCATATATTTGCATTACATTACCTTG   promZIP5A_AWOK01321153.1
-1281 AAAAAAAATACATCATCACGGCTAACTCTGTTTTCCATTAATTTGAACTTCATTCGCTTG   promZIP5B_cloned
        **  * **  ***         *   ****  ****  ***** * * ****  ****

-1210 AATCTTGTCGTAAAAGTTTTGATTTACTAA----TATGAAGGTTTAAGTAGTTTAATTCA   promZIP5A_AWOK01321153.1
-1221 AGCTTTAAATA-TGATCATTCATTAAATGTGGTATCCCTTGCTGAAAGTCCTAACCTTTA   promZIP5B_cloned
      *   **        *   ** *** * *      *     * *  ****  *    ** *

-1154 AAGTTCTAAAAAAATTCTA---ATGTTAAAAGGTAGACATTCTTTGTTTCTTTCAT----   promZIP5A_AWOK01321153.1
-1162 AACCTGAAAGAAATTAAGAAAAAATATTAAGAACTCTGTCTTTTTAGTATATCTTATCGGT   promZIP5B_cloned
      **  *  ** *** *   *   ** **** *  *     ** ** ** * * * **

-1101 TTTTACATAAAAT-----TTGTATTTG----TTTTTCTATACTTATGCATTTCACCATTA   promZIP5A_AWOK01321153.1
-1102 TTATCGATGAACTGATAATCGAAGAGGACAAAATCAAAATCGAAATCGATTACCCGATAA   promZIP5B_cloned
      ** *  ** ** *     * * *   *      *    **    **  *** * * ** *

                                                 CAAT-box
-1050 ATACTTTTTGGGCCTCAAAATTTTGGGGAGTAAAG---------CAAACTTTACTAGTCT   promZIP5A_AWOK01321153.1
-1042 GAAAAATTTCGAAATCGAAATCGAAATCGATAAACCGATAACAAATAATCGATTCGATTT   promZIP5B_cloned
        *   *** *   ** ****         ****            **         * *

              Box S
 -999 CACCCTTGAGCCACCCATGACTTTATTTACACTGG---ATTACGTTATCGTGATGACCTG   promZIP5A_AWOK01321153.1
 -985 ATCGATAAACCGATTCATGACTTTATTTACATTGGATTATTACGTTAAACTGATGACCTG   promZIP5B_cloned
        *  *  * * *  **************** ***   *********   **********

                   STRE
 -942 CTATCTCAATAAAGGGGTGCTAAGATTCAAAACCTTCTCGTTGTGTACTTTTATTCGACA   promZIP5A_AWOK01321153.1
 -922 CTATCTCAATA-GAGGGTGAGAGGATTCAAAACCTTCTCGTTATGTACTTTTAATTGATA   promZIP5B_cloned
      ***********   *****  * ******************* ********** * ** *

                           STRE GT1-motif
 -882 AATTAAAGAAGAATGAATAGAGGGGGCTAAGATTCAAAACCTTCTCGTTATGTACTTTTA   promZIP5A_AWOK01321153.1
 -863 AATTAAGGAAGAATGAATAGAGGGGGTTAAGATTCAAAACCTTCTCGTTATGTACTTTTA   promZIP5B_cloned
      ****** ******************* *********************************

           CAAT-box                        ABRE4
 -822 ATTGACAAATTAAAGGAGAATGACAAGAACGTTTTAGCACGTATAGAATTAGTTATTATA   promZIP5A_AWOK01321153.1
 -803 ATTGACAAATTAAAGGAGAATGACAAGAACGTTTTAGCACGTATAGAATTAGTTAATAAA   promZIP5B_cloned
      ******************************************************* ** *

                          CAAT-box            Box 4
 -762 ATTATAAGGAATTAAACACACAAATGTAATCAGGAAATAATTAATAGTAAAGAATAAATT   promZIP5A_AWOK01321153.1
 -743 ATTATAAGGAATTAAACAAACAAATTTAATCAGGAAATAATTAATAGTAAAGAATAAATT   promZIP5B_cloned
      ****************** ****** **********************************

-702 TGTTTAGAATCCAGCAAAAAA----AAAAAAATTATAAGCCAGCTAAGTAAGAGCAATTT   promZIP5A_AWOK01321153.1
 -683 TGTTTAGAATCCACAAAAATTTTAAAAAAATAAAATAAGCCAGCTAAGTAAGAGCAATTT   promZIP5B_cloned
      *************  ****      ***** *  **************************

                                                     ZDRE
 -646 ACGAGGAGGAAGTATATCCCGTTAATCTATTCAGCTAGAATATATGTCGACATTTTTTCG   promZIP5A_AWOK01321153.1
 -623 ACGAGGAGGAAGTATATCCCGTTAATCTATTCAGCTAGAATATATGTCGACATTTTTTCG   promZIP5B_cloned
      ************************************************************

 -586 ACACATAATGTAGTTAAGAATTGTTTTTGCTCTATTTCTCTCCGTTCTTTCTCCTTCTGT   promZIP5A_AWOK01321153.1
 -563 ACACATAATATAGTTAAGAATTGTTTTTGCCCTATTTCCCTTAGTTCTTTCTCCTTCTCT   promZIP5B_cloned
      ********* ********************  *******  **   *************** *

                                                          G-box ABRE
 -526 CCAGTTTTATAGACCCTTGTGACTTTATTATGTCTTCACTTTTTGATATCATTAATACGT   promZIP5A_AWOK01321153.1
 -503 CCAGTTTTATAGACCCTTGTGACTTTATTATGTCTTCACATTTTGATATCATTAATACGT   promZIP5B_cloned
      *************************************** ********************
```

```
                     MYB-like sequence  IDE2           TATA-box
-466 GTTTCCTAATACATCTGCGATAACCAAGTAATGTATATGTTCTATTATACATTGATCGAC     promZIP5A_AWOK01321153.1
-443 GTTTCCTAATACATCTGCGATAACCAAGTAATGTATATGTTTTATTATACA----TCAAC     promZIP5B_cloned
     ***************************************** *********    ** **

                         IDE2
-406 CTTTCATATATTATTTTTTCAGGTTTTGACTTTGTGTTGCGAAATCACTTGTATTAGGGT     promZIP5A_AWOK01321153.1
-387 CTTTAATATATTATTTTTTCAAGTTCTGACTTTGTGTTTCGGAATCACTTGCATTAGGGT     promZIP5B_cloned
     **** **************** *** ************ ** ********* ********

                                                  G-box ABRE
-346 AGACGGTCTACATTACATTCCTTTGAAGTGTGCCTTTTCCTGAATCCGACGTGCAAACGA     promZIP5A_AWOK01321153.1
-327 AGACTGTCTACGTCACACCTCATTGATGTACGCATTTTCCTGAATCCTACGTGAAGTTGT     promZIP5B_cloned
     **** ****** * ***   * **** **  ** ************* ***** *   *

                                   P-box
-286 GGTTACGGGATGCTTCATGCATTTGATAATCCTTTTGACTTTTACTTTTTAATTTCACAA     promZIP5A_AWOK01321153.1
-267 GAAGACGGAATGCTTCATGCACCCGACTTTTCCTTTGACTTTTACTTTTTAATTTACAAA     promZIP5B_cloned
     *   **** ************   **   * * **********************   **

-226 AAGTTGTGACAGTTTTTTCTTGGTTATCTCTCTGCCATTTTCTTTATTAACACTTCATGT     promZIP5A_AWOK01321153.1
-207 -ATTTATGGCAGTTTTTTCTTGATTTTCTCTC--------------------AGCCATTT     promZIP5B_cloned
      * ** ** ************* ** ******                       *** *

       IDE2     TATA-box                            TATA-box
-166 TCAAGTACTAATATAAGAGCTAAACTATCAATTAGGATCACAGATAATTAAGAAGCCTAT     promZIP5A_AWOK01321153.1
-168 TCAAGTGCTAATATAAGAGCCAAACTATCAATTTGGATCACATATAATTAAGAAGCATAG     promZIP5B_cloned
     ****** ************* ************ ******** ************* **

-106 CTCACCACGATTGTGACATTGGAATTTGTA--ACATTTCAGATTCTAAGTGATTCTTTTC     promZIP5A_AWOK01321153.1
-108 ATCACCACGATTTTGACATTGGAATTTGTTAAACACTTCAGATTCTAAGTGTTTCATTTC     promZIP5B_cloned
      *********** ****************   *** *************** *** ****

 -48 TCTCAATCGTACTTCCAAAACCCTTTTTCATTTTCTATACCTAGAGTCATG              promZIP5A_AWOK01321153.1
 -48 TCTCAATCATACTTCCACAAACCTTTTTGGTTTTCTAAACCTAGAGTCATG              promZIP5B_cloned
     ******** ******** ** *******  ******* *************
```

c. Comparison of the promoter sequences including 5’UTR of *NtZIP5A* from three tobacco cultivars: K326 Nitab4.5 (NCAA01000170.1 position 898333…896770, orientation plus/minus), **Basma Xanthi** (AWOK01321153.1 position 22508…20712, orientation plus/minus) and **K326** (AWOJ01501266.1 position 7785…6182, orientation plus/minus).

For comparison the following fragments were used: 1564 bp (cultivar **K326 Nitab4.5**), 1797 bp (cultivar **Basma Xanthi**) and 1604 bp (cultivar **K326**) upstream of the START codon. Comparison was made with the Clustal Omega https://www.ebi.ac.uk/Tools/msa/clustalo/
In blue - differences between sequences;
* depicts identical nucleotides at a given position.

```
PromZIP5A_NCAA01000170.1      ------------------------------------------------------------      0
PromZIP5A_AWOK01321153.1      GAATATCATGGAGGAGGTTATGGAGTTAATCATAACTCCATAATTATTAGAGTATTGGGA      60
PromZIP5A_AWOJ01501266.1      ------------------------------------------------------------      0

PromZIP5A_NCAA01000170.1      ------------------------------------------------------------      0
PromZIP5A_AWOK01321153.1      GTTGTGGAGATAATTGAGAAGAGTAGACATCCACTATAATTTAATTTTCTTATAGCAATA      120
PromZIP5A_AWOJ01501266.1      ------------------------------------------------------------      0

PromZIP5A_NCAA01000170.1      ------------------------------------------------------------      0
PromZIP5A_AWOK01321153.1      GCTTAATTTTCGTGTGCGCATTATATAACTTTAATTATATTATTCTTGACTTTATTTAGT      180
PromZIP5A_AWOJ01501266.1      ------------------------------------------------------------      0

PromZIP5A_NCAA01000170.1      ----------------------------------------------------TTTAGATC      8
PromZIP5A_AWOK01321153.1      GTTATTTGTACATCATACCCCTTTACAATTTATAATTTAAGAAAATGATCTTTTTAGATC      240
PromZIP5A_AWOJ01501266.1      -------------CATACCCCTTTACAATTTATAATTTAAGAAAATGATCTTTTTAGATC      47
                                                                                  ********

PromZIP5A_NCAA01000170.1      TCTTATGTGTAAAAGACAAATCTGTTACGTGTAAAAGTAAATCTCTCAAGTGTAAAAATT      68
PromZIP5A_AWOK01321153.1      TCTTATGTGTAAAAGACAAATCTGTTACGTGTAAAAGTAAATCTCTCAAGTGTAAAAATT      300
PromZIP5A_AWOJ01501266.1      TCTTATGTGTAAAAGACAAATCTGTTACGTGTAAAAGTAAATCTCTCAAGTGTAAAAATT      107
                              ************************************************************

PromZIP5A_NCAA01000170.1      GAGGTAGGGTCATAAAACTAAAAACAAGATTGTAAAGAGCCACAAAATCAATTGACCCCT      128
PromZIP5A_AWOK01321153.1      GAGGTAGGGTCATAAAACTAAAAACAAGATTGTAAAGAGCCACAAAATCAATTGACCCCT      360
PromZIP5A_AWOJ01501266.1      GAGGTAGGGTCATAAAACTAAAAACAAGATTGTAAAGAGCCACAAAATCAATTGACCCCT      167
                              ************************************************************

PromZIP5A_NCAA01000170.1      TTATTTAGGGACGGCTAAACAAATTTAGTGGTCTAAACCCAAAATTTAATAAGAATCCAA      188
PromZIP5A_AWOK01321153.1      TTATTTAGGGACGGCTAAACAAATTTAGTGGTCTAAACCCAAAATTTAATAAGAATCCAA      420
PromZIP5A_AWOJ01501266.1      TTATTTAGGGACGGCTAAACAAATTTAGTGGTCTAAACCCAAAATTTAATAAGAATCCAA      227
                              ************************************************************

PromZIP5A_NCAA01000170.1      ATTTTATACACCTATATAAAGTTATCTTTTCTTTTTTTTCCGTACTTATTTATAGTATTG      248
PromZIP5A_AWOK01321153.1      ATTTTATACACCTATATAAAGTTATCTTTTCTTTTTTTTCCGTACTTATTTATAGTATTG      480
PromZIP5A_AWOJ01501266.1      ATTTTATACACCTATATAAAGTTATCTTTTCTTTTTTTTCCGTACTTATTTATAGTATTG      287
                              ************************************************************

PromZIP5A_NCAA01000170.1      TATTCTTACAAGATATAAGACTTTTAACTTTATTTAATAATATTATTATTATATTAGCAA      308
PromZIP5A_AWOK01321153.1      TATTCTTACAAGATATAAGACTTTTAACTTTATTTAATAATATTATTATTATATTAGCAA      540
PromZIP5A_AWOJ01501266.1      TATTCTTACAAGATATAAGACTTTTAACTTTATTTAATAATATTATTATTATATTAGCAA      347
                              ************************************************************

PromZIP5A_NCAA01000170.1      GGATTAATTCAAAAATGTTAGCCATATATTTGCATTACATTACCTTGAATCTTGTCGTAA      368
PromZIP5A_AWOK01321153.1      GGATTAATTCAAAAATGTTAGCCATATATTTGCATTACATTACCTTGAATCTTGTCGTAA      600
PromZIP5A_AWOJ01501266.1      GGATTAATTCAAAAATGTTAGCCATATATTTGCATTACATTACCTTGAATCTTGTCGTAA      407
                              ************************************************************

PromZIP5A_NCAA01000170.1      AAGTTTTGATTTACTAATATGAAGGTTTAAGTAGTTTAATTCAAAGTTCTAAAAAAATTC      428
PromZIP5A_AWOK01321153.1      AAGTTTTGATTTACTAATATGAAGGTTTAAGTAGTTTAATTCAAAGTTCTAAAAAAATTC      660
PromZIP5A_AWOJ01501266.1      AAGTTTTGATTTACTAATATGAAGGTTTAAGTAGTTTAATTCAAAGTTCTAAAAAAATTC      467
                              ************************************************************

PromZIP5A_NCAA01000170.1      TAATGTTAAAAGGTAGACATTCTTTGTTTCTTTCATTTTTACATAAAATTTGTATTTGTT      488
PromZIP5A_AWOK01321153.1      TAATGTTAAAAGGTAGACATTCTTTGTTTCTTTCATTTTTACATAAAATTTGTATTTGTT      720
PromZIP5A_AWOJ01501266.1      TAATGTTAAAAGGTAGACATTCTTTGTTTCTTTCATTTTTACATAAAATTTGTATTTGTT      527
                              ************************************************************

PromZIP5A_NCAA01000170.1      TTTCTATACTTATGCATTTCACCATTAATACTTTTTGGGCCTCAAAATTTTGGGGAGTAA      548
PromZIP5A_AWOK01321153.1      TTTCTATACTTATGCATTTCACCATTAATACTTTTTGGGCCTCAAAATTTTGGGGAGTAA      780
PromZIP5A_AWOJ01501266.1      TTTCTATACTTATGCATTTCACCATTAATACTTTTTGGGCCTCAAAATTTTGGGGAGTAA      587
                              ************************************************************

PromZIP5A_NCAA01000170.1      AGCAAACTTTACTAGTCTCACCCTTGAGCCACCCATGACTTTATTTACACTGGATTACGT      608
PromZIP5A_AWOK01321153.1      AGCAAACTTTACTAGTCTCACCCTTGAGCCACCCATGACTTTATTTACACTGGATTACGT      840
```

```
PromZIP5A_AWOJ01501266.1      AGCAAACTTTACTAGTCTCACCCTTGAGCCACCCATGACTTTATTTACACTGGATTACGT      647
                              ************************************************************

PromZIP5A_NCAA01000170.1      TATCGTGATGACCTGCTATCTCAATAAAGGGGTGCTAAGATTCAAAACCTTCTCGTTGTG      668
PromZIP5A_AWOK01321153.1      TATCGTGATGACCTGCTATCTCAATAAAGGGGTGCTAAGATTCAAAACCTTCTCGTTGTG      900
PromZIP5A_AWOJ01501266.1      TATCGTGATGACCTGCTATCTCAATAAAGGGGTGCTAAGATTCAAAACCTTCTCGTTGTG      707
                              ************************************************************

PromZIP5A_NCAA01000170.1      TACTTTTATTCGACAAATTAAAGAAGAATGAATAGAGGGGGCTAAGATTCAAAACCTTCT      728
PromZIP5A_AWOK01321153.1      TACTTTTATTCGACAAATTAAAGAAGAATGAATAGAGGGGGCTAAGATTCAAAACCTTCT      960
PromZIP5A_AWOJ01501266.1      TACTTTTATTCGACAAATTAAAGAAGAATGAATAGAGGGGGCTAAGATTCAAAACCTTCT      767
                              ************************************************************

PromZIP5A_NCAA01000170.1      CGTTATGTACTTTTAATTGACAAATTAAAGGAGAATGACAAGAACGTTTTAGCACGTATA      788
PromZIP5A_AWOK01321153.1      CGTTATGTACTTTTAATTGACAAATTAAAGGAGAATGACAAGAACGTTTTAGCACGTATA      1020
PromZIP5A_AWOJ01501266.1      CGTTATGTACTTTTAATTGACAAATTAAAGGAGAATGACAAGAACGTTTTAGCACGTATA      827
                              ************************************************************

PromZIP5A_NCAA01000170.1      GAATTAGTTATTATAATTATAAGGAATTAAACACACAAATGTAATCAGGAAATAATTAAT      848
PromZIP5A_AWOK01321153.1      GAATTAGTTATTATAATTATAAGGAATTAAACACACAAATGTAATCAGGAAATAATTAAT      1080
PromZIP5A_AWOJ01501266.1      GAATTAGTTATTATAATTATAAGGAATTAAACACACAAATGTAATCAGGAAATAATTAAT      887
                              ************************************************************

PromZIP5A_NCAA01000170.1      AGTAAAGAATAAATTTGTTTAGAATCCAGCAAAAAA-AAAAAATTATAAGCCAGCTAAGT      907
PromZIP5A_AWOK01321153.1      AGTAAAGAATAAATTTGTTTAGAATCCAGCAAAAAAAAAAAAATTATAAGCCAGCTAAGT      1140
PromZIP5A_AWOJ01501266.1      AGTAAAGAATAAATTTGTTTAGAATCCAGCAAAAAAAAAAAAATTATAAGCCAGCTAAGT      947
                              ************************************ ***********************

PromZIP5A_NCAA01000170.1      AAGAGCAATTTACGAGGAGGAAGTATATCCCGTTAATCTATTCAGCTAGAATATATGTCG      967
PromZIP5A_AWOK01321153.1      AAGAGCAATTTACGAGGAGGAAGTATATCCCGTTAATCTATTCAGCTAGAATATATGTCG      1200
PromZIP5A_AWOJ01501266.1      AAGAGCAATTTACGAGGAGGAAGTATATCCCGTTAATCTATTCAGCTAGAATATATGTCG      1007
                              ************************************************************

PromZIP5A_NCAA01000170.1      ACATTTTTTCGACACATAATGTAGTTAAGAATTGTTTTTGCTCTATTTCTCTCCGTTCTT      1027
PromZIP5A_AWOK01321153.1      ACATTTTTTCGACACATAATGTAGTTAAGAATTGTTTTTGCTCTATTTCTCTCCGTTCTT      1260
PromZIP5A_AWOJ01501266.1      ACATTTTTTCGACACATAATGTAGTTAAGAATTGTTTTTGCTCTATTTCTCTCCGTTCTT      1067
                              ************************************************************

PromZIP5A_NCAA01000170.1      TCTCCTTCTGTCCAGTTTTATAGACCCTTGTGACTTTATTATGTCTTCACTTTTTGATAT      1087
PromZIP5A_AWOK01321153.1      TCTCCTTCTGTCCAGTTTTATAGACCCTTGTGACTTTATTATGTCTTCACTTTTTGATAT      1320
PromZIP5A_AWOJ01501266.1      TCTCCTTCTGTCCAGTTTTATAGACCCTTGTGACTTTATTATGTCTTCACTTTTTGATAT      1127
                              ************************************************************

PromZIP5A_NCAA01000170.1      CATTAATACGTGTTTCCTAATACATCTGCGATAACCAAGTAATGTATATGTTCTATTATA      1147
PromZIP5A_AWOK01321153.1      CATTAATACGTGTTTCCTAATACATCTGCGATAACCAAGTAATGTATATGTTCTATTATA      1380
PromZIP5A_AWOJ01501266.1      CATTAATACGTGTTTCCTAATACATCTGCGATAACCAAGTAATGTATATGTTCTATTATA      1187
                              ************************************************************

PromZIP5A_NCAA01000170.1      CATTGATCGACCTTTCATATATTATTTTTTCAGGTTTTGACTTTGTGTTGCGAAATCACT      1207
PromZIP5A_AWOK01321153.1      CATTGATCGACCTTTCATATATTATTTTTTCAGGTTTTGACTTTGTGTTGCGAAATCACT      1440
PromZIP5A_AWOJ01501266.1      CATTGATCGACCTTTCATATATTATTTTTTCAGGTTTTGACTTTGTGTTGCGAAATCACT      1247
                              ************************************************************

PromZIP5A_NCAA01000170.1      TGTATTAGGGTAGACGGTCTACATTACATTCCTTTGAAGTGTGCCTTTTCCTGAATCCGA      1267
PromZIP5A_AWOK01321153.1      TGTATTAGGGTAGACGGTCTACATTACATTCCTTTGAAGTGTGCCTTTTCCTGAATCCGA      1500
PromZIP5A_AWOJ01501266.1      TGTATTAGGGTAGACGGTCTACATTACATTCCTTTGAAGTGTGCCTTTTCCTGAATCCGA      1307
                              ************************************************************

PromZIP5A_NCAA01000170.1      CGTGCAAACGAGGTTACGGGATGCTTCATGCATTTGATAATCCTTTTGACTTTTACTTTT      1327
PromZIP5A_AWOK01321153.1      CGTGCAAACGAGGTTACGGGATGCTTCATGCATTTGATAATCCTTTTGACTTTTACTTTT      1560
PromZIP5A_AWOJ01501266.1      CGTGCAAACGAGGTTACGGGATGCTTCATGCATTTGATAATCCTTTTGACTTTTACTTTT      1367
                              ************************************************************

PromZIP5A_NCAA01000170.1      TAATTTCACAAAAGTTGTGACAGTTTTTTCTTGGTTATCTCTCTGCCATTTTCTTTATTA      1387
PromZIP5A_AWOK01321153.1      TAATTTCACAAAAGTTGTGACAGTTTTTTCTTGGTTATCTCTCTGCCATTTTCTTTATTA      1620
PromZIP5A_AWOJ01501266.1      TAATTTCACAAAAGTTGTGACAGTTTTTTCTTGGTTATCTCTCTGCCATTTTCTTTATTA      1427
                              ************************************************************

PromZIP5A_NCAA01000170.1      ACACTTCATGTTCAAGTACTAATATAAGAGCTAAACTATCAATTAGGATCACAGATAATT      1447
PromZIP5A_AWOK01321153.1      ACACTTCATGTTCAAGTACTAATATAAGAGCTAAACTATCAATTAGGATCACAGATAATT      1680
PromZIP5A_AWOJ01501266.1      ACACTTCATGTTCAAGTACTAATATAAGAGCTAAACTATCAATTAGGATCACAGATAATT      1487
                              ************************************************************

PromZIP5A_NCAA01000170.1      AAGAAGCCTATCTCACCACGATTGTGACATTGGAATTTGTAACATTTCAGATTCTAAGTG      1507
PromZIP5A_AWOK01321153.1      AAGAAGCCTATCTCACCACGATTGTGACATTGGAATTTGTAACATTTCAGATTCTAAGTG      1740
PromZIP5A_AWOJ01501266.1      AAGAAGCCTATCTCACCACGATTGTGACATTGGAATTTGTAACATTTCAGATTCTAAGTG      1547
                              ************************************************************

PromZIP5A_NCAA01000170.1      ATTCTTTTCTCTCAATCGTACTTCCAAAACCCTTTTTCATTTTCTATACCTAGAGTC         1564
PromZIP5A_AWOK01321153.1      ATTCTTTTCTCTCAATCGTACTTCCAAAACCCTTTTTCATTTTCTATACCTAGAGTC         1797
PromZIP5A_AWOJ01501266.1      ATTCTTTTCTCTCAATCGTACTTCCAAAACCCTTTTTCATTTTCTATACCTAGAGTC         1604
                              *********************************************************
```

d. Comparison of the promoter sequences including 5'UTR of *NtZIP5B* from four tobacco cultivars: K326 Nitab4.5 (NCAA01006169.1 position 29448…27794, orientation plus/minus), **Basma Xanthi** (AWOK01242628.1 position 1…1607) and **K326** (AWOJ01230056.1 position 1…1726).

For comparison the following fragments were used: 1655 bp (cultivar **K326 Nitab4.5**), 1607 bp (cultivar **Basma Xanthi**) and 1726 bp (cultivar **K326**) upstream from the START codon. Comparison was made with the Clustal Omega https://www.ebi.ac.uk/Tools/msa/clustalo/
* depicts identical nucleotides at a given position.

```
PromZIP5B_NCAA01006169.1      ------------------------------------------------------------      0
PromZIP5B_AWOK01242628.1      ------------------------------------------------------------      0
PromZIP5B_AWOJ01230056.1      GGTCATATTTTCAGCCCAAACGGCTAGATTTTAAATATGGCCGTTGGAGAATTTTAATTT      60


PromZIP5B_NCAA01006169.1      -----------TAGCCGTTGGGCCCGCCAGGCCCGTCTGACGGTCCCGGGCTAAACGGTC      49
PromZIP5B_AWOK01242628.1      -----------------------------------------------------------C      1
PromZIP5B_AWOJ01230056.1      TTTAAAAAAAATAGCCGTTGGGCCCGCCAGGCCCGTCTGACGGTCCCGGGCTAAACGGTC      120
                                                                                         *

PromZIP5B_NCAA01006169.1      CCGGGCTCGTGGGCTAATACTACAAGACCGGCCCGTCACGGGCTTCTCAGGACCACCAGG      109
PromZIP5B_AWOK01242628.1      CCGGGCTCGTGGGCTAATACTACAAGACCGGCCCGTCACGGGCTTCTCAGGACCACCAGG      61
PromZIP5B_AWOJ01230056.1      CCGGGCTCGTGGGCTAATACTACAAGACCGGCCCGTCACGGGCTTCTCAGGACCACCAGG      180
                              ************************************************************

PromZIP5B_NCAA01006169.1      ACAGGCCCACCAGGCCCGTTAGGACCGCGTGCCCGGGCCGGTCTGGTTCAGGCCCGGCCC      169
PromZIP5B_AWOK01242628.1      ACAGGCCCACCAGGCCCGTTAGGACCGCGTGCCCGGGCCGGTCTGGTTCAGGCCCGGCCC      121
PromZIP5B_AWOJ01230056.1      ACAGGCCCACCAGGCCCGTTAGGACCGCGTGCCCGGGCCGGTCTGGTTCAGGCCCGGCCC      240
                              ************************************************************

PromZIP5B_NCAA01006169.1      ACCAAGCAGCCTTAATTACACCTATAAGAAAATTTGCGGGATTTCACGGAAAATATATCG      229
PromZIP5B_AWOK01242628.1      ACCAAGCAGCCTTAATTACACCTATAAGAAAATTTGCGGGATTTCACGGAAAATATATCG      181
PromZIP5B_AWOJ01230056.1      ACCAAGCAGCCTTAATTACACCTATAAGAAAATTTGCGGGATTTCACGGAAAATATATCG      300
                              ************************************************************

PromZIP5B_NCAA01006169.1      CTATAAACACGTCTAACTAATATGGATAAAAAGAAACTAAAATAAGACGAACACCATTTA      289
PromZIP5B_AWOK01242628.1      CTATAAACACGTCTAACTAATATGGATAAAAAGAAACTAAAATAAGACGAACACCATTTA      241
PromZIP5B_AWOJ01230056.1      CTATAAACACGTCTAACTAATATGGATAAAAAGAAACTAAAATAAGACGAACACCATTTA      360
                              ************************************************************

PromZIP5B_NCAA01006169.1      TAACATATAAATTGTGTCAACAAGCACGTGGAACGAAACTAAAGTAAGGGATCAAATGTA      349
PromZIP5B_AWOK01242628.1      TAACATATAAATTGTGTCAACAAGCACGTGGAACGAAACTAAAGTAAGGGATCAAATGTA      301
PromZIP5B_AWOJ01230056.1      TAACATATAAATTGTGTCAACAAGCACGTGGAACGAAACTAAAGTAAGGGATCAAATGTA      420
                              ************************************************************

PromZIP5B_NCAA01006169.1      TGTCACCAACACTCCAACGATATATAAAAAAAATACATCATCACGGCTAACTCTGTTTTC      409
PromZIP5B_AWOK01242628.1      TGTCACCAACACTCCAACGATATATAAAAAAAATACATCATCACGGCTAACTCTGTTTTC      361
PromZIP5B_AWOJ01230056.1      TGTCACCAACACTCCAACGATATATAAAAAAAATACATCATCACGGCTAACTCTGTTTTC      480
                              ************************************************************

PromZIP5B_NCAA01006169.1      CATTAATTTGAACTTCATTCGCTTGAGCTTTAAATATGATCATTCATTAAATGTGGTATC      469
PromZIP5B_AWOK01242628.1      CATTAATTTGAACTTCATTCGCTTGAGCTTTAAATATGATCATTCATTAAATGTGGTATC      421
PromZIP5B_AWOJ01230056.1      CATTAATTTGAACTTCATTCGCTTGAGCTTTAAATATGATCATTCATTAAATGTGGTATC      540
                              ************************************************************

PromZIP5B_NCAA01006169.1      CCTTGCTGAAAGTCCTAACCTTTAAACCTGAAAGAAATTAAGAAAAATATTAAGAACTCT      529
PromZIP5B_AWOK01242628.1      CCTTGCTGAAAGTCCTAACCTTTAAACCTGAAAGAAATTAAGAAAAATATTAAGAACTCT      481
PromZIP5B_AWOJ01230056.1      CCTTGCTGAAAGTCCTAACCTTTAAACCTGAAAGAAATTAAGAAAAATATTAAGAACTCT      600
                              ************************************************************

PromZIP5B_NCAA01006169.1      GTCTTTTTAGTATATCTTATCGGTTTATCGATGAACTGATAATCGAAGAGGACAAAATCA      589
PromZIP5B_AWOK01242628.1      GTCTTTTTAGTATATCTTATCGGTTTATCGATGAACTGATAATCGAAGAGGACAAAATCA      541
PromZIP5B_AWOJ01230056.1      GTCTTTTTAGTATATCTTATCGGTTTATCGATGAACTGATAATCGAAGAGGACAAAATCA      660
                              ************************************************************

PromZIP5B_NCAA01006169.1      AAATCGAAATCGATTACCCGATAAGAAAAATTTCGAAATCGAAATCGAAATCGATAAACC      649
PromZIP5B_AWOK01242628.1      AAATCGAAATCGATTACCCGATAAGAAAAATTTCGAAATCGAAATCGAAATCGATAAACC      601
PromZIP5B_AWOJ01230056.1      AAATCGAAATCGATTACCCGATAAGAAAAATTTCGAAATCGAAATCGAAATCGATAAACC      720
                              ************************************************************

PromZIP5B_NCAA01006169.1      GATAACAAATAATCGATTCGATTTATCGATAAACCGATTCATGACTTTATTTACATTGGA      709
PromZIP5B_AWOK01242628.1      GATAACAAATAATCGATTCGATTTATCGATAAACCGATTCATGACTTTATTTACATTGGA      661
PromZIP5B_AWOJ01230056.1      GATAACAAATAATCGATTCGATTTATCGATAAACCGATTCATGACTTTATTTACATTGGA      780
                              ************************************************************

PromZIP5B_NCAA01006169.1      TTATTACGTTAAACTGATGACCTGCTATCTCAATAGAGGGTGAGAGGATTCAAAACCTTC      769
PromZIP5B_AWOK01242628.1      TTATTACGTTAAACTGATGACCTGCTATCTCAATAGAGGGTGAGAGGATTCAAAACCTTC      721
PromZIP5B_AWOJ01230056.1      TTATTACGTTAAACTGATGACCTGCTATCTCAATAGAGGGTGAGAGGATTCAAAACCTTC      840
                              ************************************************************
```

```
PromZIP5B_NCAA01006169.1      TCGTTATGTACTTTTAATTGATAAATTAAGGAAGAATGAATAGAGGGGGTTAAGATTCAA        829
PromZIP5B_AWOK01242628.1      TCGTTATGTACTTTTAATTGATAAATTAAGGAAGAATGAATAGAGGGGGTTAAGATTCAA        781
PromZIP5B_AWOJ01230056.1      TCGTTATGTACTTTTAATTGATAAATTAAGGAAGAATGAATAGAGGGGGTTAAGATTCAA        900
                              ************************************************************

PromZIP5B_NCAA01006169.1      AACCTTCTCGTTATGTACTTTTAATTGACAAATTAAAGGAGAATGACAAGAACGTTTTAG        889
PromZIP5B_AWOK01242628.1      AACCTTCTCGTTATGTACTTTTAATTGACAAATTAAAGGAGAATGACAAGAACGTTTTAG        841
PromZIP5B_AWOJ01230056.1      AACCTTCTCGTTATGTACTTTTAATTGACAAATTAAAGGAGAATGACAAGAACGTTTTAG        960
                              ************************************************************

PromZIP5B_NCAA01006169.1      CACGTATAGAATTAGTTAATAAAATTATAAGGAATTAAACAAACAAATTTAATCAGGAAA        949
PromZIP5B_AWOK01242628.1      CACGTATAGAATTAGTTAATAAAATTATAAGGAATTAAACAAACAAATTTAATCAGGAAA        901
PromZIP5B_AWOJ01230056.1      CACGTATAGAATTAGTTAATAAAATTATAAGGAATTAAACAAACAAATTTAATCAGGAAA        1020
                              ************************************************************

PromZIP5B_NCAA01006169.1      TAATTAATAGTAAAGAATAAATTTGTTTAGAATCCACAAAAATTTTAAAAAAATAAAATA        1009
PromZIP5B_AWOK01242628.1      TAATTAATAGTAAAGAATAAATTTGTTTAGAATCCACAAAAATTTTAAAAAAATAAAATA        961
PromZIP5B_AWOJ01230056.1      TAATTAATAGTAAAGAATAAATTTGTTTAGAATCCACAAAAATTTTAAAAAAATAAAATA        1080
                              ************************************************************

PromZIP5B_NCAA01006169.1      AGCCAGCTAAGTAAGAGCAATTTACGAGGAGGAAGTATATCCCGTTAATCTATTCAGCTA        1069
PromZIP5B_AWOK01242628.1      AGCCAGCTAAGTAAGAGCAATTTACGAGGAGGAAGTATATCCCGTTAATCTATTCAGCTA        1021
PromZIP5B_AWOJ01230056.1      AGCCAGCTAAGTAAGAGCAATTTACGAGGAGGAAGTATATCCCGTTAATCTATTCAGCTA        1140
                              ************************************************************

PromZIP5B_NCAA01006169.1      GAATATATGTCGACATTTTTTCGACACATAATATAGTTAAGAATTGTTTTTGCCCTATTT        1129
PromZIP5B_AWOK01242628.1      GAATATATGTCGACATTTTTTCGACACATAATATAGTTAAGAATTGTTTTTGCCCTATTT        1081
PromZIP5B_AWOJ01230056.1      GAATATATGTCGACATTTTTTCGACACATAATATAGTTAAGAATTGTTTTTGCCCTATTT        1200
                              ************************************************************

PromZIP5B_NCAA01006169.1      CCCTTAGTTCTTTCTCCTTCTCTCCAGTTTTATAGACCCTTGTGACTTTATTATGTCTTC        1189
PromZIP5B_AWOK01242628.1      CCCTTAGTTCTTTCTCCTTCTCTCCAGTTTTATAGACCCTTGTGACTTTATTATGTCTTC        1141
PromZIP5B_AWOJ01230056.1      CCCTTAGTTCTTTCTCCTTCTCTCCAGTTTTATAGACCCTTGTGACTTTATTATGTCTTC        1260
                              ************************************************************

PromZIP5B_NCAA01006169.1      ACATTTTGATATCATTAATACGTGTTTCCTAATACATCTGCGATAACCAAGTAATGTATA        1249
PromZIP5B_AWOK01242628.1      ACATTTTGATATCATTAATACGTGTTTCCTAATACATCTGCGATAACCAAGTAATGTATA        1201
PromZIP5B_AWOJ01230056.1      ACATTTTGATATCATTAATACGTGTTTCCTAATACATCTGCGATAACCAAGTAATGTATA        1320
                              ************************************************************

PromZIP5B_NCAA01006169.1      TGTTTTATTATACATCAACCTTTAATATATTATTTTTTCAAGTTCTGACTTTGTGTTTCG        1309
PromZIP5B_AWOK01242628.1      TGTTTTATTATACATCAACCTTTAATATATTATTTTTTCAAGTTCTGACTTTGTGTTTCG        1261
PromZIP5B_AWOJ01230056.1      TGTTTTATTATACATCAACCTTTAATATATTATTTTTTCAAGTTCTGACTTTGTGTTTCG        1380
                              ************************************************************

PromZIP5B_NCAA01006169.1      GAATCACTTGCATTAGGGTAGACTGTCTACGTCACACCTCATTGATGTACGCATTTTCCT        1369
PromZIP5B_AWOK01242628.1      GAATCACTTGCATTAGGGTAGACTGTCTACGTCACACCTCATTGATGTACGCATTTTCCT        1321
PromZIP5B_AWOJ01230056.1      GAATCACTTGCATTAGGGTAGACTGTCTACGTCACACCTCATTGATGTACGCATTTTCCT        1440
                              ************************************************************

PromZIP5B_NCAA01006169.1      GAATCCTACGTGAAGTTGTGAAGACGGAATGCTTCATGCACCCGACTTTTCCTTTGACTT        1429
PromZIP5B_AWOK01242628.1      GAATCCTACGTGAAGTTGTGAAGACGGAATGCTTCATGCACCCGACTTTTCCTTTGACTT        1381
PromZIP5B_AWOJ01230056.1      GAATCCTACGTGAAGTTGTGAAGACGGAATGCTTCATGCACCCGACTTTTCCTTTGACTT        1500
                              ************************************************************

PromZIP5B_NCAA01006169.1      TTACTTTTTAATTTACAAAATTTATGGCAGTTTTTTCTTGATTTTCTCTCAGCCATTTTC        1489
PromZIP5B_AWOK01242628.1      TTACTTTTTAATTTACAAAATTTATGGCAGTTTTTTCTTGATTTTCTCTCAGCCATTTTC        1441
PromZIP5B_AWOJ01230056.1      TTACTTTTTAATTTACAAAATTTATGGCAGTTTTTTCTTGATTTTCTCTCAGCCATTTTC        1560
                              ************************************************************

PromZIP5B_NCAA01006169.1      AAGTGCTAATATAAGAGCCAAACTATCAATTTGGATCACATATAATTAAGAAGCATAGAT        1549
PromZIP5B_AWOK01242628.1      AAGTGCTAATATAAGAGCCAAACTATCAATTTGGATCACATATAATTAAGAAGCATAGAT        1501
PromZIP5B_AWOJ01230056.1      AAGTGCTAATATAAGAGCCAAACTATCAATTTGGATCACATATAATTAAGAAGCATAGAT        1620
                              ************************************************************

PromZIP5B_NCAA01006169.1      CACCACGATTTTGACATTGGAATTTGTTAAACACTTCAGATTCTAAGTGTTTCATTTCTC        1609
PromZIP5B_AWOK01242628.1      CACCACGATTTTGACATTGGAATTTGTTAAACACTTCAGATTCTAAGTGTTTCATTTCTC        1561
PromZIP5B_AWOJ01230056.1      CACCACGATTTTGACATTGGAATTTGTTAAACACTTCAGATTCTAAGTGTTTCATTTCTC        1680
                              ************************************************************

PromZIP5B_NCAA01006169.1      TCAATCATACTTCCACAAACCTTTTTGGTTTTCTAAACCTAGAGTC          1655
PromZIP5B_AWOK01242628.1      TCAATCATACTTCCACAAACCTTTTTGGTTTTCTAAACCTAGAGTC          1607
PromZIP5B_AWOJ01230056.1      TCAATCATACTTCCACAAACCTTTTTGGTTTTCTAAACCTAGAGTC          1726
                              **********************************************
```
