# Supplementary material for: Zn/Cd status-dependent accumulation of Zn and Cd in root parts in tobacco is accompanied by specific expression of ZIP genes
Source: BMC Plant Biol. 2020 Jan 22;20:37. doi: 10.1186/s12870-020-2255-3 (PMC6977228; doi:10.1186/s12870-020-2255-3)
Supplement: Supplementary file 5 — Additional file 5. Bioinformatics analysis of NtZIP5B subcellular localization [file 12870_2020_2255_MOESM5_ESM.pdf]

Additional file 5:

Bioinformatics analysis to indicate the subcellular localization of NtZIP5A and NtZIP5B proteins.

Bioinformatic analysis of NtZIP5A and NtZIP5B proteins was performed based on ProtComp v. 9.0 (http://www.softberry.com/berry.phtml?topic=protcomppl&group=programs&subgroup=proloc).

The sequence of NtZIP5A

>NP_001312674.1_NtZIP5A

MTKLEKVIFWYILLLLPAIVLGECTCDSEDEERNKTEALKYKMVAIASILIASAIGVCIPVLGKAIPALSPEKNF
FFIIKAFAAGVILATGFIHVLPDAFESLTSPCLKENPWGNFPFSGFIAMVSAMGTLMVDTYATSYFSNKNDTKNG
LVAQSGDEGGAIHVHSHGSASLMGDSSSELLRYRVVSQVLEMGIIVHSVIIGIALGASESPKTIRPLVAALTFHQ
FFEGMGLGGCIAQAKFKTRAVAIMALFFSLTTPVGIAIGLGITNVYDENSPTALIVEGVFNSASAGILIYMALVD
FLAADFMHPRMQGNGKLQLGANISLLLGAGLMALIAKWA

```
5724 multiple located sequences are accepted
ProtComp Version 9.0. Identifying sub-cellular location (Plant)
Seq name: NP_001312674.1_NtZIP5A, Length=339
Significant similarity in Location DB - Plasma membrane
Database sequence: AC=Q6L8G0 Location:Plasma membrane  DE  Zinc transporter
5;||37
Score=89, Sequence length=354, Alignment length=305
Predicted by Neural Nets - Extracellular (Secreted) with score    0.9
******** Transmembrane segments are found: .-41:67o..o74:98-..-113:134-..-
192:210+..+226:240+..+247:272-..-283:305+.
******** Potential GPI-anchor in position 323 is found
Integral Prediction of protein location: Plasma membrane with score    8.7
Location weights:     LocDB / PotLocDB / Neural Nets / Pentamers / Integral
 Nuclear                0.0 /      0.0 /       0.00 /      0.00 /     0.00
 Plasma membrane       10.0 /      1.6 /       0.88 /      1.71 /     8.68
 Extracellular          0.0 /      0.0 /       0.88 /      0.02 /     0.14
 Cytoplasmic            0.0 /      0.0 /       0.00 /      0.04 /     0.00
 Mitochondrial          0.0 /      0.0 /       0.00 /      1.89 /     0.04
 Endoplasm. retic.      0.0 /      0.0 /       0.00 /      2.19 /     0.00
 Peroxisomal            0.0 /      0.0 /       0.88 /      0.00 /     0.04
 Golgi                  0.0 /      0.0 /       0.37 /      0.18 /     0.08
 Chloroplast            0.0 /      1.4 /       0.00 /      0.15 /     1.02
 Vacuolar               0.0 /      0.0 /       0.00 /      0.00 /     0.00
```

The sequence of NtZIP5B

>XP_016449056.1_NtZIP5B

MTKLEKVVFWYILLLLPAIVLGECTCDSEDEERNKPEALKYKMVAIASILIASAIGVCIPVLGKAIPALSPEKNF
FFIIKAFAAGVILATGFIHVLPDAFESLTSPCLKENPWGNFPFSGFIAMVSAMGTLMVDTYATSYFSNKNDTKNG
LVAQSGDEGGAIHVHSHGHAHGSSSLLGDSSSELLRYRVVSQVLEMGIIVHSVIIGIALGASESPKTIRPLVGAL
TFHQFFEGMGLGGCIAQAKFKTRAVAIMALFFSLTTPVGIAIGLGITNVYDENSPTALIVEGVFNSASAGILIYM
ALVDFLAADFMHPRMQGNGKLQLGANVSLLLGAGLMALIAKWA

Seq name: XP_016449056.1_**NtZIP5B**, Length=343
Significant similarity in Location DB - Plasma membrane
Database sequence: AC=Q6L8G0 Location:Plasma membrane DE Zinc transporter 5;||37
Score=87, Sequence length=354, Alignment length=300
Predicted by Neural Nets - Extracellular (Secreted) with score 0.9
******** Transmembrane segments are found: .-41:67o..o74:98-..-113:134-..-196:214+..+230:244+..+251:276-..-287:309+.
******** Potential GPI-anchor in position 327 is found
Integral Prediction of protein location: Plasma membrane with score 8.7

Location weights:	LocDB /	PotLocDB /	Neural Nets /	Pentamers /	Integral
Nuclear	0.0 /	0.0 /	0.00 /	0.00 /	0.00
Plasma membrane	10.0 /	1.6 /	0.88 /	1.44 /	8.69
Extracellular	0.0 /	0.0 /	0.88 /	0.00 /	0.14
Cytoplasmic	0.0 /	0.0 /	0.00 /	0.06 /	0.00
Mitochondrial	0.0 /	0.0 /	0.00 /	2.18 /	0.05
Endoplasm. retic.	0.0 /	0.0 /	0.00 /	2.04 /	0.00
Peroxisomal	0.0 /	0.0 /	0.88 /	0.00 /	0.04
Golgi	0.0 /	0.0 /	0.37 /	0.22 /	0.07
Chloroplast	0.0 /	1.4 /	0.00 /	0.24 /	1.02
Vacuolar	0.0 /	0.0 /	0.00 /	0.00 /	0.00

As a control the following transport proteins were included into analysis:

(1) **NtZIP1-like** - targeted to the plasma membrane (Papierniak et al., 2018)

>XP_016507999.1 PREDICTED: zinc transporter 1-like [Nicotiana tabacum]

MNNHNVQVCSYCYKAVVLTCLVILVFAPGISGECTCNIKVDQPRNTKNSSDSLRYRLISIVSILIAGAIGVSLPL
LARKIEALRPENDIFFMIKAFAAGVILATGFIHILPDAFQTLTSPCLQGMDPWGKFPFTGFFAMIASIGCLMIDT
FATSFYQNRHFHIAKQVNIVDEEAARDDIQHSHSHASHVAHGATHSIGSDQELILSENIRNRIISQVLELGILVH
SIIIGVSLGASQNTEMIKPLLVALSFHQFFEGMGLGGCISQAKFKSTSTAIMSVLFSLTTPAGIGIGIGISRVYN
AHSSISLIVEGILNSASSGILIYMALVDILASDFMNPRMQNNVRLLCGAHISLLLGAGCMSVMAKWA

Seq name: XP_016507999.1 PREDICTED: zinc transporter 1-like [Nicotiana tabacum], Length=367
Significant similarity in Location DB - Plasma membrane
Database sequence: AC=A3BI11 Location:Plasma membrane DE Zinc transporter 8;||37
Score=81, Sequence length=391, Alignment length=298
Predicted by Neural Nets - Extracellular (Secreted) with score 0.9
******** Transmembrane segments are found: .+55:78-..-88:112o..o130:156-..-219:238o..o254:268+..+275:300-..-311:333+.
******** Potential GPI-anchor in position 342 is found
Integral Prediction of protein location: Plasma membrane with score 8.7

Location weights:	LocDB /	PotLocDB /	Neural Nets /	Pentamers /	Integral
Nuclear	0.0 /	0.0 /	0.00 /	0.00 /	0.00
Plasma membrane	10.0 /	1.6 /	0.88 /	1.53 /	8.66
Extracellular	0.0 /	0.0 /	0.88 /	0.81 /	0.16
Cytoplasmic	0.0 /	0.0 /	0.00 /	0.16 /	0.00
Mitochondrial	0.0 /	0.0 /	0.00 /	2.00 /	0.05
Endoplasm. retic.	0.0 /	0.0 /	0.00 /	1.51 /	0.00
Peroxisomal	0.0 /	0.0 /	0.88 /	0.00 /	0.04
Golgi	0.0 /	0.0 /	0.37 /	0.00 /	0.06
Chloroplast	0.0 /	1.4 /	0.00 /	0.36 /	1.04
Vacuolar	0.0 /	0.0 /	0.00 /	0.00 /	0.00

(2) **CsMTP1** from *C. sativus* targeted to the tonoplast, (Migocka et al, 2015)

```
>ABS12731.1 metal tolerance protein [Cucumis sativus]
MEVQDHGHIIEVCGDVQAVGPSIVGSKICGDAPSCGFSDAKNSSKDAKERSASMRKLCIAVVLCIVFMSV
EVVGGIKANSLAILTDAAHLLSDVAAFAISLFSLWASGWEATPRQSYGFFRIEILGALVSIQMIWLLAGI
LVYEAIVRLINGPGEVKGFLMFAVSTFGLVVNIAMALLLGHEHGHAHSHGHNHGHGEHDHGHGSHEHGEE
DHRHRHGISVTMHHHHHEEKRAASDGVEHHHHHHHHKHKESTTVPLLDSSQKVTKAQKKQRNINVQGAYL
HVLGDSIQSIGVMIGGAIIWYKPEYMILDLICTLIFSAIVLCTTIQMLRNILEVLMESTPREVDATKLEK
GLCEMEEVVAIHELHIWAITVGKILLACHVIIKPEANADMVLDKVIEYIRREYNISHVTIQIERQ
```

```
Seq name: ABS12731.1 metal tolerance protein [Cucumis sativus], Length=415
Significant similarity in Location DB - Vacuole
Database sequence: AC=Q2HJ10 Location:Vacuole  DE  Zinc transporter 2;
Score=77, Sequence length=372, Alignment length=289
Predicted by Neural Nets - Extracellular (Secreted) with score    0.9
Integral Prediction of protein location: Membrane bound Vacuolar with score
9.0
Location weights:     LocDB / PotLocDB / Neural Nets / Pentamers / Integral
 Nuclear                0.0 /      0.0 /        0.00 /      0.00 /     0.00
 Plasma membrane        0.0 /      0.0 /        0.88 /      0.23 /     0.26
 Extracellular          0.0 /      0.0 /        0.88 /      2.69 /     0.30
 Cytoplasmic            0.0 /      0.0 /        0.00 /      1.14 /     0.00
 Mitochondrial          0.0 /      0.0 /        0.00 /      1.22 /     0.38
 Endoplasm. retic.      0.0 /      0.0 /        0.00 /      0.38 /     0.04
 Peroxisomal            0.0 /      0.0 /        0.88 /      0.00 /     0.05
 Golgi                  0.0 /      0.0 /        0.37 /      0.01 /     0.00
 Chloroplast            0.0 /      0.0 /        0.00 /      0.05 /     0.00
 Vacuolar              10.0 /      0.0 /        0.00 /      0.01 /     8.97
```

(3) **AtMTP3** from *A. thaliana* targeted to the tonoplast, (Arrivault et al, 2006)

```
>NP_191440.2 metal tolerance protein A2 [Arabidopsis thaliana]
MVTPKLHLDLSLTKKVSYLFVSRQTLYRLSSSCLASLFLSFDAFLIARSCFFFQMKDHIHEHDHMVQICGEVSSG
ETSLVGIKKTCGEAPCGFSDAKTSSIEAQERAASMRKLLIAVLLCAIFIVVEVVGGIKANSLAILTDAAHLLSDV
AAFAISLFSLWASGWKANPQQSYGFFRIEILGALVSIQMIWLLAGILVYEAIVRLNNGSGEVEGSLMFAVSAVGL
LVNIAMAILLGHDHGHGHGHSHDNGHGHSHDHGHGIAATEHHHDSGHDESQLSDVLIEQKKQRNVNIQGAYLHVL
GDSIQSVGVMIGGAIIWYKPEWKILDLICTLVFSVIVLGTTIGMLRNILEVLMESTPREIDPTMLEKGVCEIEEV
VAVHELHIWAITVGKLLLACHVKIRPEAEADMVLDKIIDYIKREHNISHVTIQIERQ
```

```
Seq name: NP_191440.2 metal tolerance protein A2 [Arabidopsis thaliana],
Length=432
Significant similarity in Location DB - Vacuole
Database sequence: AC=Q2HJ10 Location:Vacuole  DE  Zinc transporter 2;
Score=78, Sequence length=372, Alignment length=293
Predicted by Neural Nets - Extracellular (Secreted) with score    0.9
Integral Prediction of protein location: Membrane bound Vacuolar with score
8.9
Location weights:     LocDB / PotLocDB / Neural Nets / Pentamers / Integral
 Nuclear                0.0 /      0.0 /        0.00 /      0.00 /     0.00
 Plasma membrane        0.0 /      0.0 /        0.87 /      0.47 /     0.06
 Extracellular          0.0 /      0.0 /        0.87 /      1.36 /     0.35
 Cytoplasmic            0.0 /      0.0 /        0.00 /      0.62 /     0.00
 Mitochondrial          0.0 /      0.0 /        0.00 /      1.75 /     0.47
 Endoplasm. retic.      0.0 /      0.0 /        0.00 /      1.36 /     0.14
```

Peroxisomal	0.0 /	0.0 /	0.87 /	0.00 /	0.05
Golgi	0.0 /	0.0 /	0.39 /	0.33 /	0.00
Chloroplast	0.0 /	0.0 /	0.00 /	0.00 /	0.00
Vacuolar	10.0 /	0.0 /	0.00 /	0.00 /	8.94

References:

Arrivault S, Senger T, Krämer U. 2006. The *Arabidopsis* metal tolerance protein AtMTP3 maintains metal homeostasis by mediating Zn exclusion from the shoot under Fe deficiency and Zn oversupply. Plant Journal **46**, 861-79.

Fu XZ, Zhou X, Xing F, Ling LL, Chun CP, Cao L, Aarts MGM, Peng LZ. 2017. Genome-wide identification, cloning and functional analysis of the Zinc/Iron-Regulated transporter-Like Protein (*ZIP*) gene family in Trifoliate Orange (*Poncirus trifoliata* L. Raf.). *Frontiers in Plant Science.* **19**, 588.

Migocka M, Kosieradzka A, Papierniak A, Maciaszczyk-Dziubinska E, Posyniak E, Garbiec A, Filleur S. 2015. Two metal-tolerance proteins, MTP1 and MTP4, are involved in Zn homeostasis and Cd sequestration in cucumber cells. Journal of Experimental Botany. **66**, 1001-1015.

Papierniak A, Kozak K, Kendziorek M, Barabasz A, Palusińska M, Tiuryn J, Paterczyk B, Williams LE, Antosiewicz DM. 2018. Contribution of NtZIP1-Like to the regulation of Zn homeostasis. Frontiers in Plant Science. **16**, 185.
