# Supplementary material for: Zn/Cd status-dependent accumulation of Zn and Cd in root parts in tobacco is accompanied by specific expression of ZIP genes
Source: BMC Plant Biol. 2020 Jan 22;20:37. doi: 10.1186/s12870-020-2255-3 (PMC6977228; doi:10.1186/s12870-020-2255-3)

Additional file 6:

Changes in the expression of tobacco *ZIP* genes in the apical, middle and basal root part, which accompany Cd-dependent stimulation of Zn translocation to shoots;

a) Pairwise combinations of Zn and Cd concentration used for comparison of the efficiency of Zn translocation to shoots; experimental variant with Cd where the stimulation of Zn translocation occurred (underlined) was compared with the reference combination without Cd;
b) Zn shoot/root concentration ratio demonstrates the efficiency of Zn translocation to shoots;
c) Graphical representation of changes in the expression level of tobacco *ZIP* genes in the apical, middle and basal root part;
   Arrows indicate increase or decrease in the expression in the presence of Cd, relative to the combination without Cd;

(a)

0 µM Zn + 0 µM Cd	1 µM Zn + 0 µM Cd	1 µM Zn + 0 µM Cd
0 µM Zn + 0.25 µM Cd	1 µM Zn + 0.25 µM Cd	1 µM Zn + 1 µM Cd

(b)

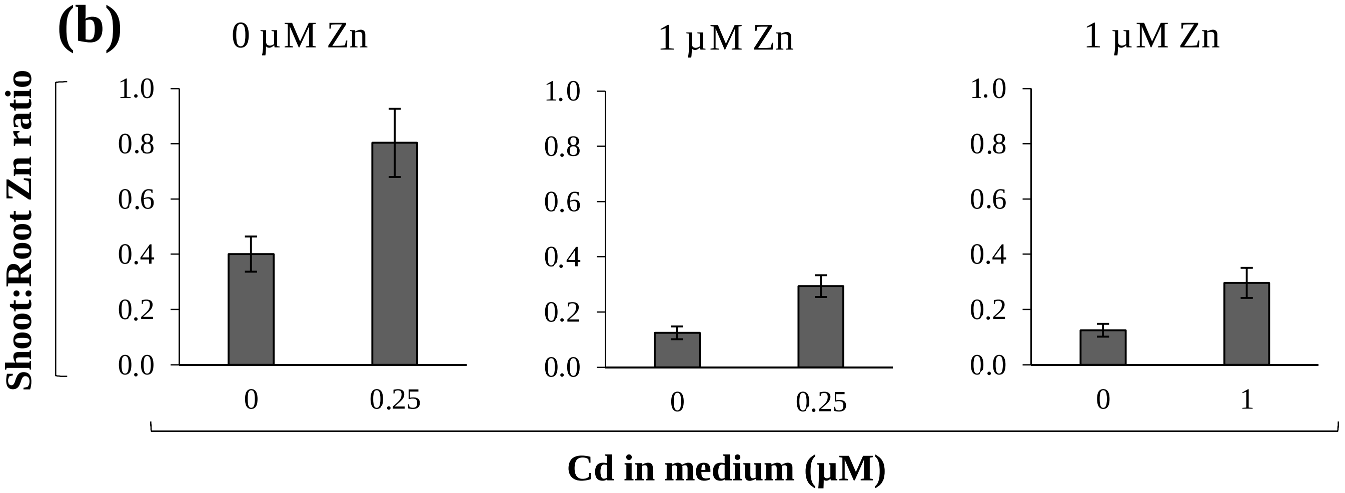

(c)

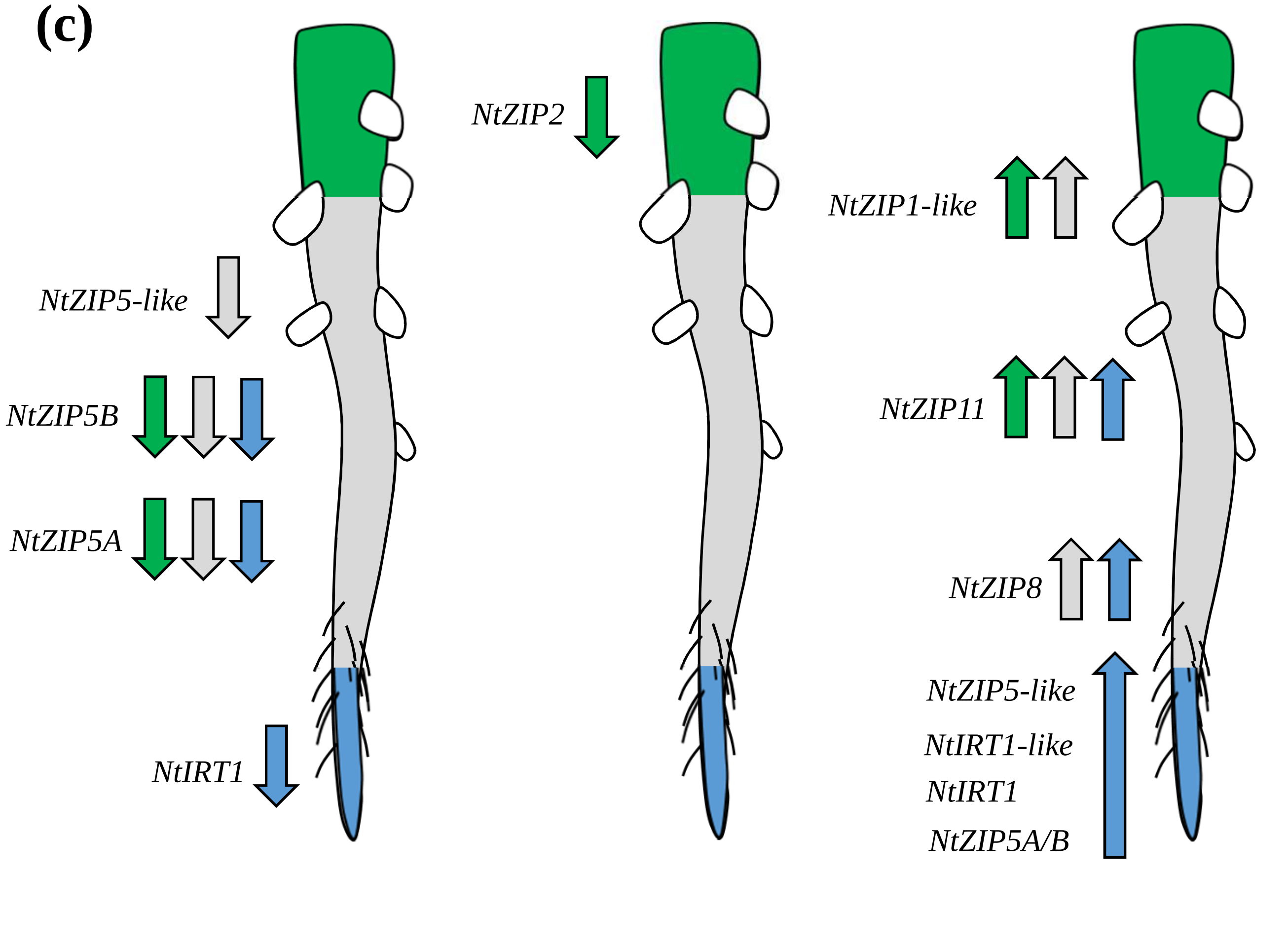

Supplement: Supplementary file 6 — Additional file 6. Graphical presentation of root part-specific changes in ZIPs expression [file 12870_2020_2255_MOESM6_ESM.pdf]
