# Supplementary material for: Zn/Cd status-dependent accumulation of Zn and Cd in root parts in tobacco is accompanied by specific expression of ZIP genes
Source: BMC Plant Biol. 2020 Jan 22;20:37. doi: 10.1186/s12870-020-2255-3 (PMC6977228; doi:10.1186/s12870-020-2255-3)
Supplement: Supplementary file 7 — Additional file 7. Stability of PP2A [file 12870_2020_2255_MOESM7_ESM.pdf]

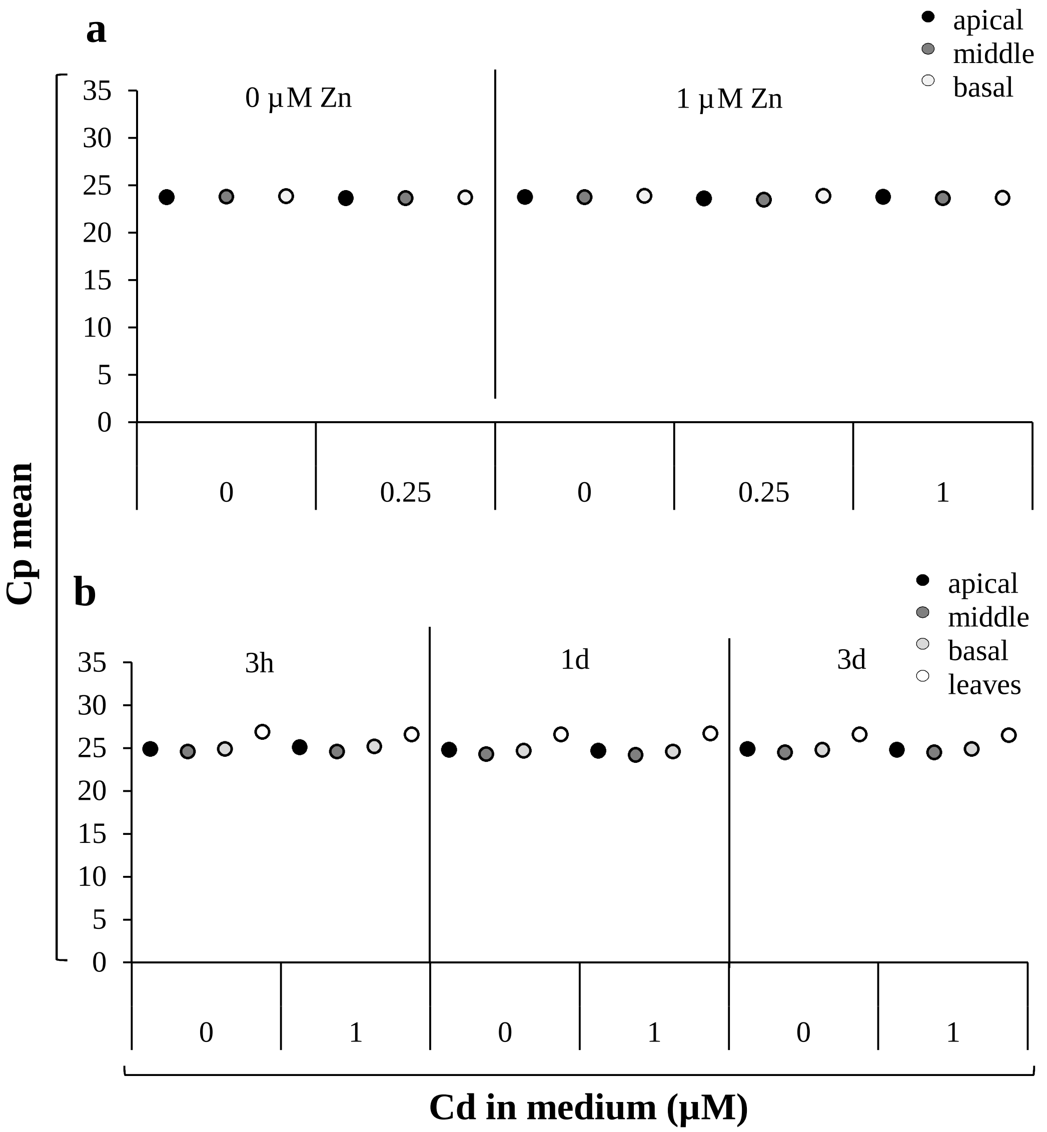

Additional file 7 :

RNA transcription levels of PP2A gene, presented as Cp mean values in plant samples collected from performed experiments: (a) in the apical, middle and basal root parts of plants grown at control conditions and exposed for 17 days to combinations of Zn (0; 1 µM) and Cd (0; 0.25 µM; 1 µM) concentrations; (b) in the apical, middle, basal root parts and leaves of plants grown at control conditions and exposed for 3 hours, 1 day and 3 days to combinations of Zn (1 µM) and Cd (0; 1 µM) concentrations;
