# Supplementary material for: Zn/Cd status-dependent accumulation of Zn and Cd in root parts in tobacco is accompanied by specific expression of ZIP genes
Source: BMC Plant Biol. 2020 Jan 22;20:37. doi: 10.1186/s12870-020-2255-3 (PMC6977228; doi:10.1186/s12870-020-2255-3)
Supplement: Supplementary file 8 — Additional file 8. Primer sequences used for expression analysis, cloning of NtZIP5B and promoter isolation [file 12870_2020_2255_MOESM8_ESM.pdf]

Additional file 8: Primer sequences used for expression analysis

Gene name	Primer For	Primer Rev	Products on target templates	Lenght of amplicon (bp)
NtIRT1	CGCAATAACAACTCCATTCG	AAGCCATATAGATCAGAAGGC	AB263746.1	134
NtIRT1-like	CTTCTTCGCAGTAACAACC	AGCCATGTAAATAAGAAGACC	XM_016611068.1	139
NtZIP1-like	TGCTGCTGGTGTCATTCTAG	GCTGGCATGACTATGACTGTG	XM_016652513.1	274
NtZIP2	CACCATGTTTAGTGACTGC	CTTGAGAAAAGGATTTGCTTCC	XM_016617597.1	136
NtZIP4A	CTGTTTCCAATACCACCTGT	GCTTCTTGCCAACTAATGGA	XM_016647965.1	150
NtZIP4B	ACTGACTCTAATCTCTTTCTTGC	ATGGCGATAAAACCAGCG	XM_016586154.1	161
NtZIP5A [# *]	TGGGAACTCTTATGGTGGAT	CTGAACCATGTGAATGAACATGA	NM_001325745.1	126
NtZIP5B [#]	GGGAACTCTAATGGTGGAC	ATGTGCATGGCCATGTG	XM_016594002.1	118
NtZIP5-like	TCTGCGAAAAATGGTGTG	GAAGGAGCTCGGAATCAG	XM_016594002.1	118
NtZIP8	GGTTGTGCCATTAGAAGAGG	AGTTAATGCCGCTACAAGG	XM_016603305.1, XM_016586286.1	163
NtZIP11	CTGACACAGATTCCGACTCA	CACAATCAGCCAACATAGTAAGC	XM_016644574.1	321
NtPP2A	GCACATTCATTCAGTTTGAACC	GTAGCATATAAAGCAGTCAGC	NM_001325282.1	142

[#] nucleotide alignment of *NtZIP5A* / *NtZIP5B* with marked positions of starters used for expression analysis of both genes are given in the Additional file 2c; Specificity of primers used for expression analysis of *NtZIP4A* and *NtZIP4B* are shown in the Additional file 2d.

* *NtZIP1* identified by Sano et al. (2012) was here renamed *NtZIP5A* (detailes in the Additional file 2; Figure 8).

[**ref**: Sano T, Yoshihara T, Handa K, Sato MH, Nagata T, Hasezawa S. Metal ion homeostasis mediated by Nramp transporters in plant cells - focused on increased resistance to iron and cadmium ion. In: Weigert R, editor. Crosstalk and integration of membrane trafficking pathways. Rijeka, Shanghai: INTECH; 2012. p.214–228].

Primer sequences used for cloning *NtZIP5B* and promoters

Primer name	Primer sequence
ZIP5B-ORF-start	CACCATGACAAAGTTAGAAAAAGTAGTTTTTGGTACATTCTCTTGC
ZIP5B-ORF-end-STOP	TTAAGCCCATTTGGCTATGAGAGCCATGAG
promZIP5A-F	CACCGAATATCATGGAGGAGGTTATGG
PromZIP5A-R	CATGACTCTAGGTATAGAAAATGA
PromZIP5B-F	CACCGGTCATATTTTCAGCCCAAAC
PromZIP5B-R	CATGACTCTAGGTTTAGAAAACC
